# Supplementary material for: Comparative Analysis of Enzymatic Transglycosylation Using E. coli Nucleoside Phosphorylases: A Synthetic Concept for the Preparation of Purine Modified 2′-Deoxyribonucleosides from Ribonucleosides
Source: Int J Mol Sci. 2022 Mar 3;23(5):2795. doi: 10.3390/ijms23052795 (PMC8911250; doi:10.3390/ijms23052795)
Supplement: Supplementary file 1 [file ijms-23-02795-s001.zip › ijms-1613376-supplementary.pdf]

Comparative analysis of enzymatic transglycosylation using nucleoside phosphorylases *E.coli*: a synthetic concept for the preparation of purine modified 2′-deoxyribonucleosides from ribonucleosides.

Mikhail S. Drenichev[1], Vladimir E. Oslovsky[1], Anastasia A. Zenchenko[1], Claudia V. Danilova[1], Mikhail A. Varga[1], Roman S. Esipov[2], Dmitry D. Lykoshin[2], Cyril S. Alexeev[a1]. *

[1]*Engelhardt Institute of Molecular Biology, Russian Academy of Sciences, Vavilov str. 32, Moscow, 119991 Russia.*
[2]*Laboratory of Biopharmaceutical Technologies, Shemyakin-Ovchinnikov Institute of Bioorganic Chemistry, Russian Academy of Sciences, Ulitsa Miklukho-Maklaya, 16/10, GSP-7, Moscow, 117997, Russian Federation*

[*]Correspondance to micelle@mail.ru, cyril.aleks@gmail.com.

Reagents and solutions

All chemicals and solvents were of analytical grade or higher and purchased, if not stated otherwise, from Sigma–Aldrich (United States).

All solutions were prepared in water purified using Milli-Q® water ultrafiltration station (Merck Millipore, United States). pH values were determined with microprocessor-based pH (mV- C) bench meter 211 (Hanna Instruments, Germany), equipped with HI1131B double junction combination pH electrode and HI7662 stainless steel temperature sensor for pH compensation.

K,Na-phosphate buffer solution (50 mM, pH 7.5). 50 mL of 0.1 M KH_2PO_4 and 40.9 mL of 0.1 M NaOH were mixed in 100 mL volumetric flask. The pH value of the solution was adjusted to 7.5 by careful addition of 0.1 M NaOH. The overall volume was brought to 100 mL by addition of water. The resulting solution had a buffer capacity equal to β 0.016.

K,Na-phosphate buffer solution (5 mM, pH 7.5). For analytical reactions was used 50 mM Tris-HCl buffer diluted 10 times.

Tris – HCl buffer solution (50 mM, pH 7.5). 50 mL of 0.1 M tris(hydroxymethyl)aminomethane solution and 40.3 mL 0.1 M HCl solution were mixed in 100 mL volumetric flask. The pH value was adjusted to 7.5 by careful addition of 0.1 M HCl solution. The overall volume was brought to 100 mL by addition of water. The resulting solution had a buffer capacity equal to β 0.017.

The following solutions were used in transglycosylation reactions and their analysis using HPLC, UV-spectroscopy.

Stock 1 mM solutions of compounds were prepared by dissolving 0.025 mmol of compound in 50 mM Tris – HCl buffer or deionized water in 25 mL volumetric flask (0.025 mmol corresponds to 10.2 mg of 7-Me-dGuo hydroiodide, 6.3 mg of dAdo, 6.3 mg of dIno, 6.7 mg of dGuo, and 6.00 mg of *N*^{6}-Bn-2-NH$_2$-Ade).

Stock 2 mM solutions of compounds were prepared by dissolving 0.05 mmol of compound in deionized water in 25 mL volumetric flask (0.05 mmol corresponds to 6.8 mg of Ade, 6.8 of mg of Hyp, 6.3 mg of 5-Me-Ura (Thy), 11.4 mg of dUrd, 5.6 mg of Ura).

Enzymes. A purine nucleoside phosphorylase *E. coli* (PNP, Sigma-Aldrich N2415) and thymidine phosphorylase *E. coli* (TP, Sigma-Aldrich T2807) were purchased from Sigma–Aldrich (United States).

Absorbance spectra were detected and recorded by means of Cary 3 Bio UV/VIS spectrophotometer (Varian, Australia) using semi-Micro absorption cuvettes 104-QS (chamber volume 1400 μL, pathlength 10 mm) and Macro absorption cuvettes standard cells 110-QS (chamber volume 3500 μL, pathlength 10 mm) made of Suprasil® quartz (Hellma, Germany).

HPLC analysis: Analytical HPLC analysis was run using Akvilon (Russia) HPLC system (2×Stayer pumps (2nd series), Stayer MS16 dynamic mixer and Stayer 104M UV-Vis detector).
The analysis was performed on the following HPLC columns and conditions:
a) 4.6×150mm column (5 μm, Cosmosil 5C18-MS-II, approx. 120 Å, Part No 38019-81, Nacalai Tesque, Inc. (Japan)) equipped with EC security guard (4.0×3 мм, 5 μm, C_{18} Part No AJ0-4287, Phenomenex (United States)) for enzymatic transglycosilation reactions with Thd and Ade (Ura), dUrd and 5-Me-Ura, dAdo and Ura, 7-Me-dGuo and Ura (Hyp), *N*^{6}-Bn-2-NH$_2$-Ade. HPLC analysis was run in a linear acetonitrile gradient in 0.06% (v/v) TFA/deionized water from 2 to 12% for 10 min (flushing with 12-80 % acetonitrile-0.06% TFA/deionized water for 10-10.1 min, then 80-2% for 10.1-10.8 min) at flow rate 1 ml/min with UV detection at wavelength 260 nm for Thd and Ade (Ura), dUrd and 5-Me-Ura, dAdo and Ura, 7-Me-dGuo and Ura (Hyp). For the reaction 7-Me-dGuo and *N*^{6}-Bn-2-NH$_2$-Ade HPLC analysis conditions was run in a linear acetonitrile gradient in 0.06% (v/v) TFA/deionized water from 2 to 30% for 15 min (flushing with 12-80 % acetonitrile-0.06% TFA/deionized water for 15-15.1 min, then 80-2% for 15.1-15.8 min) at flow rate 1 ml/min with UV detection at 283 nm, injection volume 20 μL.
b) 4.6×250mm column (5 μm, Cosmosil 5CN-MS, approx. 300 Å, Part No 38236-31, Nacalai Tesque, Inc. (Japan)) equipped with Rheodyne inline filter (2 μm) for enzymatic transglycosilation reactions with dAdo and Hyp, dIno and Ade, dGuo and Ade (Hyp). HPLC analysis was run in a linear acetonitrile gradient in 10 mM NaOAc/deionized water from 2 to 12% for 10 min (flushing with 12-

80 % acetonitrile-10 mM NaOAc/deionized water for 10-10.1 min, then 80-2% for 10.1-10.8 min) at flow rate 1 ml/min with UV detection at wavelength 260 nm, injection volume 20 μL.

The conditioning and equilibration of the chromatographic column was conducted for at least half an hour after each measurement.

HPLC purification: HPLC purificaton was run using Akvilon (Russia) HPLC system (2×Stayer pumps (2nd series), Stayer MS16 dynamic mixer and Stayer 104M UV-Vis detector) and semi-prep 10.0×250mm column (5 μm, Cosmosil 5C18-MS-II, approx. 120 Å, Part No 38023-11, Nacalai Tesque, Inc. (Japan)) equipped with EC security guard (10.0×10 мм, C_{18} Part No AJ0-7221, Phenomenex (United States)) for enzymatic transglycosilation reactions with 7-Me-dGuo and N^6-Bn-2-NH_2-Ade. HPLC analysis was run in a linear acetonitrile gradient in deionized water from 2 to 30% for 15 min (flushing with 30-80 % acetonitrile-deionized water for 15-15.1 min, then 80-2% for 15.1-15.8 min) at flow rate 3 ml/min with UV detection at wavelength 283 nm, injection volume 500 μL.

Important!: The product was eluted after flushing step in gradient program.

Calibration coefficients (α) were calculated using equation:

$$C_{t=0} = \alpha \times S_{t=0},$$

were t = 0 min refers to HPLC run immediately after mixing a substrates sample solution with the enzyme(s) solution. The HPLC data at t = 0 was used for calibration coefficients (α) calculations. Using these values, the equilibrium concentrations were calculated, and the data are presented below, in figures.

<u>2´-deoxyadenosine from thymidine (Thd+Ade+P_i, analytical method)</u>

To a reaction sample solution (1 mL, Table S1) were added 1 U of TP *E. coli* and 1 U of PNP *E. coli*. The reaction mixture was incubated at 37°C, pH 7.5, and was monitored by HPLC.

Table S1. Volumes of stock and buffer solutions taken for 1 mL of a rection mixture (pH 7.5).

2 mM Thd stock solution	2 mM Ade stock solution	5 mM phosphate	50 mM Tris - HCl	C_{Thd}:C_{Ade}:C_P
100 μL	150 μL	40 μL	710 μL	1:1.5:1
150 μL	100 μL	10 μL	740 μL	1.5:1:0.25
150 μL	100 μL	40 μL	710 μL	1.5:1:1
300 μL	100 μL	40 μL	560 μL	3:1:1

HPLC-analysis of transglycosylation: Cosmosil 5C18-MS-II, 4.6*150 mm, 5µm, 120 Å, (Nacalai Tesque, Inc. (Japan)), 20ºC, elution in modified mobile phase: linear gradient of acetonitrile in 0.06% TFA/deionized water from 2 to 12% for 10 min (flushing with 12-80 % acetonitrile-0.06% TFA/deionized water for 10-10.1 min, then 80-2% for 10.1-10.8 min) at flow rate 1 ml/min with UV detection at wavelength 260 nm, injection volume 20 µL.

Fugure S1.1. **1:1.5:1 ratio**

a) $t_{initial}$

Thd+Ade+P_i – 1:1.5:1 – t_0

№	RT, min	Area, mV*сек	Equilibrium concentration, µM	Name
**1**	3.36	4551.03	300.00	Ade
**2**	9.26	2055.08	200.00	Thd

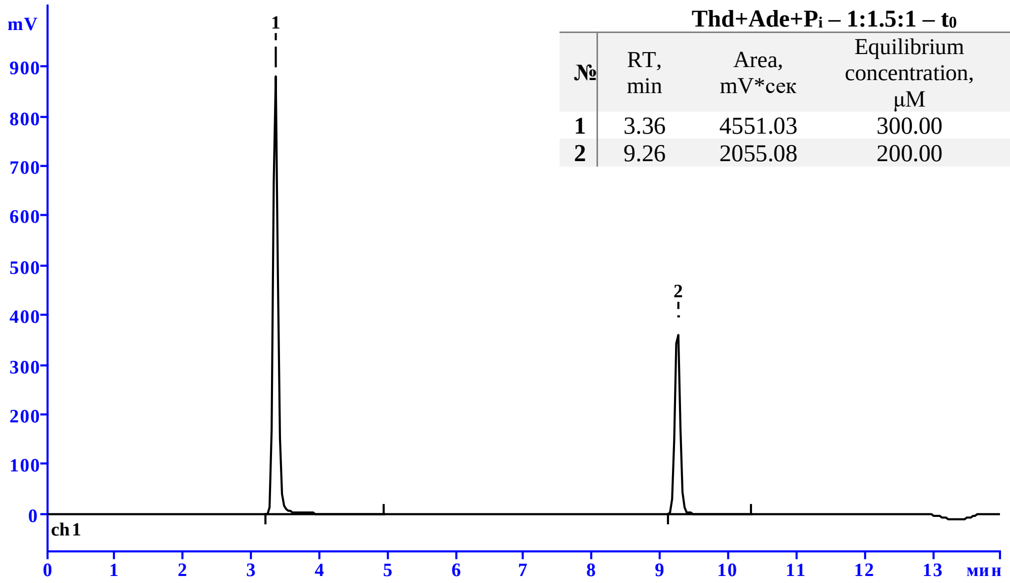

b) $t_{equilibrium}$

Thd+Ade+P_i – 1:1.5:1– t $_{equilibrium}$

№	RT, min	Area, mV*сек	Equilibrium concentration, µM	Name
**1**	3.25	1946.42	128.31	Ade
**2**	5.82	1612.50	181.57	Thy
**3**	7.19	2902.10	171.69	dAdo
**4**	9.22	189.36	18.43	Thd

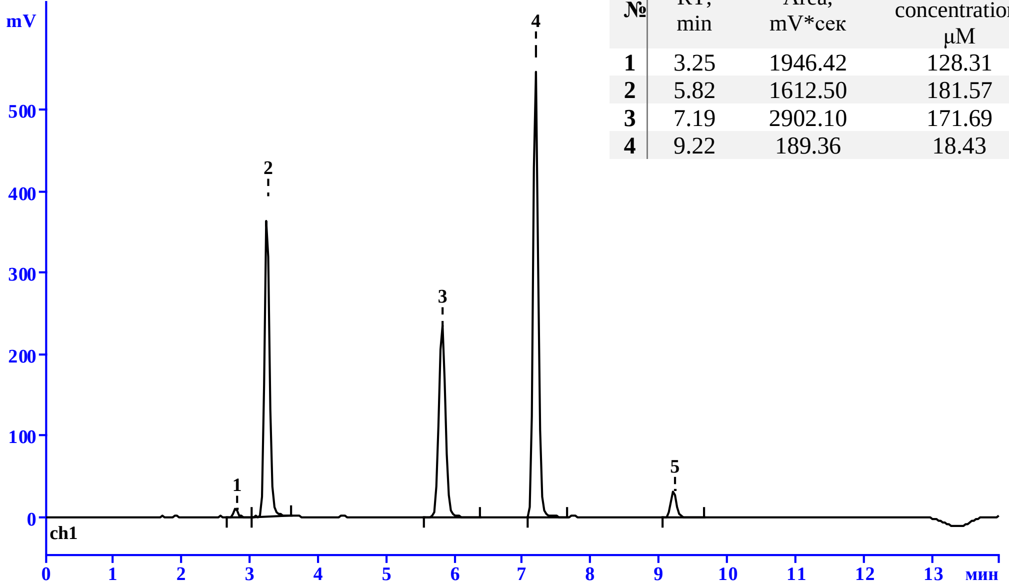

Fugure S1.2. **1.5:1:0.25 ratio**

a) $t_{initial}$

Thd+Ade+P_i – 1.5:1:0.25 – t_0

№	RT, min	Area, mV*сек	Equilibrium concentration, µM	Name
**1**	3.38	3125.91	200.00	Ade
**2**	9.26	3078.26	300.00	Thd

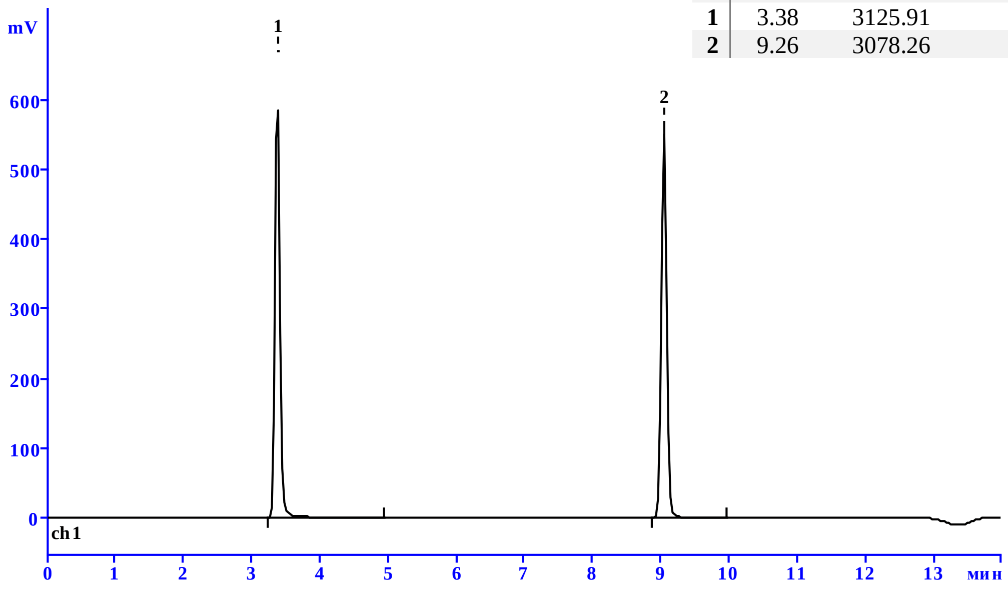

b) $t_{equilibrium}$

Thd+Ade+P_i – 1.5:1:0.25– $t_{equilibrium}$

№	RT, min	Area, mV*сек	Equilibrium concentration, µM	Name
**1**	3.42	551.42	35.28	Ade
**2**	5.97	1964.07	226.99	Thy
**3**	7.25	2847.16	164.72	dAdo
**4**	9.20	749.10	73.01	Thd

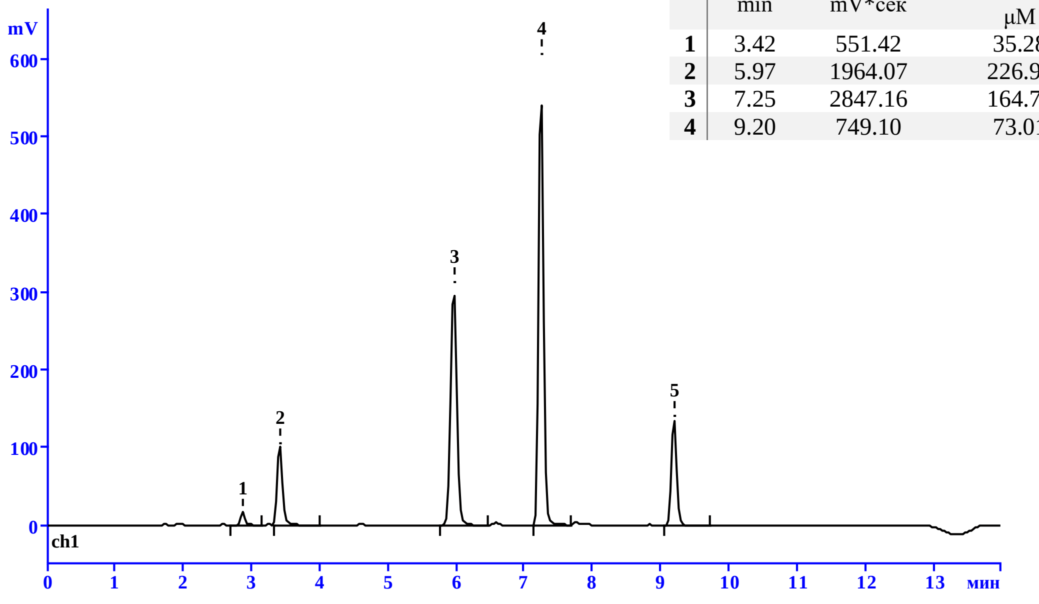

Fugure S1.3. **1.5:1:1 ratio**

a) t

a) $t_{initial}$

Thd+Ade+P_i – 1.5:1:1 – t_0

№	RT, min	Area, mV*сек	Equilibrium concentration, µM	Name
**1**	3.36	3061.05	200.00	Ade
**2**	8.97	3177.00	300.00	Thd

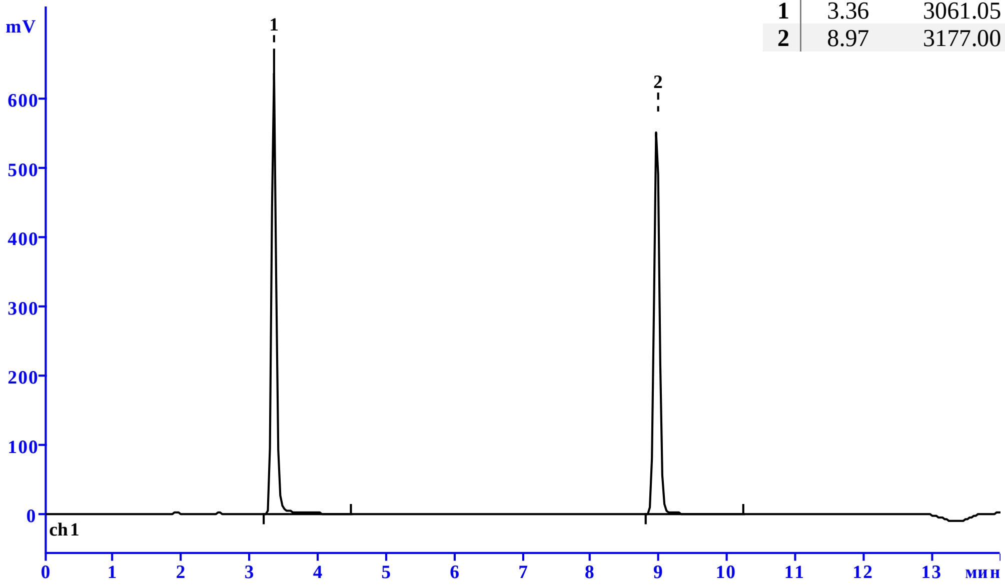

b) $t_{equilibrium}$

Thd+Ade+P_i – 1.5:1:1– t $_{equilibrium}$

№	RT, min	Area, mV*сек	Equilibrium concentration, µM	Name
**1**	3.37	495.90	32.40	Ade
**2**	5.75	1960.56	213.39	Thy
**3**	7.03	2914.09	167.60	dAdo
**4**	9.12	917.18	86.61	Thd

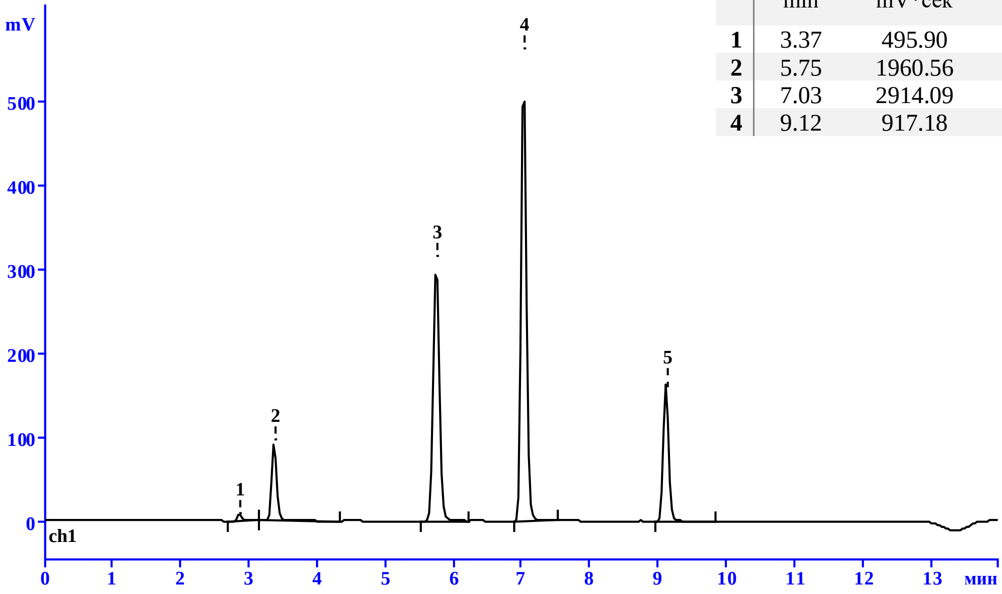

Fugure S1.4. **3:1:1 ratio**

a) t_{initial}

Thd+Ade+P_i – 3:1:1 – t_0

№	RT, min	Area, mV*сек	Equilibrium concentration, µM	Name
**1**	3.36	3130.56	200.00	Ade
**2**	8.96	6409.81	600.00	Thd

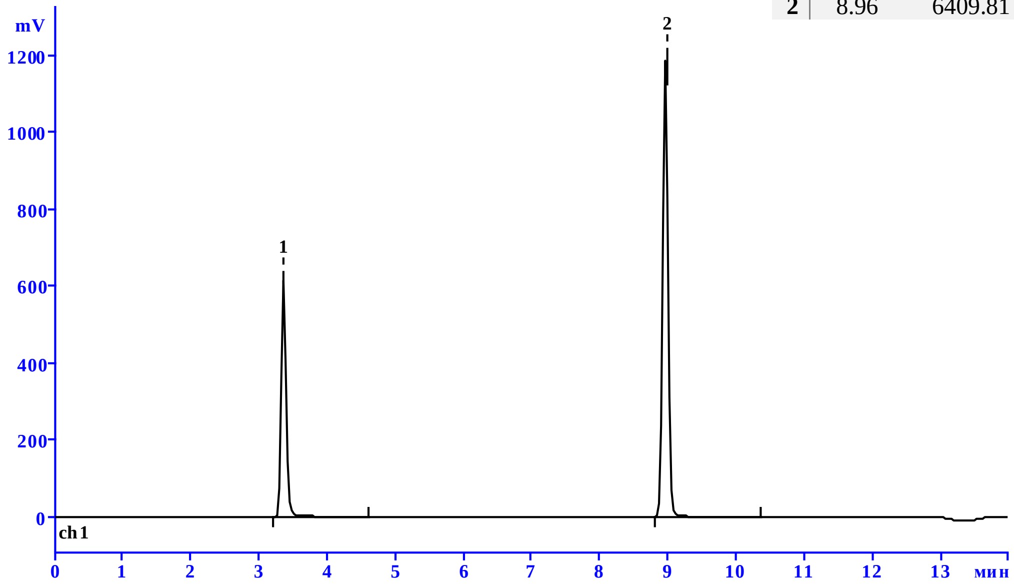

b) $t_{equilibrium}$

Thd+Ade+P_i – 3:1:1– $t_{equilibrium}$

№	RT, min	Area, mV*сек	Equilibrium concentration, µM	Name
**1**	3.27	202.63	12.95	Ade
**2**	5.63	2707.78	294.48	Thy
**3**	6.93	3223.17	187.05	dAdo
**4**	9.02	3263.87	305.52	Thd

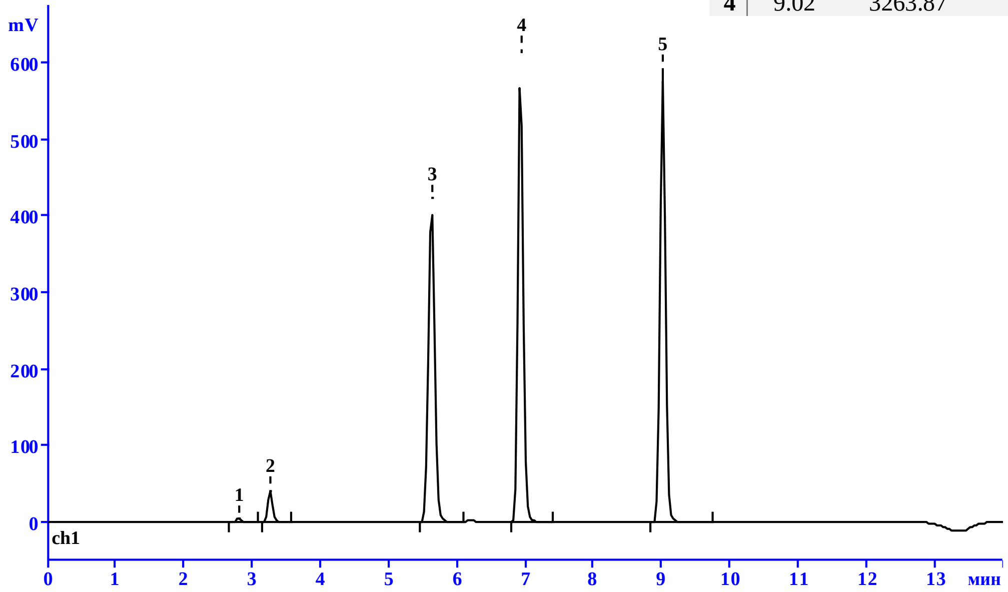

2′-deoxyuridine from thymidine (Thd+Ura+P_i, analytical method)

To a reaction sample solution (1 mL, Table S2) were added 1 U of TP *E. coli*. The reaction mixture was incubated at 37°C, pH 7.5, and was monitored by HPLC.

Table S2. Volumes of stock and buffer solutions taken for 1 mL of a rection mixture (pH 7.5).

2 mM Thd stock solution	2 mM Ura stock solution	5 mM phosphate	50 mM Tris - HCl	C_{Thd}:C_{Ura}:C_P
100 µL	150 µL	40 µL	710 µL	1:1.5:1
150 µL	100 µL	10 µL	740 µL	1.5:1:0.25
150 µL	100 µL	40 µL	710 µL	1.5:1:1
300 µL	100 µL	40 µL	560 µL	3:1:1

HPLC-analysis of transglycosylation: Cosmosil 5C18-MS-II, 4.6*150 mm, 5µm, 120 Å, (Nacalai Tesque, Inc. (Japan)), 20ºC, elution in modified mobile phase: linear gradient of acetonitrile in 0.06% TFA/deionized water from 2 to 12% for 10 min (flushing with 12-80 % acetonitrile-0.06% TFA/deionized water for 10-10.1 min, then 80-2% for 10.1-10.8 min) at flow rate 1 ml/min with UV detection at wavelength 260 nm, injection volume 20 µL.

Fugure S2.1. **1:1.5:1 ratio**

a) t_{initia}

Thd+Ura+P_i – 1:1.5:1 – t_0

№	RT, min	Area, mV*сек	Equilibrium concentration, µM	Name
**1**	2.80	2695.79	300.00	Ura
**2**	9.03	1947.44	200.00	Thd

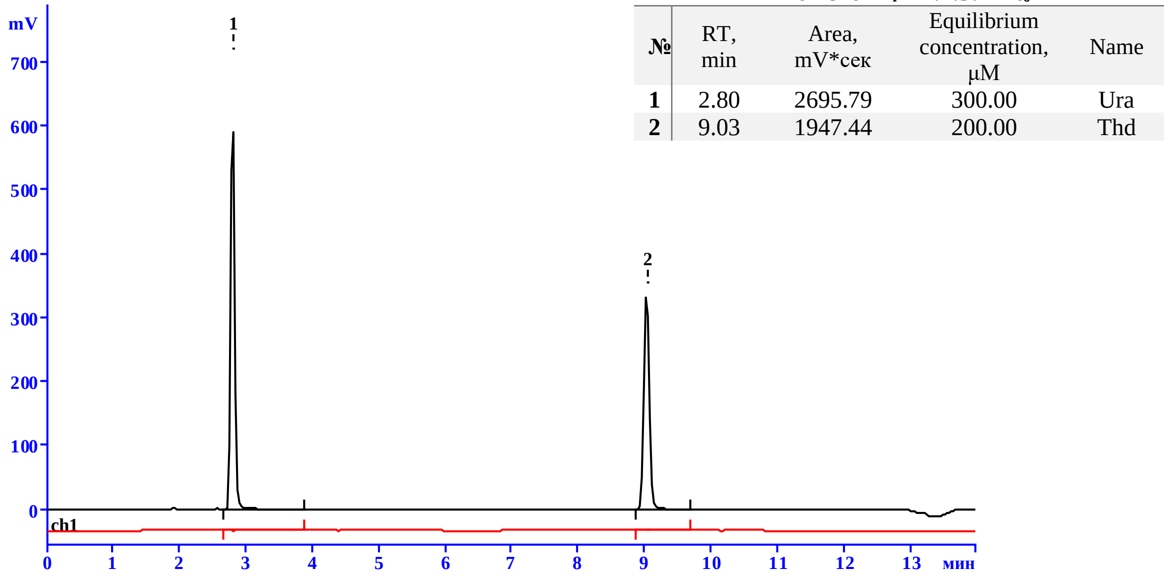

b) t_{equilibrium}

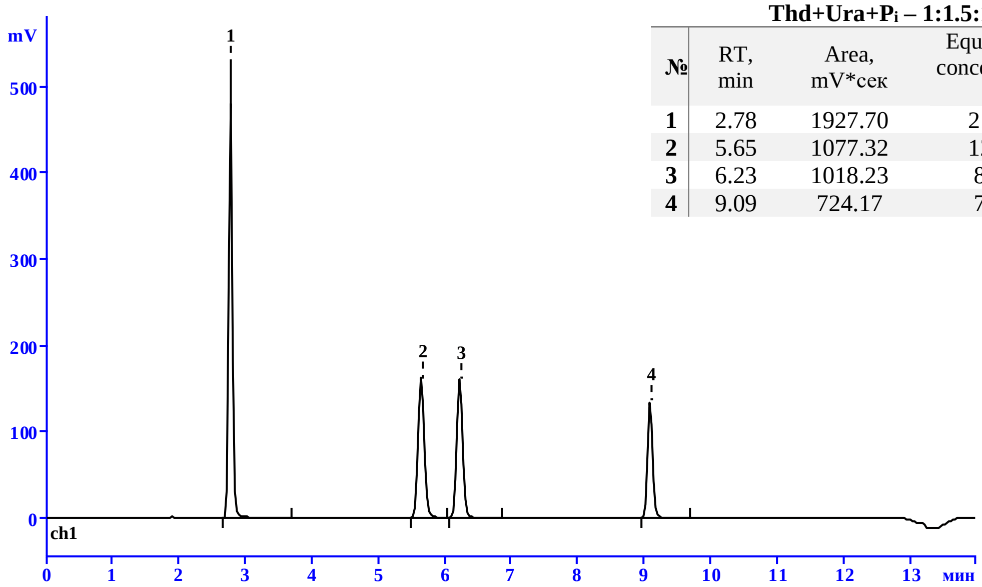

Thd+Ura+P_i – 1:1.5:1 – t $_{equilibrium}$

№	RT, min	Area, mV*сек	Equilibrium concentration, µM	Name
**1**	2.78	1927.70	214.52	Ura
**2**	5.65	1077.32	125.63	Thy
**3**	6.23	1018.23	85.48	dUrd
**4**	9.09	724.17	74.37	Thd

Fugure S2.2. **1.5:1:0.25 ratio**

a) $t_{initial}$

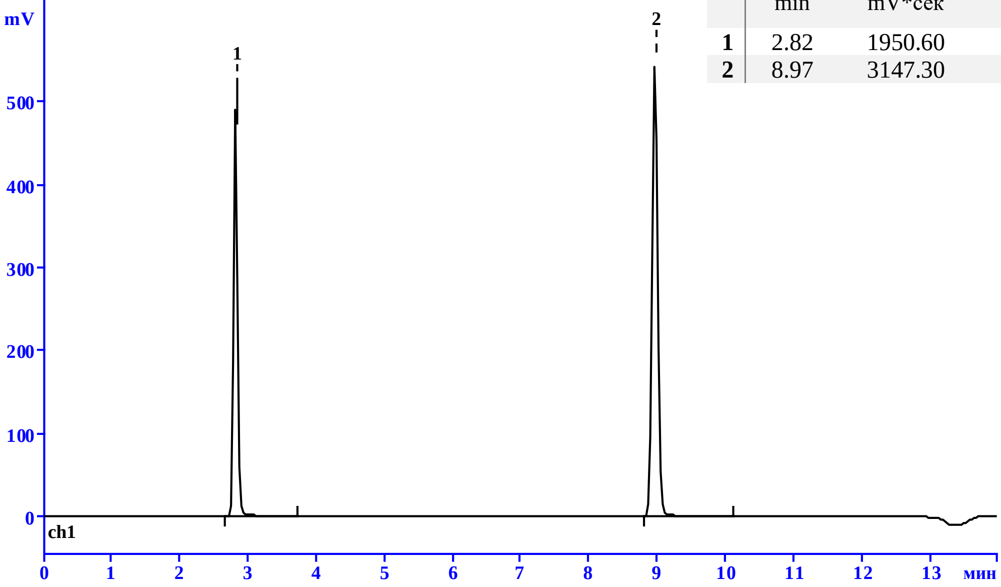

Thd+Ura+P_i – 1.5:1:0.25 – t_0

№	RT, min	Area, mV*сек	Equilibrium concentration, µM	Name
**1**	2.82	1950.60	200.00	Ura
**2**	8.97	3147.30	300.00	Thd

b) t$_{equilibrium}$

Thd+Ura+P$_i$ – 1.5:1:0.25 – t $_{equilibrium}$

№	RT, min	Area, mV*сек	Equilibrium concentration, µM	Name
**1**	2.79	1035.04	106.13	Ura
**2**	5.66	1227.45	156.37	Thy
**3**	6.26	995.79	93.87	dUrd
**4**	9.03	1506.87	143.63	Thd

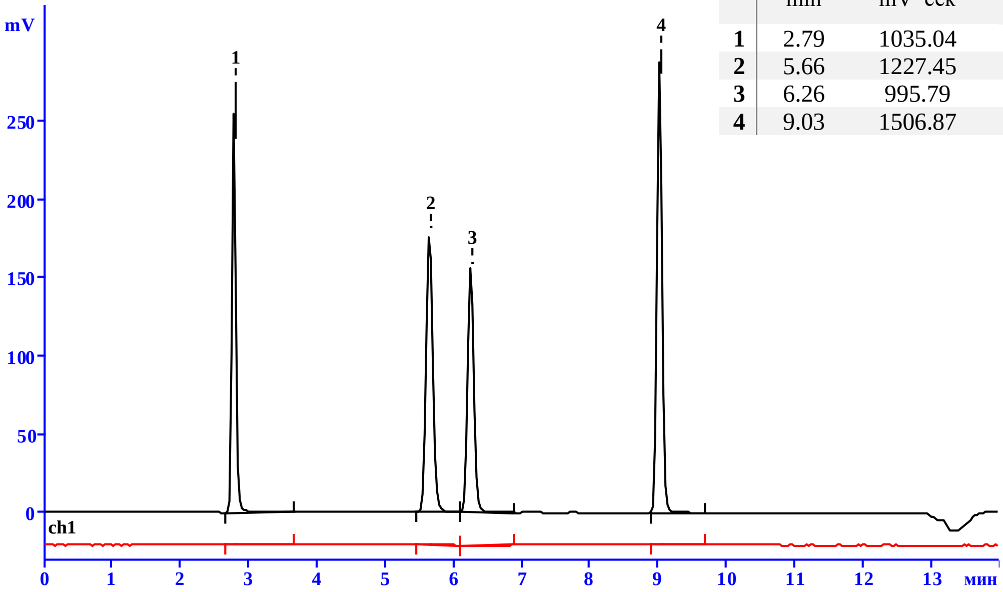

Fugure S2.3. **1.5:1:1 ratio**

a) t$_{initial}$

Thd+Ura+P$_i$ – 1.5:1:1 – t$_0$

№	RT, min	Area, mV*сек	Equilibrium concentration, µM	Name
**1**	2.80	1912.04	200.00	Ura
**2**	8.90	3129.59	300.00	Thd

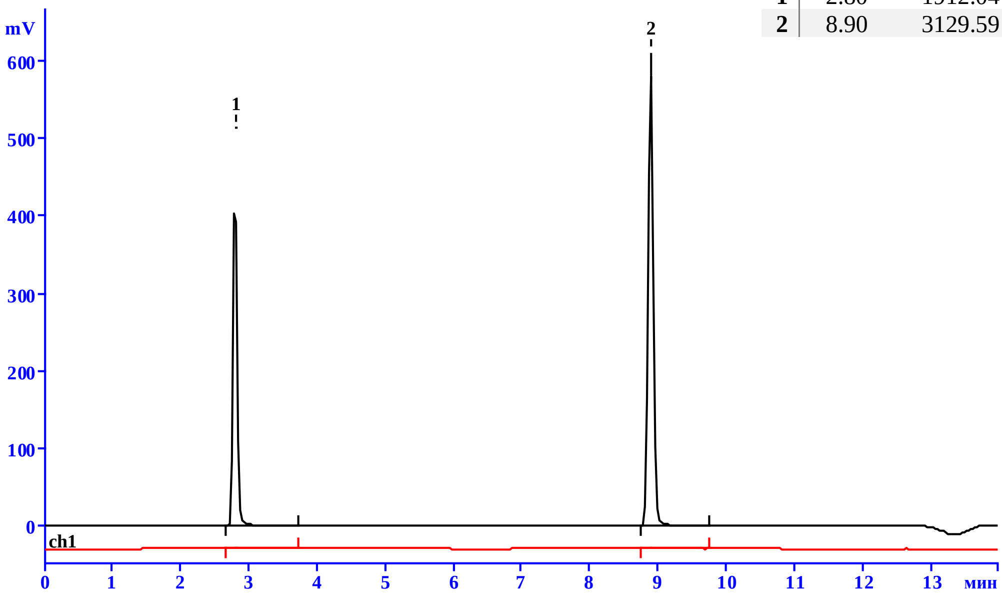

b) $t_{equilibrium}$

Thd+Ura+P_i – 1.5:1:1 – t $_{equilibrium}$

№	RT, min	Area, mV*сек	Equilibrium concentration, µM	Name
**1**	2.79	1091.23	114.14	Ura
**2**	5.67	1320.74	155.74	Thy
**3**	6.26	973.55	85.86	dUrd
**4**	9.11	1504.92	144.26	Thd

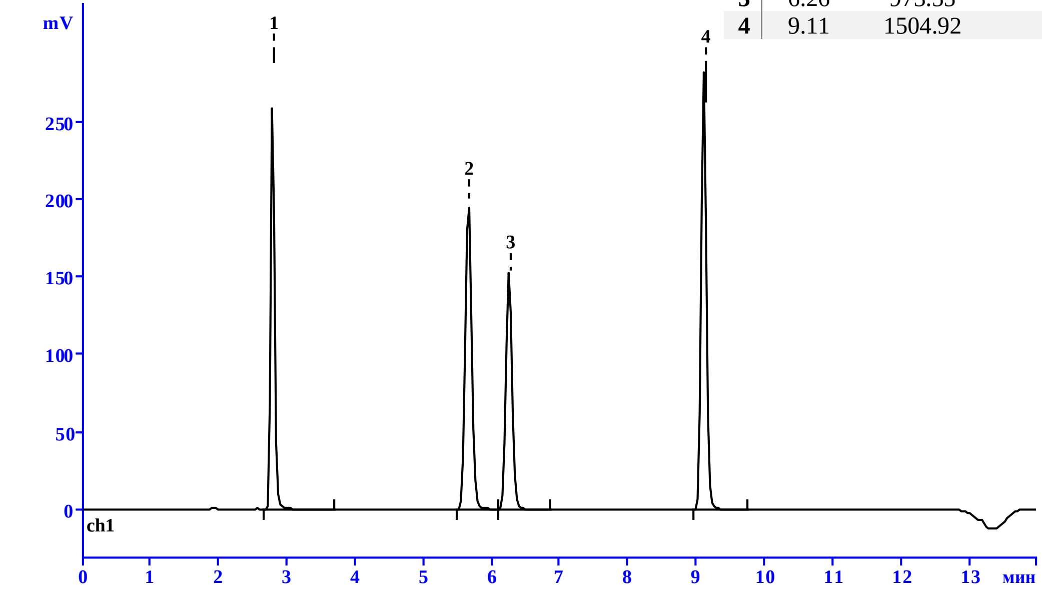

Fugure S2.4. **3:1:1 ratio**

a) $t_{initial}$

Thd+Ura+P_i – 3:1:1 – t_0

№	RT, min	Area, mV*сек	Equilibrium concentration, µM	Name
**1**	2.76	1899.05	200.00	Ura
**2**	8.94	6199.20	600.00	Thd

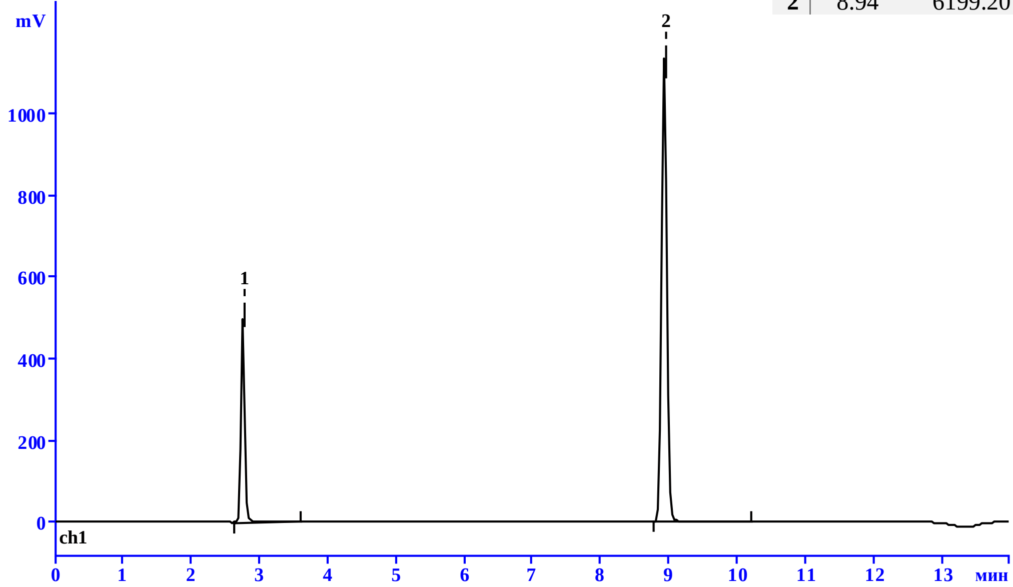

b) t$_{equilibrium}$

Thd+Ura+P$_i$ – 3:1:1 – t $_{equilibrium}$

№	RT, min	Area, mV*сек	Equilibrium concentration, µM	Name
**1**	2.80	823.41	86.72	Ura
**2**	5.66	1909.39	239.60	Thy
**3**	6.26	1250.72	113.28	dUrd
**4**	9.00	3723.68	360.40	Thd

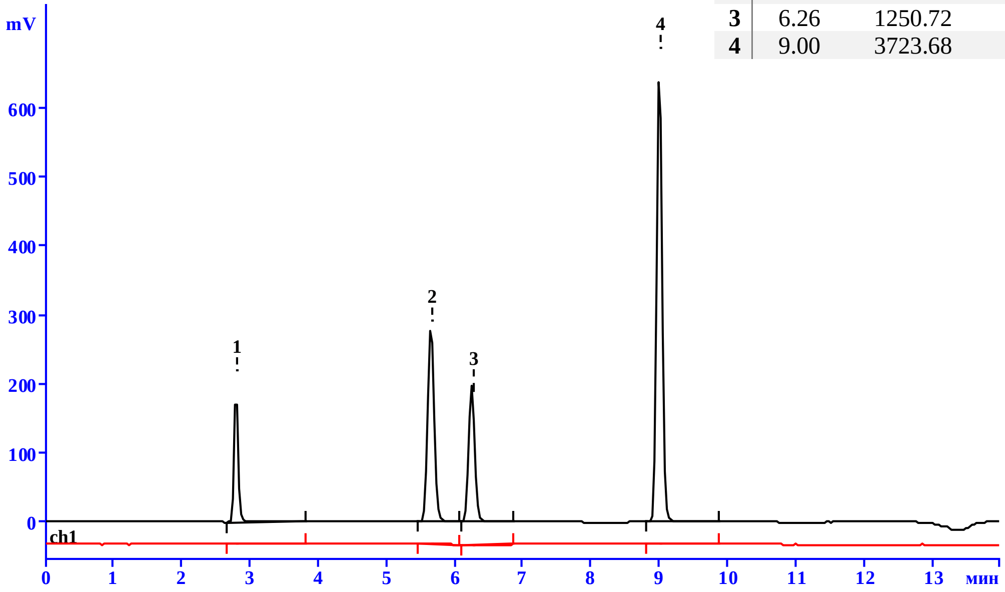

Thymidine from 2′-deoxyuridine (dUrd+5-Me-Ura+P$_i$, analytical method)

To a reaction sample solution (1 mL, Table S3) were added 1 U of TP *E. coli*. The reaction mixture was incubated at 37°C, pH 7.5, and was monitored by HPLC.

Table S3. Volumes of stock and buffer solutions taken for 1 mL of a rection mixture (pH 7.5).

2 mM dUrd stock solution	2 mM 5-Me-Ura stock solution	5 mM phosphate	50 mM Tris - HCl	C_{dUrd}:$C_{5\text{-MtUra}}$:C_P
100 µL	150 µL	40 µL	710 µL	1:1.5:1
150 µL	100 µL	10 µL	740 µL	1.5:1:0.25
150 µL	100 µL	40 µL	710 µL	1.5:1:1
300 µL	100 µL	40 µL	560 µL	3:1:1

HPLC-analysis of transglycosylation: Cosmosil 5C18-MS-II, 4.6*150 mm, 5µm, 120 Å, (Nacalai Tesque, Inc. (Japan)), 20°C, elution in modified mobile phase: linear gradient of acetonitrile in 0.06% TFA/deionized water from 2 to 12% for 10 min (flushing with 12-80 % acetonitrile-0.06% TFA/deionized water for 10-10.1 min, then 80-2% for 10.1-10.8 min) at flow rate 1 ml/min with UV detection at wavelength 260 nm, injection volume 20 µL.

Fugure S3.1. **1:1.5:1 ratio**

a) t initial

dUrd+5-MeUra+P_i – 1:1.5:1 – t_0

№	RT, min	Area, mV*сек	Equilibrium concentration, µM	Name
**1**	5.68	2584.98	300.00	5-MeUra
**2**	6.28	2315.16	200.00	Thd

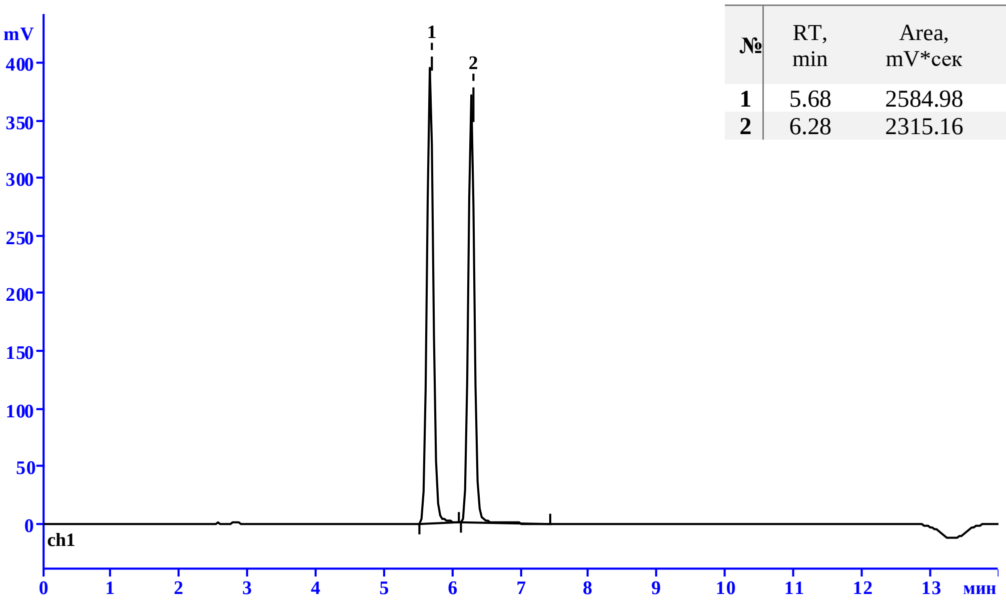

b) t equilibrium

dUrd+5-MeUra+P_i – 1:1.5:1 – t equilibrium

№	RT, min	Area, mV*сек	Equilibrium concentration, µM	Name
**1**	2.83	1376.99	142.96	Ura
**2**	5.70	1706.41	198.04	5-MeUra
**3**	6.28	660.23	57.04	dUrd
**4**	9.09	1026.84	101.96	Thd

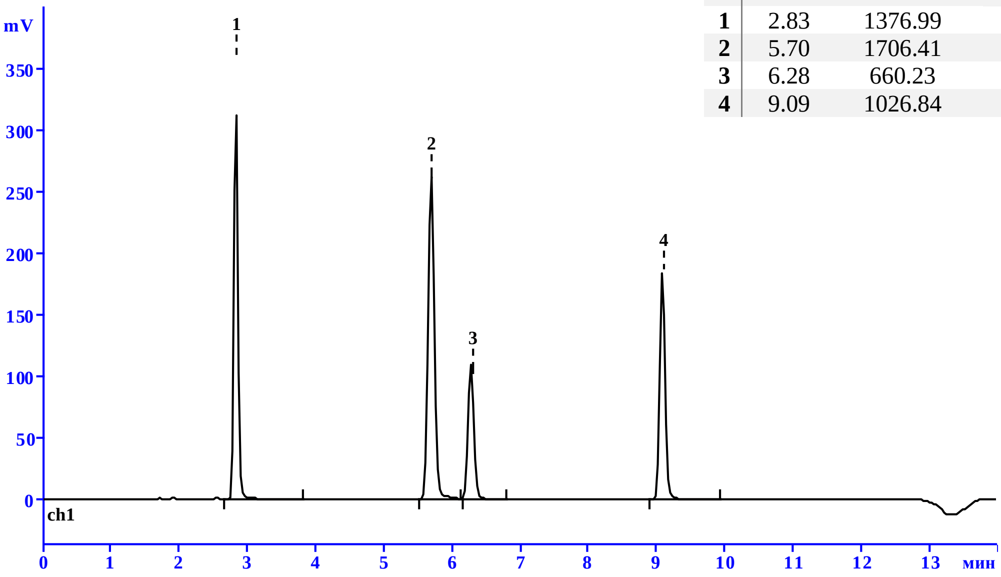

Fugure S3.2. **1.5:1:0.25 ratio**

a) $t_{initial}$

dURd+5-MeUra+P_i – 1.5:1:0.25 – t_0

№	RT, min	Area, mV*сек	Equilibrium concentration, µM	Name
**1**	5.49	1694.52	200.00	5-MeUra
**2**	6.04	3360.98	300.00	Thd

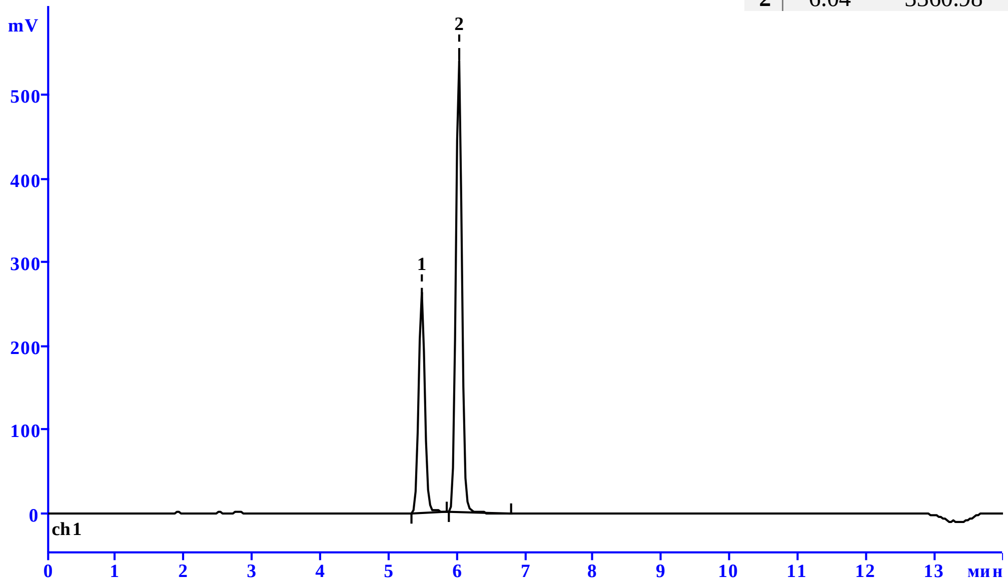

b) $t_{equilibrium}$

dUrd+5-MeUra+P_i – 1.5:1:0.25 – t $_{equilibrium}$

№	RT, min	Area, mV*сек	Equilibrium concentration, µM	Name
**1**	2.85	1592.34	158.59	Ura
**2**	5.68	820.75	96.87	5-MeUra
**3**	6.28	1584.24	141.41	dUrd
**4**	9.00	1044.35	103.13	Thd

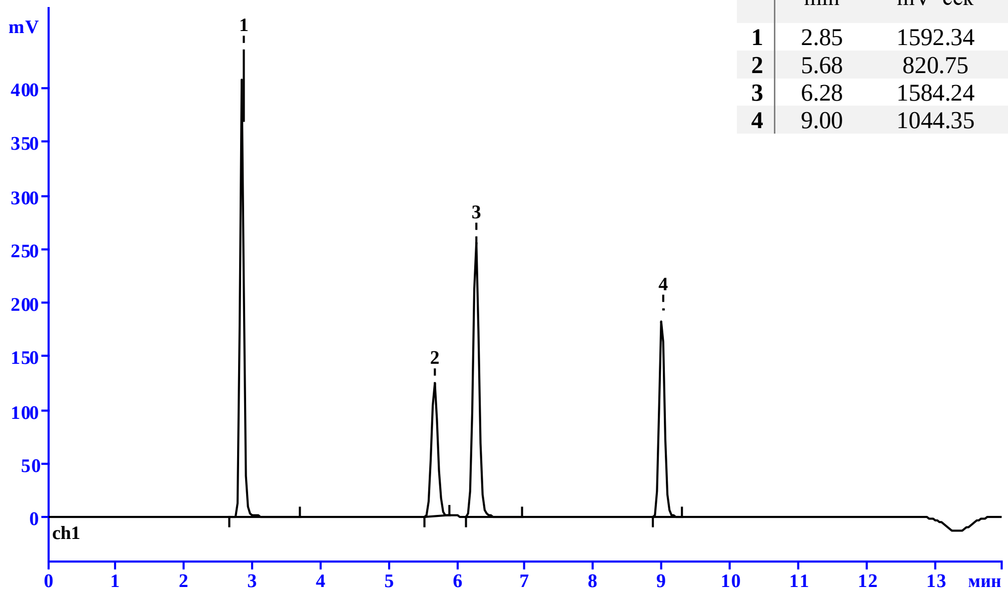

Fugure S3.3. **1.5:1:1 ratio**

a) t_{initial}

dUrd+5-MeUra+P_i – 1.5:1:1 – t_0

№	RT, min	Area, mV*сек	Equilibrium concentration, µM	Name
**1**	5.56	1793.88	200.00	5-MeUra
**2**	6.16	3508.87	300.00	Thd

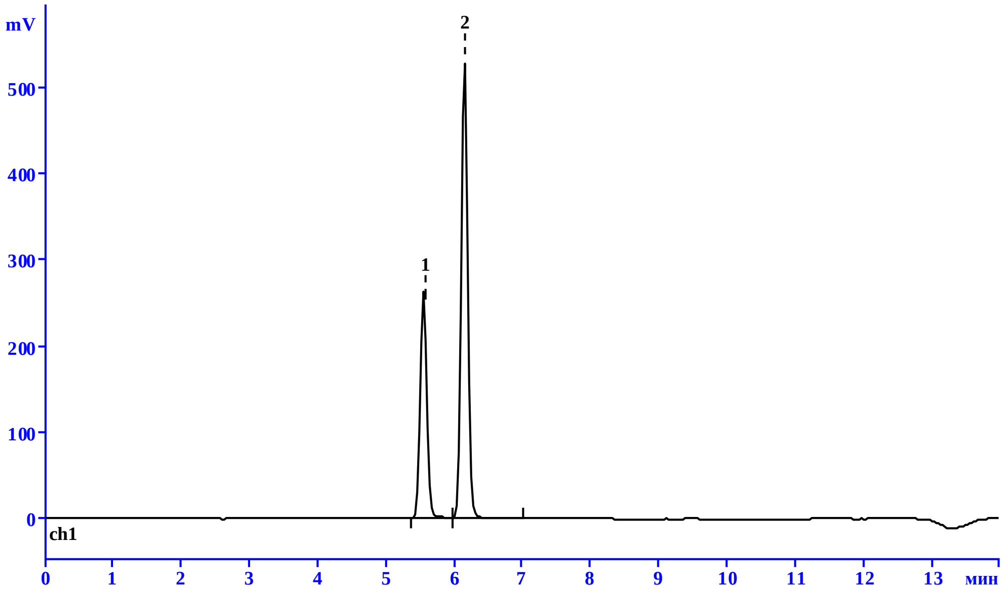

b) t_{equilibrium}

dUrd+5-MeUra+P_i – 1.5:1:1 – t equilibrium

№	RT, min	Area, mV*сек	Equilibrium concentration, µM	Name
**1**	2.84	1603.82	170.69	Ura
**2**	5.74	842.79	93.96	5-MeUra
**3**	6.32	1512.42	129.31	dUrd
**4**	9.13	1016.56	106.04	Thd

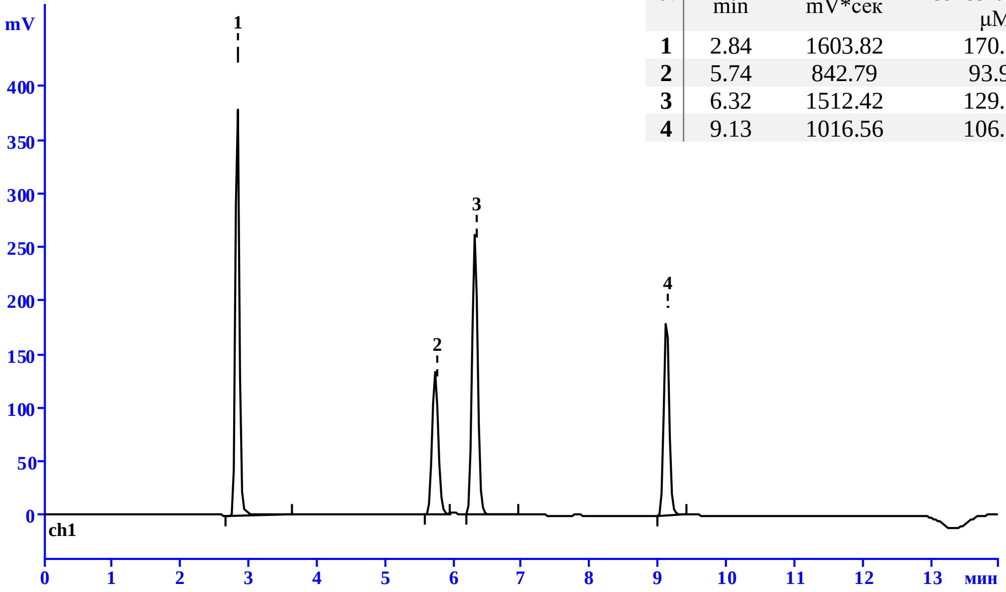

Fugure S3.4. **3:1:1 ratio**

a) $t_{initial}$

dUrd+5-MeUra+P_i – 3:1:1 – t_0

№	RT, min	Area, mV*сек	Equilibrium concentration, µM	Name
**1**	5.58	1710.68	200.00	5-MeUra
**2**	6.16	6831.56	600.00	Thd

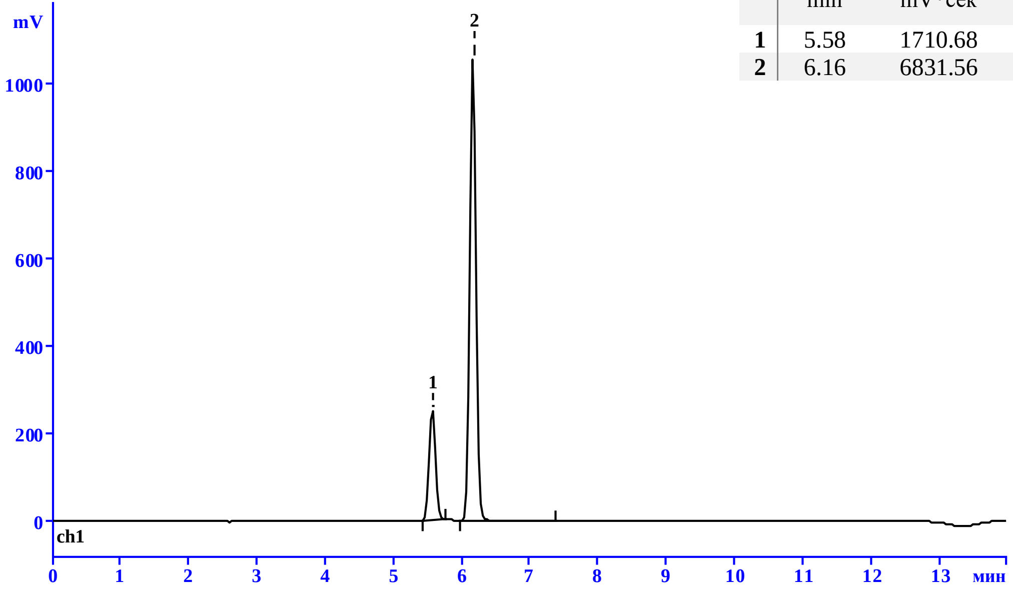

b) $t_{equilibrium}$

dUrd+5-MeUra+P_i – 3:1:1 – t equilibrium

№	RT, min	Area, mV*сек	Equilibrium concentration, µM	Name
**1**	2.81	2578.04	264.45	Ura
**2**	5.71	653.43	76.39	5-MeUra
**3**	6.36	3820.53	335.55	dUrd
**4**	9.21	1249.20	123.61	Thd

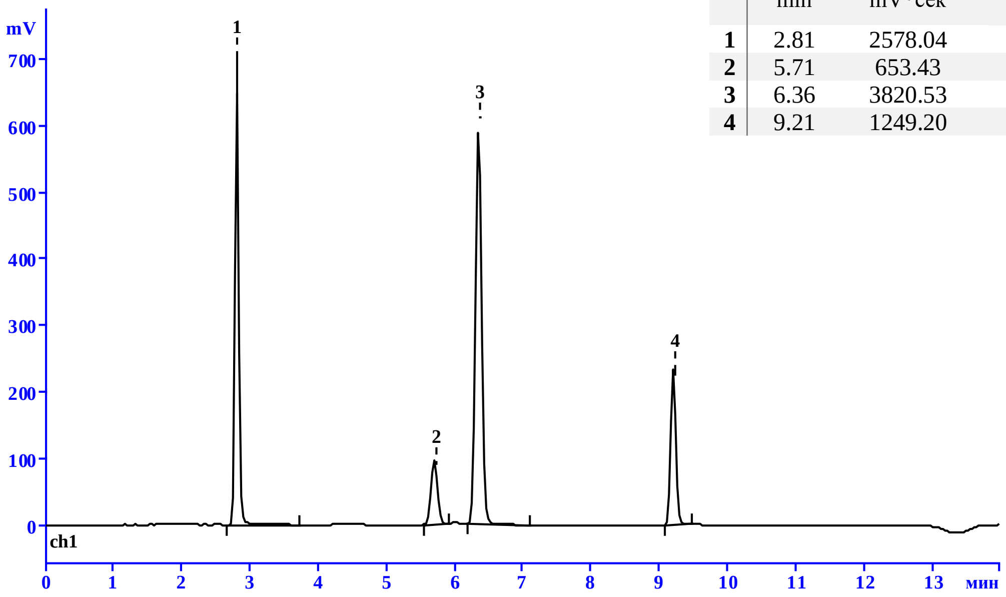

2′-deoxyuridine from 2′-deoxyadenosine (dAdo+Ura+P_i, analytical method)

To a reaction sample solution (1 mL, Table S4) were added 1 U of PNP *E. coli* and 1 U of TP *E. coli*. The reaction mixture was incubated at 37ºC, pH 7.5, and was monitored by HPLC.

Table S4. Volumes of stock and buffer solutions taken for 1 mL of a rection mixture (pH 7.5).

1 mM dAdo stock solution	2 mM Ura stock solution	5 mM phosphate	50 mM Tris - HCl	C_{dAdo}:C_{Ura}:C_P
200 µL	150 µL	40 µL	610 µL	1:1.5:1
300 µL	100 µL	10 µL	590 µL	1.5:1:0.25
300 µL	100 µL	40 µL	560 µL	1.5:1:1
600 µL	100 µL	40 µL	260 µL	3:1:1

HPLC-analysis of transglycosylation: Cosmosil 5C18-MS-II, 4.6*150 mm, 5µm, 120 Å, (Nacalai Tesque, Inc. (Japan)), 20ºC, elution in modified mobile phase: linear gradient of acetonitrile in 0.06% TFA/deionized water from 2 to 12% for 10 min (flushing with 12-80 % acetonitrile-0.06% TFA/deionized water for 10-10.1 min, then 80-2% for 10.1-10.8 min) at flow rate 1 ml/min with UV detection at wavelength 260 nm, injection volume 20 µL.

Fugure S4.1. **1:1.5:1 ratio**

a) $t_{initial}$

dAdo+Ura+P_i – 1:1.5:1 – t_0

№	RT, min	Area, mV*сек	Equilibrium concentration, µM	Name
**1**	2.85	2859.90	300.00	Ura
**2**	7.06	3448.62	200.00	dAdo

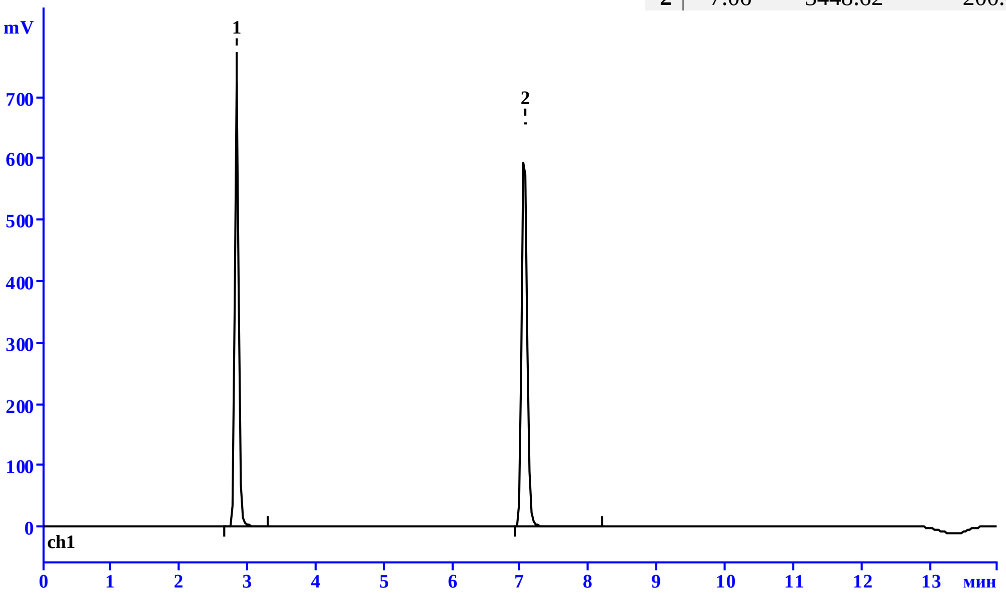

b) $t_{equilibrium}$

dAdo+Ura+P_i – 1:1.5:1 – t $_{equilibrium}$

№	RT, min	Area, mV*сек	Equilibrium concentration, µM	Name
**1**	2.77	2520.97	264.45	Ura
**2**	3.22	871.92	55.00	Ade
**3**	6.24	454.39	35.55	dUrd
**4**	7.04	2500.18	145.00	dAdo

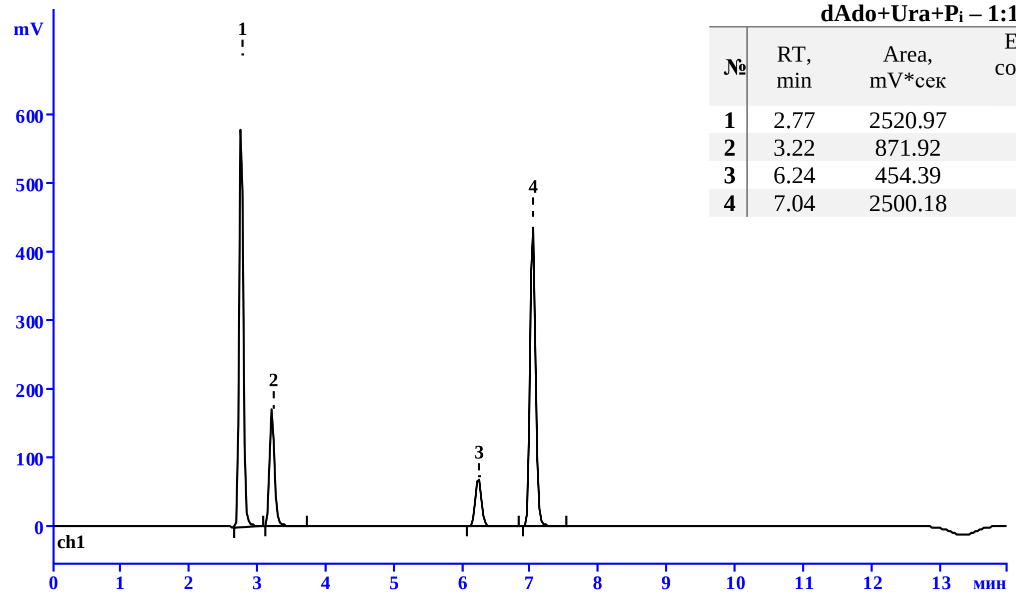

Fugure S4.2. **1.5:1:0.25 ratio**

a) $t_{initial}$

dAdo+Ura+P_i – 1.5:1:0.25 – t_0

№	RT, min	Area, mV*сек	Equilibrium concentration, µM	Name
**1**	2.79	1946.28	200.00	Ura
**2**	6.97	5205.42	300.00	dAdo

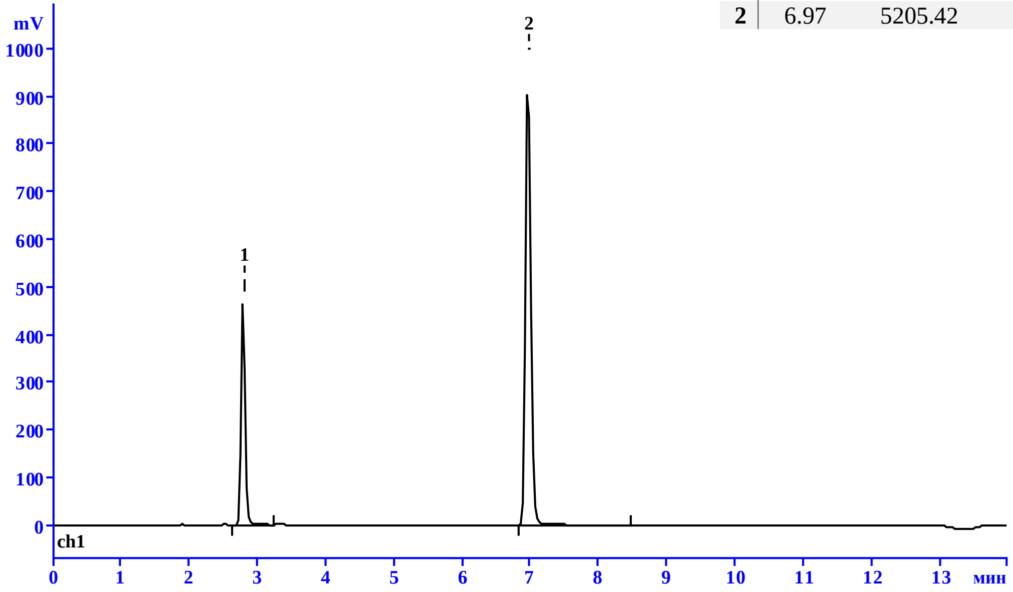

b) $t_{equilibrium}$

dAdo+Ura+P_i – 1.5:1:0.25 – t $_{equilibrium}$

№	RT, min	Area, mV*сек	Equilibrium concentration, µM	Name
**1**	2.83	1552.09	159.49	Ura
**2**	3.32	877.75	60.32	Ade
**3**	6.35	470.90	40.51	dUrd
**4**	7.05	4158.74	239.68	dAdo

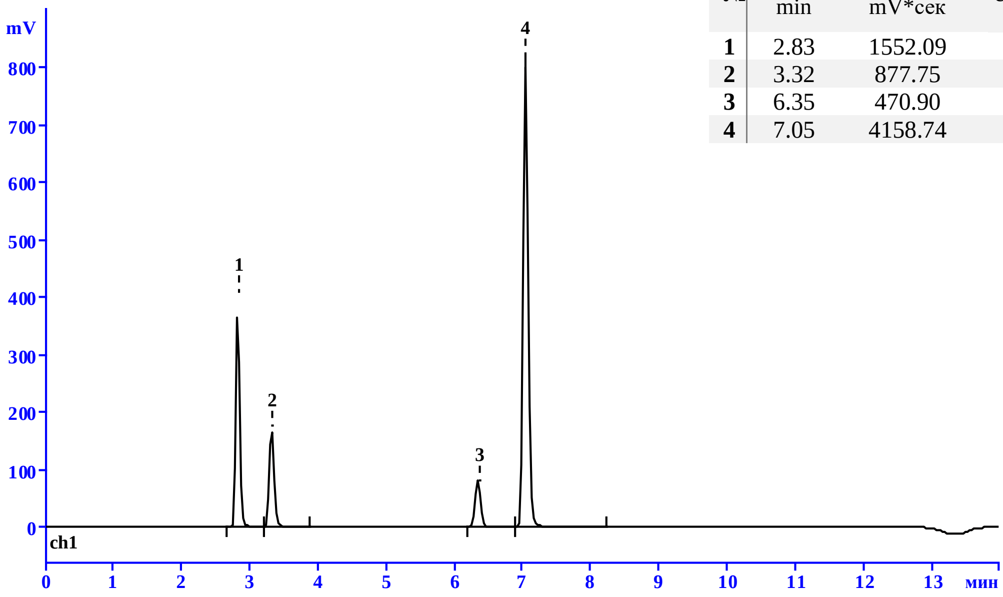

Fugure S4.4. **1.5:1:1 ratio**

a) $t_{initial}$

dAdo+Ura+P_i – 1.5:1:1 – t_0

№	RT, min	Area, mV*сек	Equilibrium concentration, µM	Name
**1**	2.79	1920.66	200.00	Ura
**2**	6.98	5239.84	300.00	dAdo

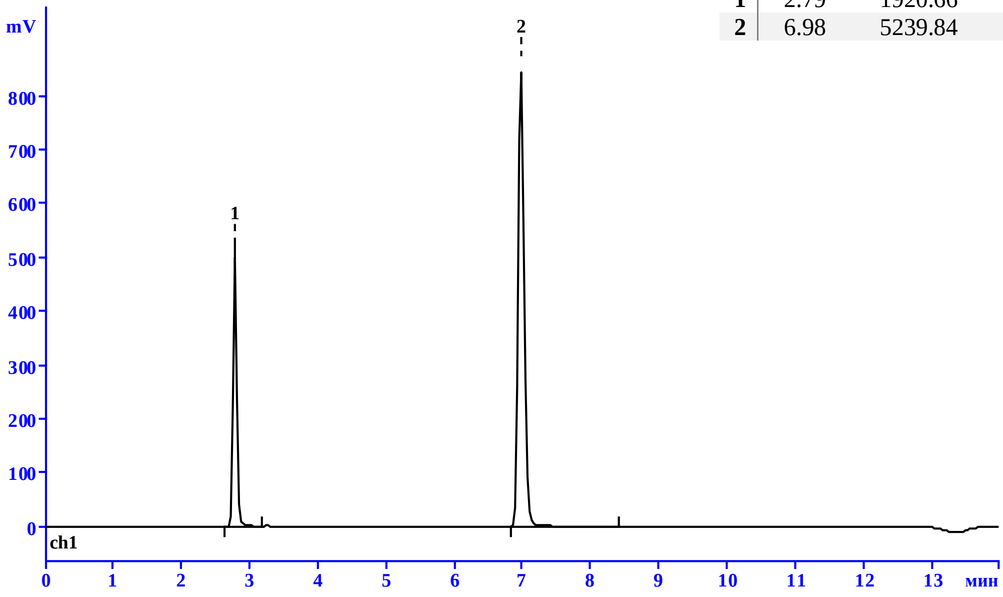

b) $t_{equilibrium}$

dAdo+Ura+P_i – 1.5:1:1 – t $_{equilibrium}$

№	RT, min	Area, mV*сек	Equilibrium concentration, µM	Name
**1**	2.84	1579.50	164.47	Ura
**2**	3.34	949.15	66.75	Ade
**3**	6.28	430.49	35.53	dUrd
**4**	7.04	4073.99	233.25	dAdo

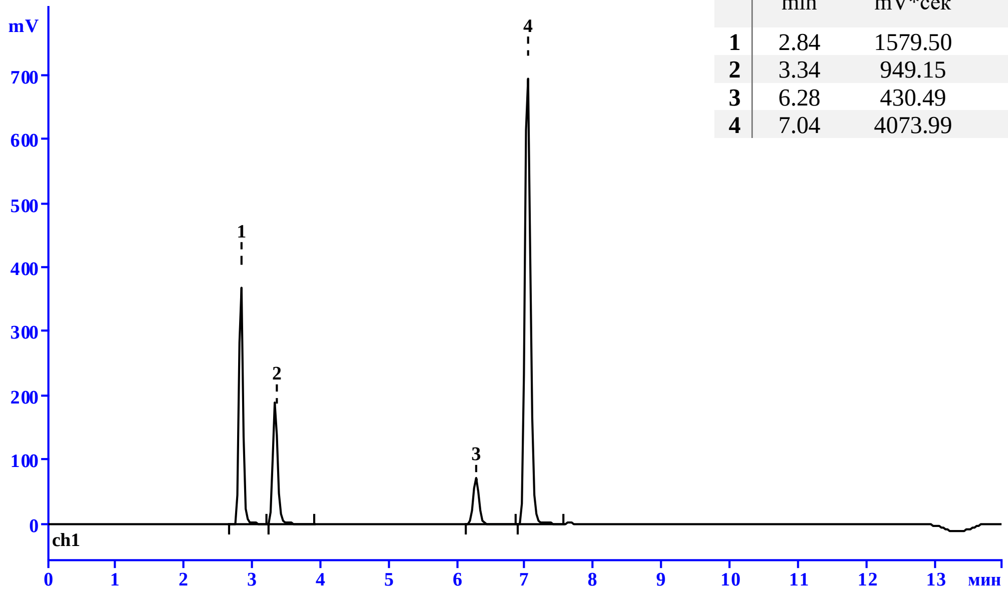

Fugure S4.4. **3:1:1 ratio**

a) $t_{initial}$

dAdo+Ura+P_i – 3:1:1 – t_0

№	RT, min	Area, mV*сек	Equilibrium concentration, µM	Name
**1**	2.80	1914.04	200.00	Ura
**2**	6.92	10223.76	600.00	dAdo

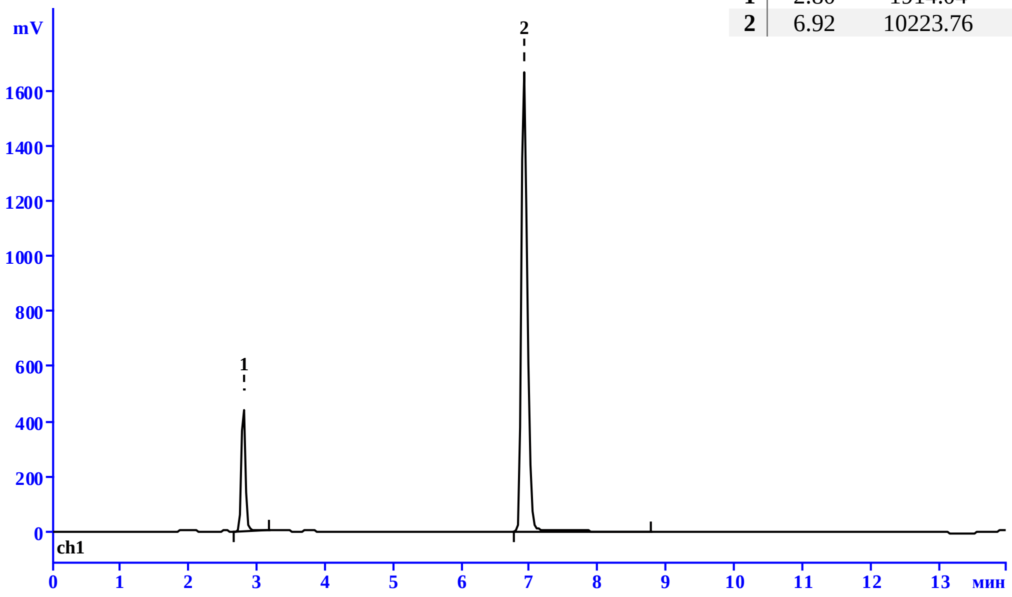

b) $t_{equilibrium}$

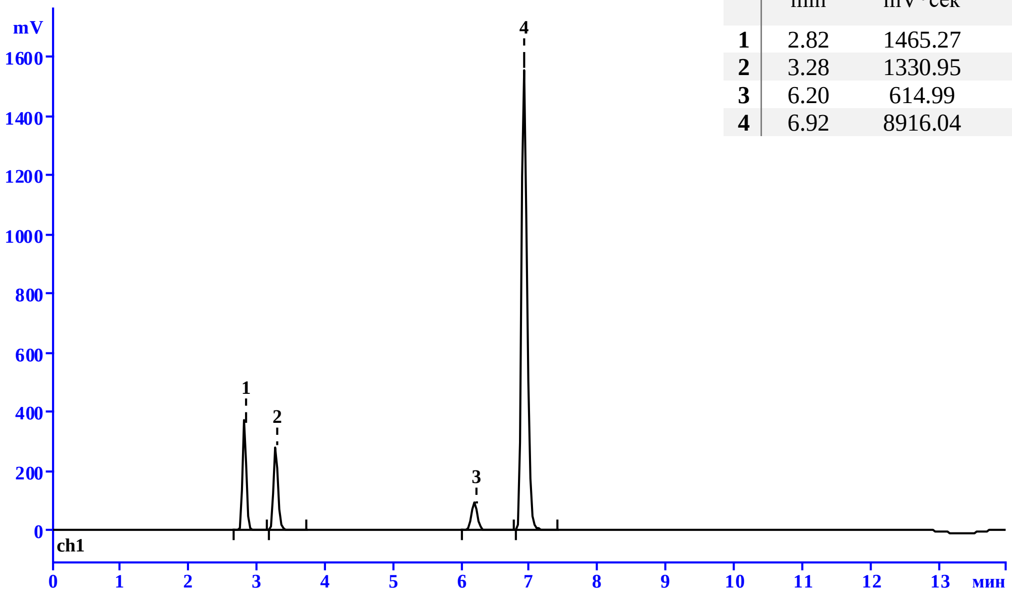

dAdo+Ura+P_i – 3:1:1 – t equilibrium

№	RT, min	Area, mV*сек	Equilibrium concentration, µM	Name
**1**	2.82	1465.27	153.11	Ura
**2**	3.28	1330.95	76.75	Ade
**3**	6.20	614.99	46.89	dUrd
**4**	6.92	8916.04	523.25	dAdo

2′-deoxyinosine from 2′-deoxyadenosine (dAdo+Hyp+P_i, analytical method)

To a reaction sample solution (1 mL, Table S5) were added 1 U of PNP *E. coli*. The reaction mixture was incubated at 37°C, pH 7.5, and was monitored by HPLC.

Table S5. Volumes of stock and buffer solutions taken for 1 mL of a rection mixture (pH 7.5).

1 mM dAdo stock solution	2 mM Hyp stock solution	5 mM phosphate	50 mM Tris - HCl	C_{dAdo}:C_{Hyp}:C_P
200 µL	150 µL	40 µL	610 µL	1:1.5:1
300 µL	100 µL	10 µL	590 µL	1.5:1:0.25
300 µL	100 µL	40 µL	560 µL	1.5:1:1
600 µL	100 µL	40 µL	260 µL	3:1:1

HPLC-analysis of transglycosylation: Cosmosil 5CN-MS, 4.6*250 mm, 5µm, 300 Å, (Nacalai Tesque, Inc. (Japan)), 20°C, elution in modified mobile phase: linear gradient of acetonitrile in 10 mM NaOAc/deionized water from 2 to 12% for 10 min (flushing with 12-80 % acetonitrile-10 mM NaOAc/deionized water for 10-10.1 min, then 80-2% for 10.1-10.8 min) at flow rate 1 ml/min with UV detection at wavelength 260 nm, injection volume 20 µL.

Fugure S5.1. **1:1.5:1 ratio.**

a) $t_{initial}$

dAdo+Hyp+P_i – 1:1.5:1 – t_0

№	RT, min	Area, mV*сек	Equilibrium concentration, µM	Name
**1**	4.27	2988.88	300.00	Hyp
**2**	7.94	3274.05	200.00	dAdo

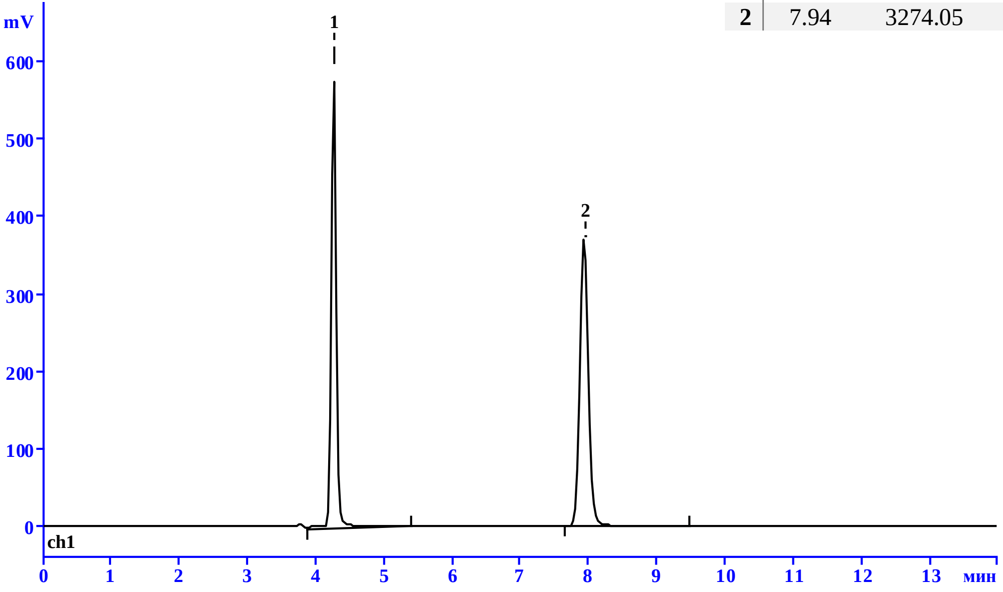

b) $t_{equilibrium}$

dAdo+Hyp+P_i – 1:1.5:1 – $t_{equilibrium}$

№	RT, min	Area, mV*сек	Equilibrium concentration, µM	Name
**1**	4.27	1979.45	198.68	Hyp
**2**	4.72	722.98	101.32	dIno
**3**	4.99	56.30	-	-
**4**	5.89	1415.56	99.36	Ade
**5**	7.89	1647.48	101.32	dAdo

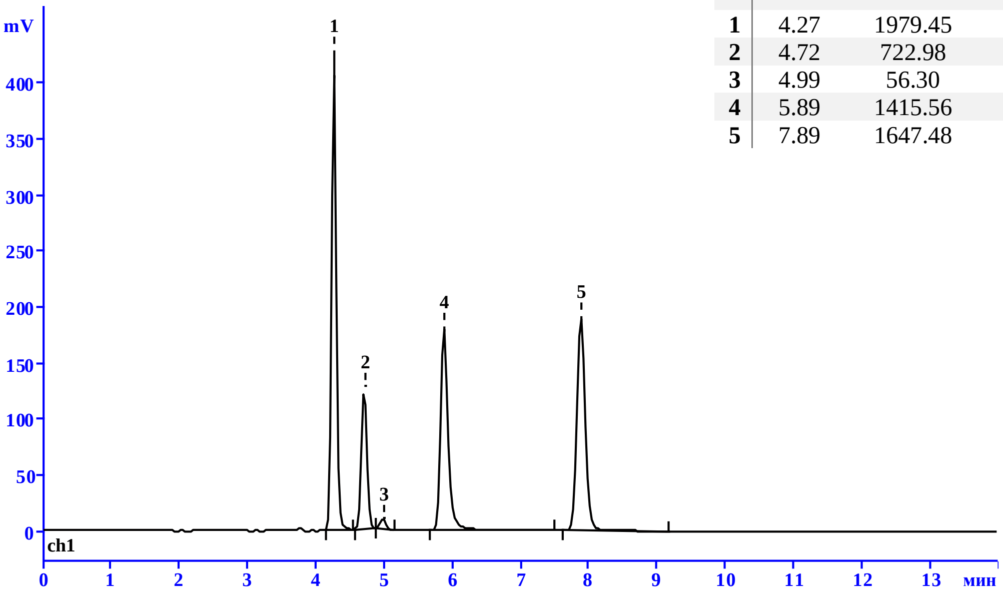

Fugure S5.2. **1.5:1:0.25 ratio.**

dAdo+Hyp+P_i – 1.5:1:0.25 – t_0

№	RT, min	Area, mV*сек	Equilibrium concentration, µM	Name
**1**	4.28	2071.76	200.00	Hyp
**2**	7.92	4904.99	300.00	dAdo

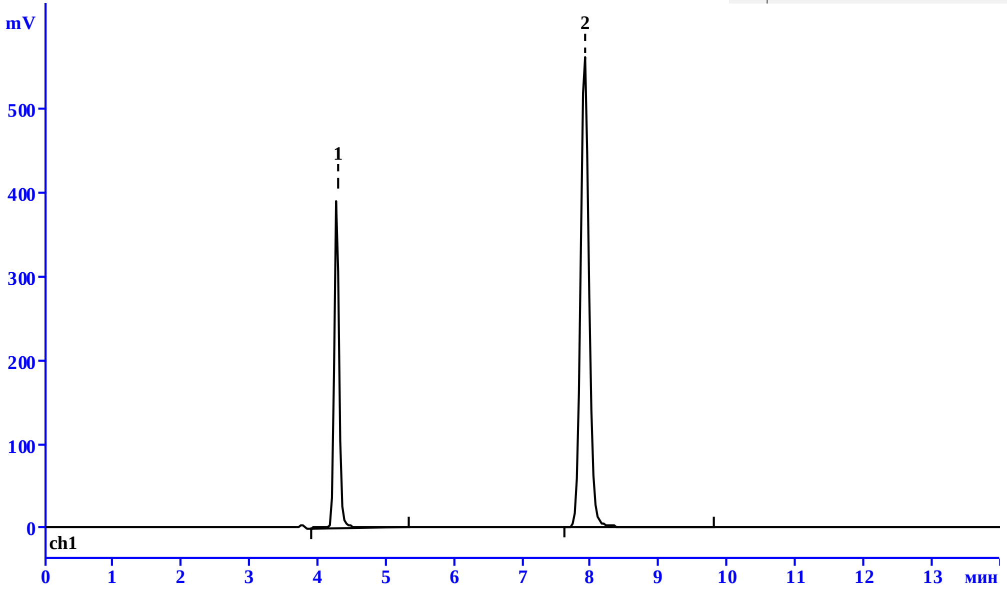

b) $t_{equilibrium}$

dAdo+Hyp+P_i – 1.5:1:0.25– t equilibrium

№	RT, min	Area, mV*сек	Equilibrium concentration, µM	Name
**1**	4.29	1038.61	100.26	Hyp
**2**	4.74	680.62	99.74	dIno
**3**	5.01	133.12	-	-
**4**	5.92	1505.79	105.25	Ade
**5**	7.94	3184.13	194.75	dAdo

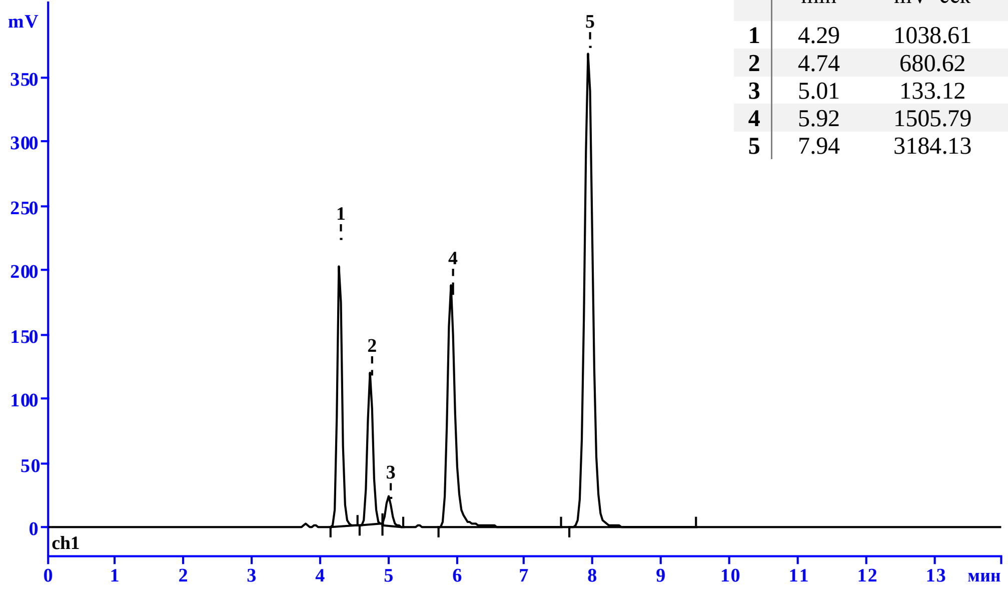

Fugure S5.3. **1.5:1:1 ratio.**

dAdo+Hyp+P_i – 1.5:1:1 – t_0

№	RT, min	Area, mV*сек	Equilibrium concentration, µM	Name
**1**	4.29	1927.15	200.00	Hyp
**2**	7.93	5039.74	300.00	dAdo

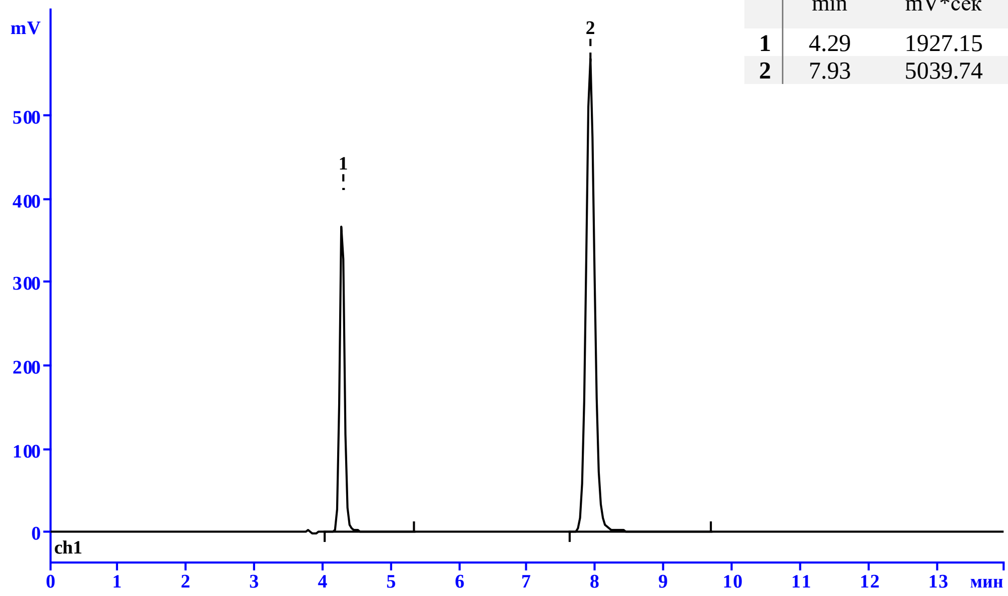

b) $t_{equilibrium}$

dAdo+Hyp+P_i – 1.5:1:1– $t_{equilibrium}$

№	RT, min	Area, mV*сек	Equilibrium concentration, µM	Name
**1**	4.29	1062.78	110.30	Hyp
**2**	4.75	775.00	89.70	dIno
**3**	5.95	1467.01	99.77	Ade
**4**	8.04	3363.63	200.23	dAdo

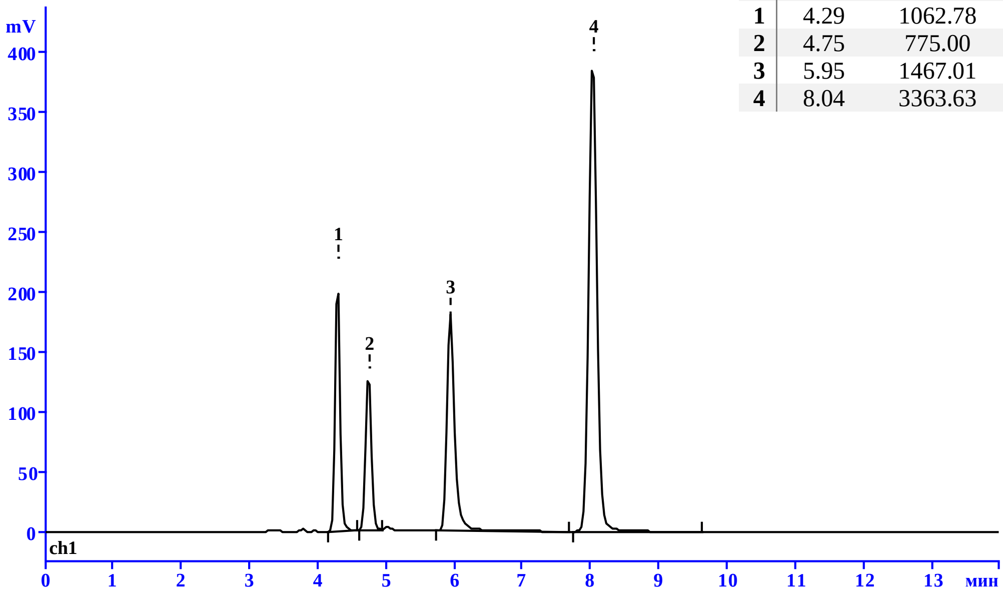

Fugure S5.4. **3:1:1 ratio.**

dAdo+Hyp+P_i – 3:1:1 – t_0

№	RT, min	Area, mV*сек	Equilibrium concentration, µM	Name
**1**	4.28	1945.10	200.00	Hyp
**2**	7.95	9832.28	600.00	dAdo

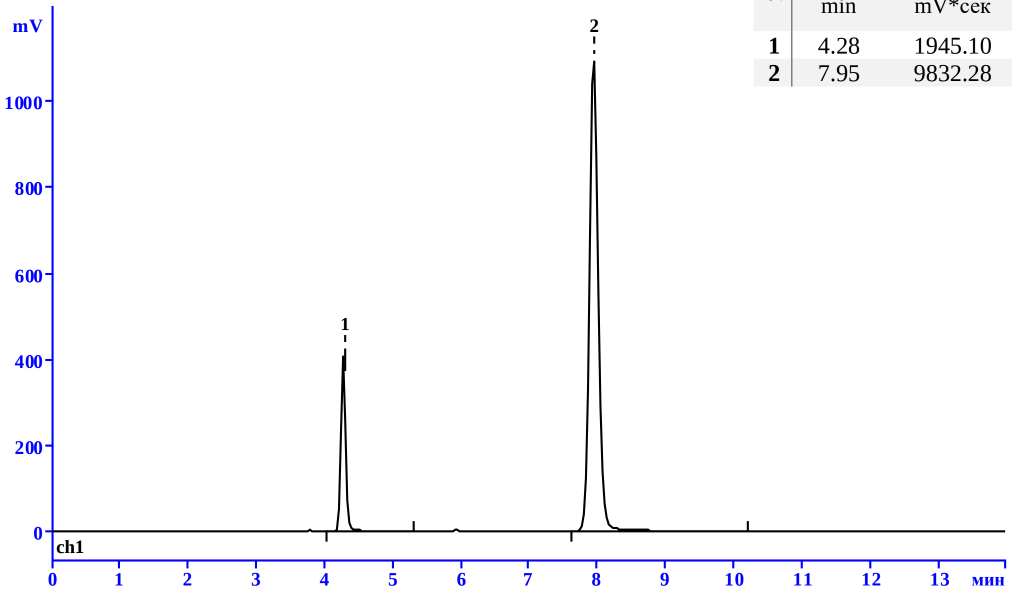

b) $t_{equilibrium}$

dAdo+Hyp+P_i – 3:1:1– t $_{equilibrium}$

№	RT, min	Area, mV*сек	Equilibrium concentration, µM	Name
**1**	4.28	778.69	80.07	Hyp
**2**	4.73	929.23	119.93	dIno
**3**	5.01	105.09	-	-
**4**	5.92	1957.03	138.59	Ade
**5**	7.93	7561.23	461.41	dAdo

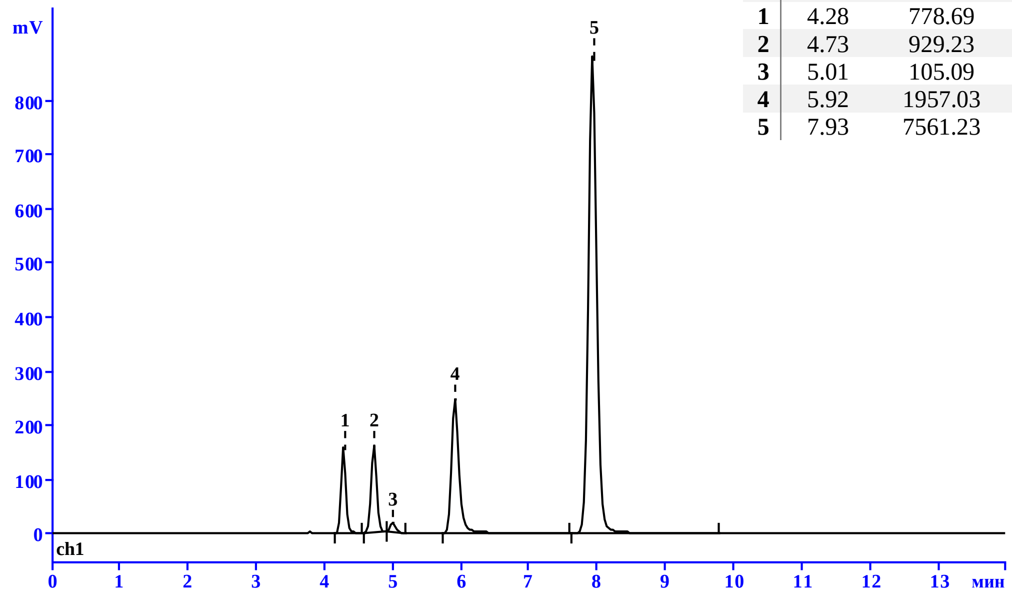

2′-deoxyadenosine from 2′-deoxyinosine (dIno+Ade+P_i, analytical method)

To a reaction sample solution (1 mL, Table S6) were added 1 U of PNP *E. coli*. The reaction mixture was incubated at 37°C, pH 7.5, and was monitored by HPLC.

Table S6. Volumes of stock and buffer solutions taken for 1 mL of a rection mixture (pH 7.5).

1 mM dIno stock solution	2 mM Ade stock solution	5 mM phosphate	50 mM Tris - HCl	C_{dIno}:C_{Ade}:C_P
200 µL	150 µL	40 µL	610 µL	1:1.5:1
300 µL	100 µL	10 µL	590 µL	1.5:1:0.25
300 µL	100 µL	40 µL	560 µL	1.5:1:1
600 µL	100 µL	40 µL	260 µL	3:1:1

HPLC-analysis of transglycosylation: Cosmosil 5CN-MS, 4.6*250 mm, 5µm, 300 Å, (Nacalai Tesque, Inc. (Japan)), 20°C, elution in modified mobile phase: linear gradient of acetonitrile in 10 mM NaOAc/deionized water from 2 to 12% for 10 min (flushing with 12-80 % acetonitrile-10 mM NaOAc/deionized water for 10-10.1 min, then 80-2% for 10.1-10.8 min) at flow rate 1 ml/min with UV detection at wavelength 260 nm, injection volume 20 µL.

Fugure S6.1. **1:1.5:1 ratio**

a) $t_{initial}$

dIno+Ade+P_i – 1:1.5:1 – t_0

№	RT, min	Area, mV*сек	Equilibrium concentration, µM	Name
**1**	4.65	1780.78	200.00	dIno
**2**	5.81	4643.55	300.00	Ade

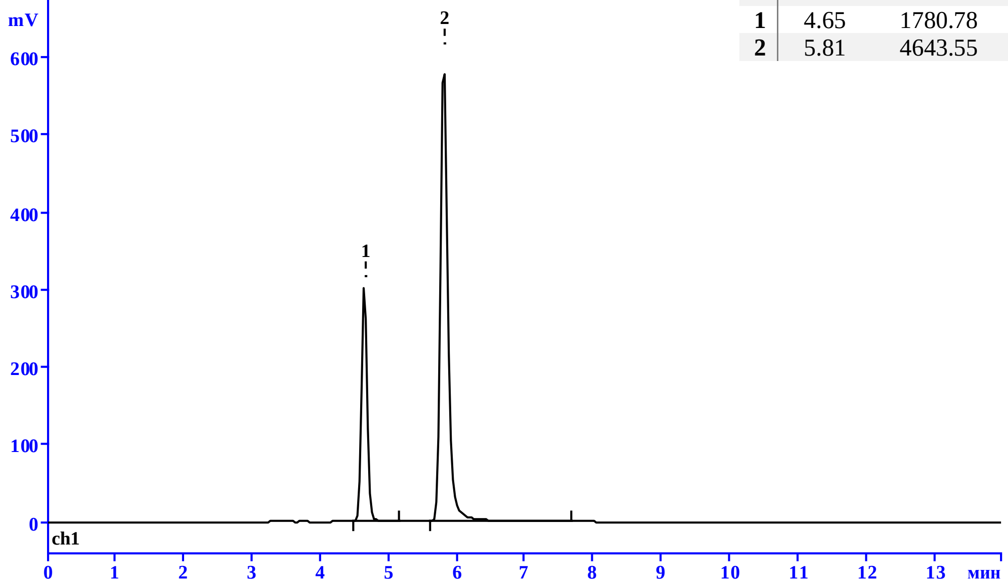

b) $t_{equilibrium}$

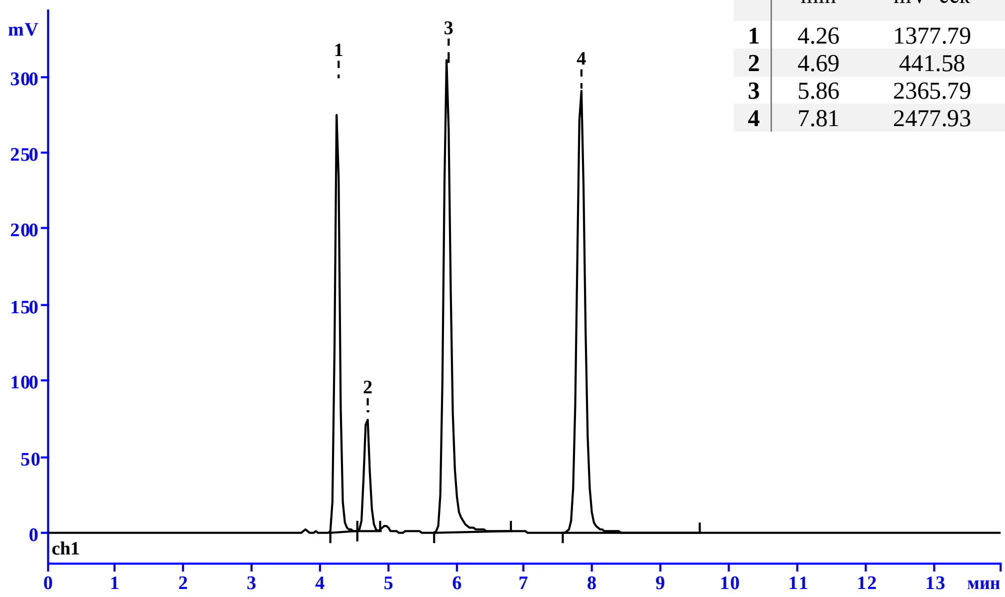

dIno+Ade+P_i – 1:1.5:1– t $_{equilibrium}$

№	RT, min	Area, mV*сек	Equilibrium concentration, µM	Name
**1**	4.26	1377.79	150.41	Hyp
**2**	4.69	441.58	49.59	dIno
**3**	5.86	2365.79	152.849	Ade
**4**	7.81	2477.93	147.16	dAdo

Fugure S6.2. **1.5:1:0.25 ratio**

a) $t_{initial}$

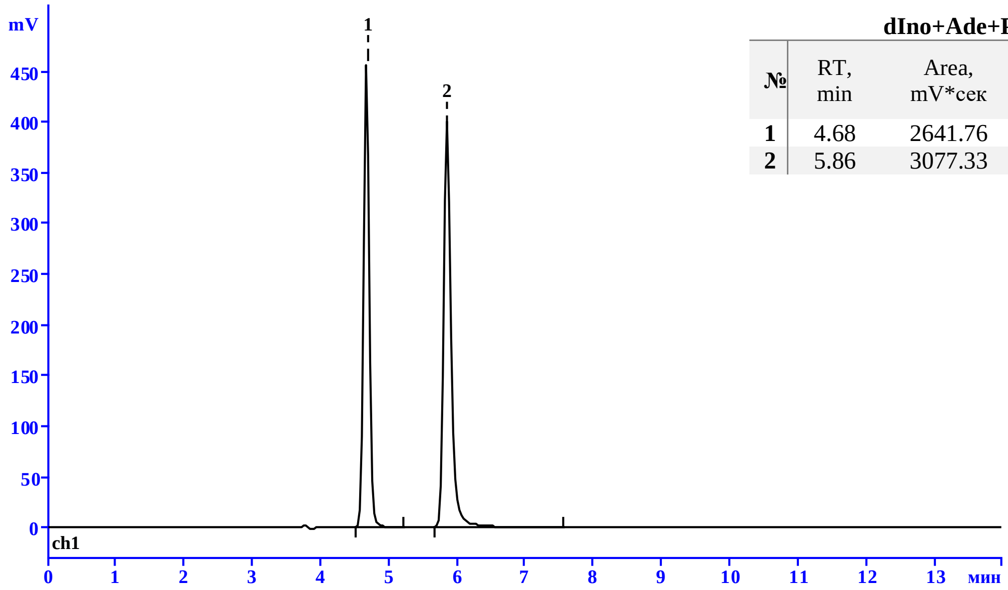

dIno+Ade+P_i – 1.5:1:0.25 – t_0

№	RT, min	Area, mV*сек	Equilibrium concentration, µM	Name
**1**	4.68	2641.76	300.00	dIno
**2**	5.86	3077.33	200.00	Ade

b) t$_{equilibrium}$

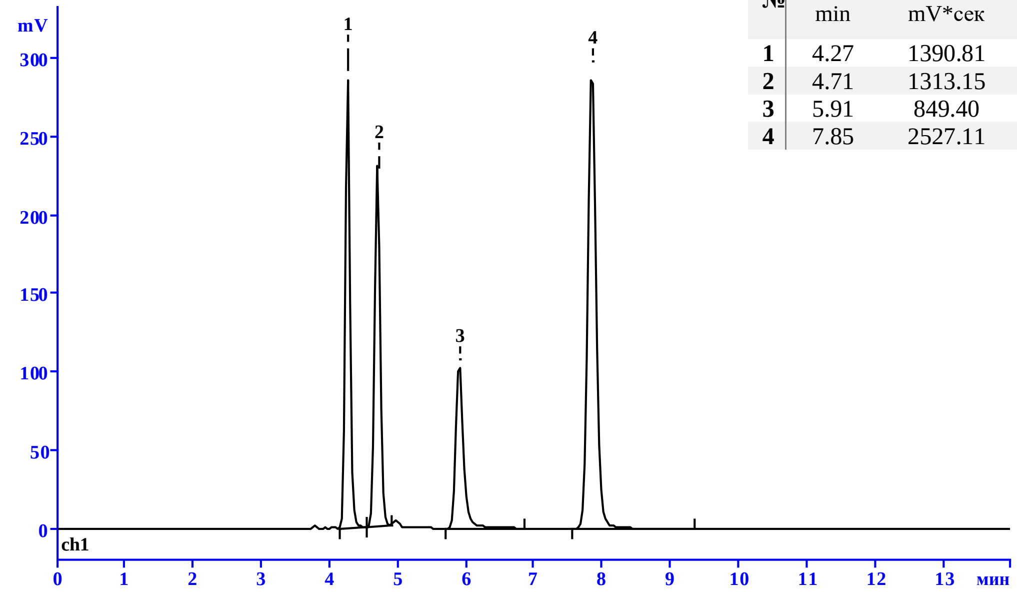

dIno+Ade+P$_i$ –1.5:1:0.25 – t $_{equilibrium}$

№	RT, min	Area, mV*сек	Equilibrium concentration, µM	Name
**1**	4.27	1390.81	150.88	Hyp
**2**	4.71	1313.15	149.12	dIno
**3**	5.91	849.40	55.20	Ade
**4**	7.85	2527.11	144.80	dAdo

Fugure S6.3. **1.5:1:1 ratio**

a) t$_{initial}$

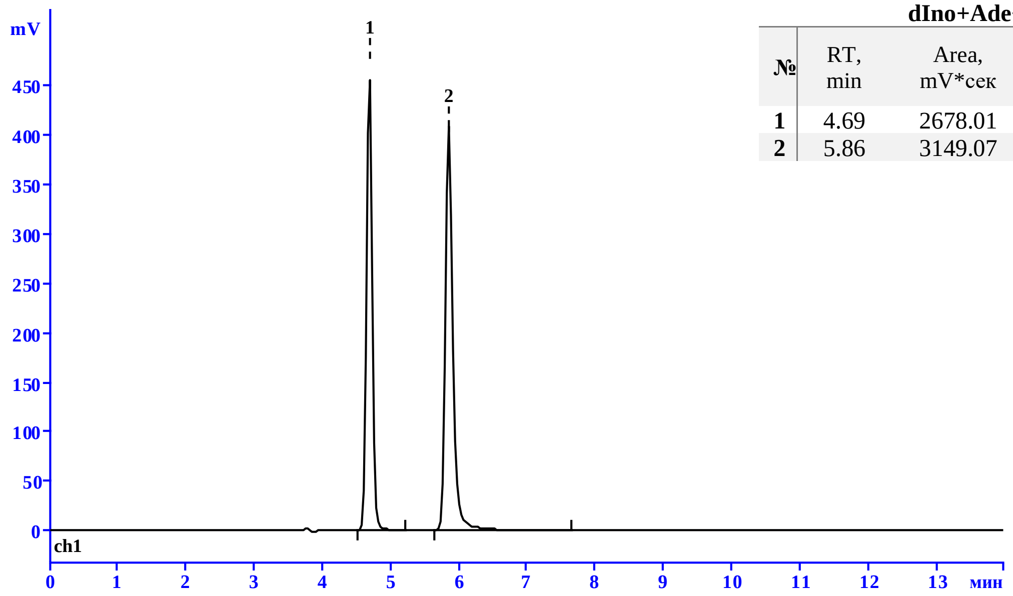

dIno+Ade+P$_i$ – 1.5:1:1 – t$_0$

№	RT, min	Area, mV*сек	Equilibrium concentration, µM	Name
**1**	4.69	2678.01	300.00	dIno
**2**	5.86	3149.07	200.00	Ade

b) $t_{equilibrium}$

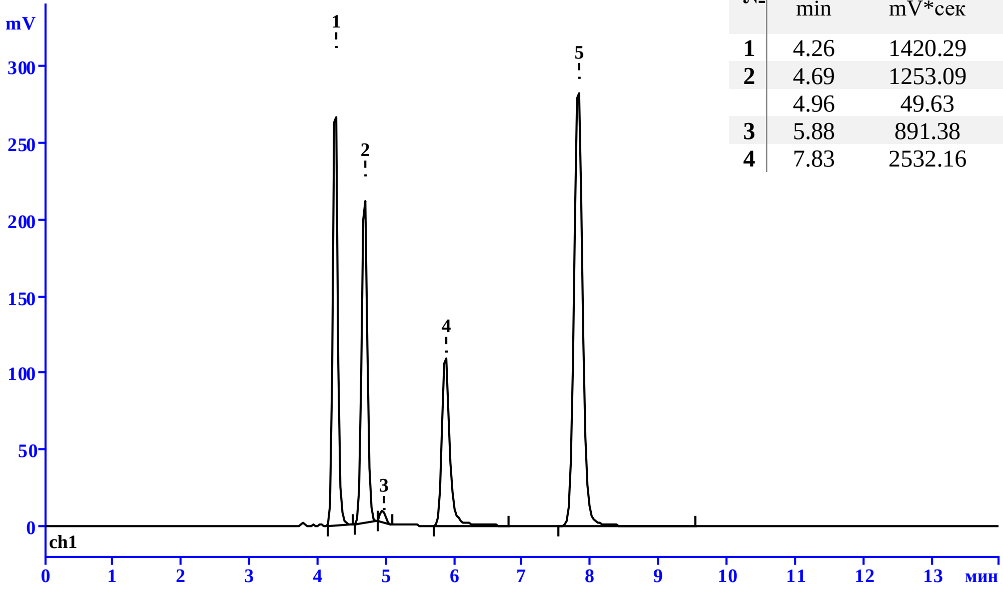

dIno+Ade+P_i –1.5:1:0.25 – t $_{equilibrium}$

№	RT, min	Area, mV*сек	Equilibrium concentration, µM	Name
**1**	4.26	1420.29	159.62	Hyp
**2**	4.69	1253.09	140.38	dIno
	4.96	49.63	-	-
**3**	5.88	891.38	55.61	Ade
**4**	7.83	2532.16	143.39	dAdo

Fugure S6.4. **3:1:1 ratio**

a) $t_{initial}$

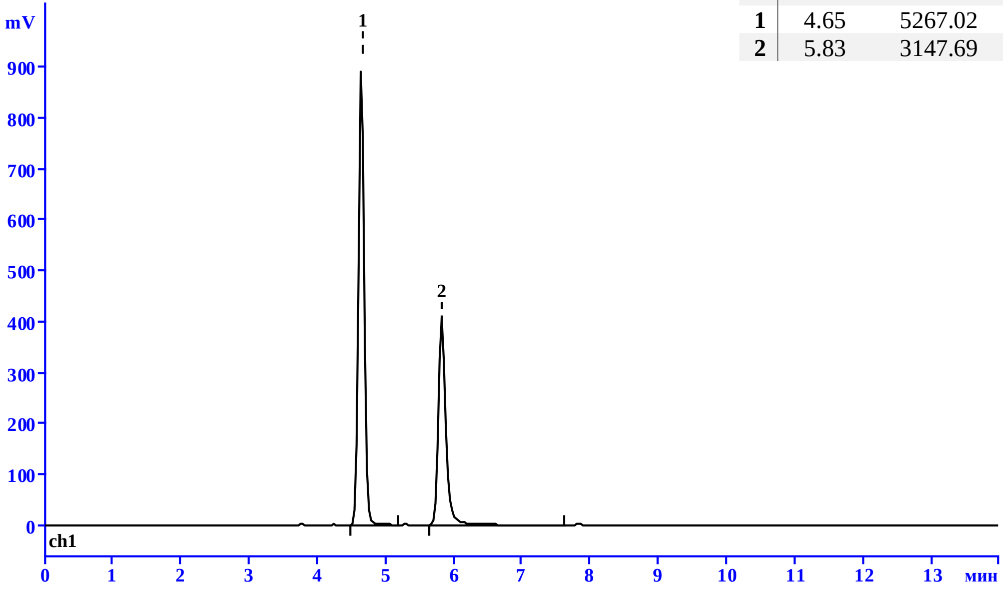

dIno+Ade+P_i – 3:1:1 – t_0

№	RT, min	Area, mV*сек	Equilibrium concentration, µM	Name
**1**	4.65	5267.02	600.00	dIno
**2**	5.83	3147.69	200.00	Ade

b) t$_{equilibrium}$

dIno+Ade+P$_i$ –3:1:0.25 – t $_{equilibrium}$

№	RT, min	Area, mV*сек	Equilibrium concentration, µM	Name
**1**	4.27	1755.63	198.71	Hyp
**2**	4.71	3522.64	401.29	dIno
	4.98	134.14	-	-
**3**	5.91	467.26	29.69	Ade
**4**	7.90	3063.01	170.31	dAdo

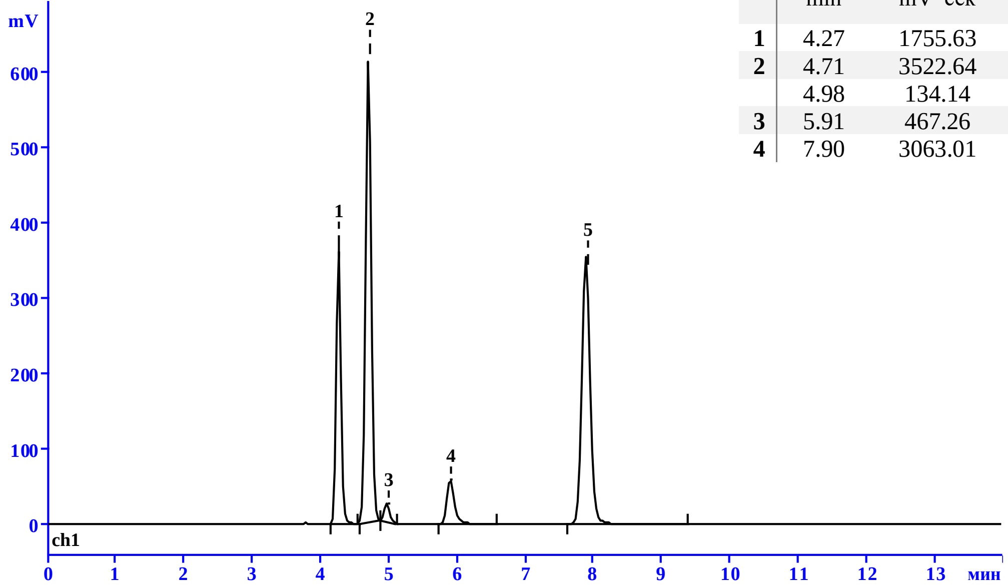

<u>2´-deoxyadenosine from 2´-deoxyguanosine (dGuo+Ade+P$_i$, analytical method)</u>

To a reaction sample solution (1 mL, Table S7) were added 1 U of PNP *E. coli*. The reaction mixture was incubated at 37°C, pH 7.5, and was monitored by HPLC.

Table S7. Volumes of stock and buffer solutions taken for 1 mL of a rection mixture (pH 7.5).

1 mM dGuo stock solution	2 mM Ade stock solution	5 mM phosphate	50 mM Tris - HCl	C_{dGuo}:C_{Ade}:C_P
200 µL	150 µL	40 µL	610 µL	1:1.5:1
300 µL	100 µL	10 µL	590 µL	1.5:1:0.25
300 µL	100 µL	40 µL	560 µL	1.5:1:1
600 µL	100 µL	40 µL	260 µL	3:1:1

HPLC-analysis of transglycosylation: Cosmosil 5CN-MS, 4.6*250 mm, 5µm, 300 Å, (Nacalai Tesque, Inc. (Japan)), 20ºC, elution in modified mobile phase: linear gradient of acetonitrile in 10 mM NaOAc/deionized water from 2 to 12% for 10 min (flushing with 12-80 % acetonitrile-10 mM NaOAc/deionized water for 10-10.1 min, then 80-2% for 10.1-10.8 min) at flow rate 1 ml/min with UV detection at wavelength 260 nm, injection volume 20 µL.

Fugure S7.1. **1:1.5:1 ratio**

a) $t_{initial}$

dGuo+Ade+P_i – 1:1.5:1 – t_0

№	RT, min	Area, mV*сек	Equilibrium concentration, µM	Name
**1**	5.41	3061.12	200.00	dGuo
**2**	5.91	4573.26	300.00	Ade

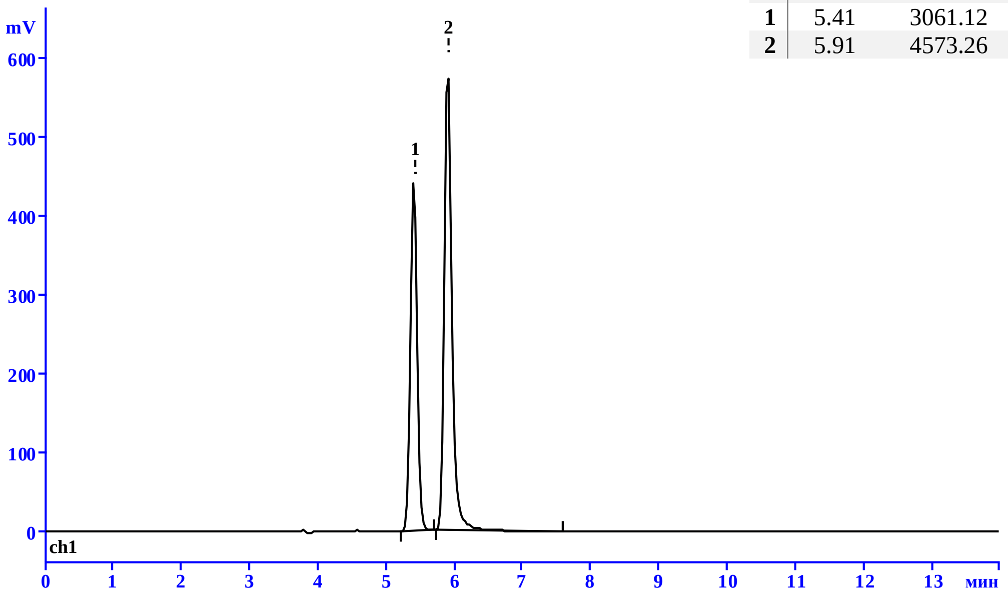

b) $t_{equilibrium}$

dGuo+Ade +P_i – 1:1.5:1 – t $_{equilibrium}$

№	RT, min	Area, mV*сек	Equilibrium concentration, µM	Name
**1**	4.59	1147.44	125.22	Gua
**2**	5.44	1144.51	74.78	dGuo
**3**	5.93	2680.66	175.85	Ade
**4**	7.96	2214.29	124.15	dAdo

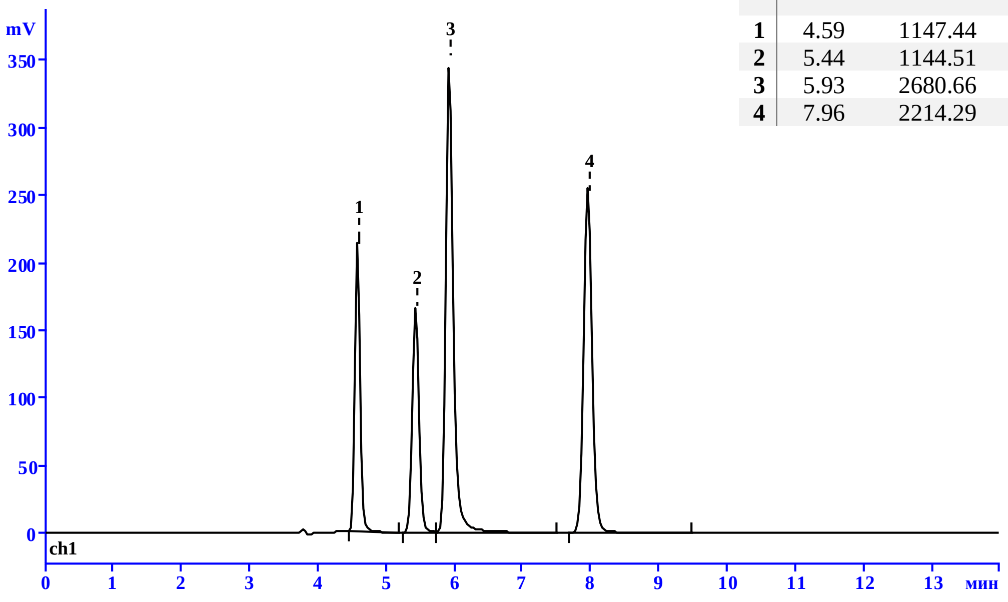

Fugure S7.2. **1.5:1:0.25 ratio**

a) $t_{initial}$

dGuo+Ade+P_i – 1.5:1:0.25 – t_0

№	RT, min	Area, mV*сек	Equilibrium concentration, μM	Name
**1**	5.35	4585.69	300.00	dGuo
**2**	5.85	3078.92	200.00	Ade

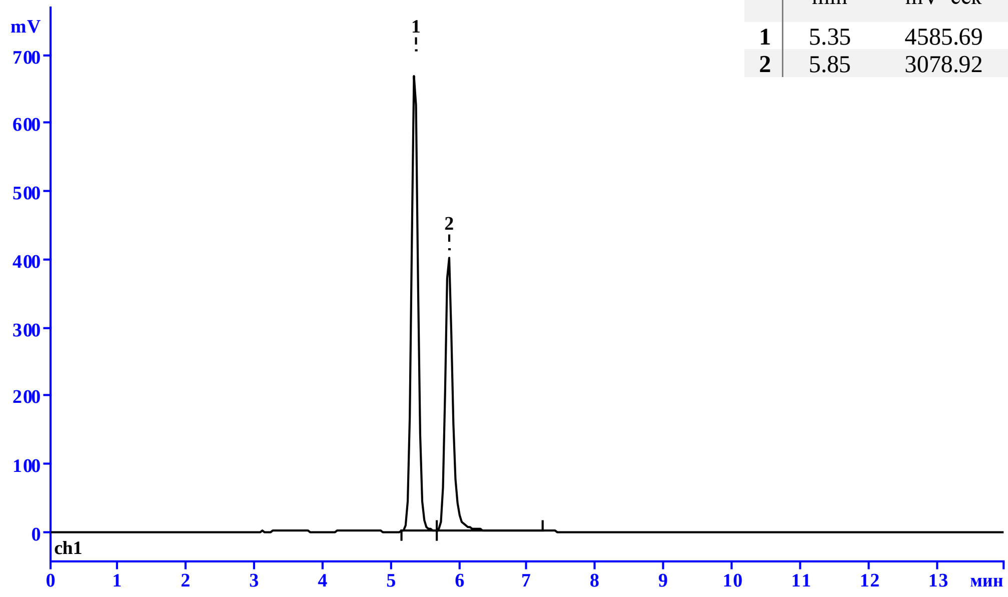

b) $t_{equilibrium}$

dGuo+Ade +P_i – 1.5:1:0.25– t $_{equilibrium}$

№	RT, min	Area, mV*сек	Equilibrium concentration, μM	Name
**1**	4.56	1146.06	134.19	Gua
**2**	5.41	2534.47	165.81	dGuo
**3**	5.92	1190.83	77.35	Ade
**4**	7.93	2171.77	122.65	dAdo

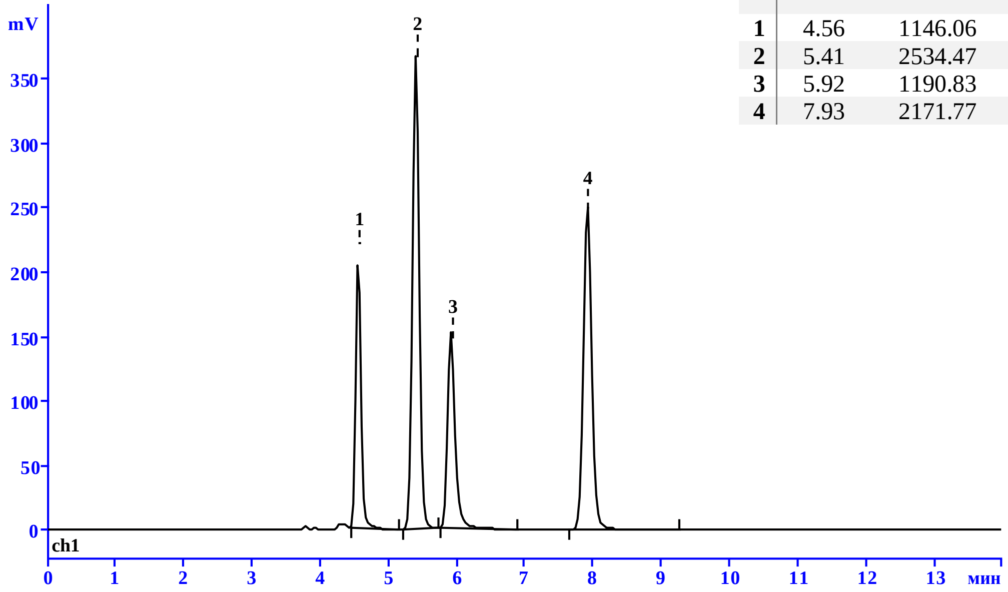

Fugure S7.3. **1.5:1:1 ratio.**

a) t_{initial}

dGuo+Ade+P_i – 1.5:1:1 – t_0

№	RT, min	Area, mV*сек	Equilibrium concentration, µM	Name
**1**	5.41	4461.30	300.00	dGuo
**2**	5.91	3156.17	200.00	Ade

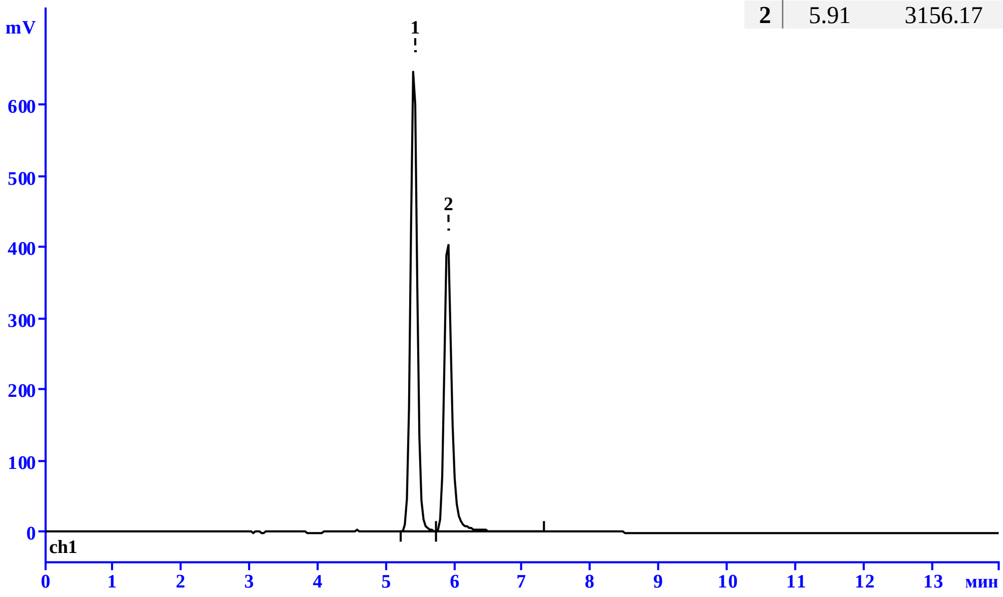

b) t_{equilibrium}

dGuo+Ade +P_i – 1.5:1:1– t equilibrium

№	RT, min	Area, mV*сек	Equilibrium concentration, µM	Name
**1**	4.54	1143.62	138.77	Gua
**2**	5.37	2397.63	161.23	dGuo
**3**	5.89	1255.35	79.55	Ade
**4**	7.91	2145.86	120.45	dAdo

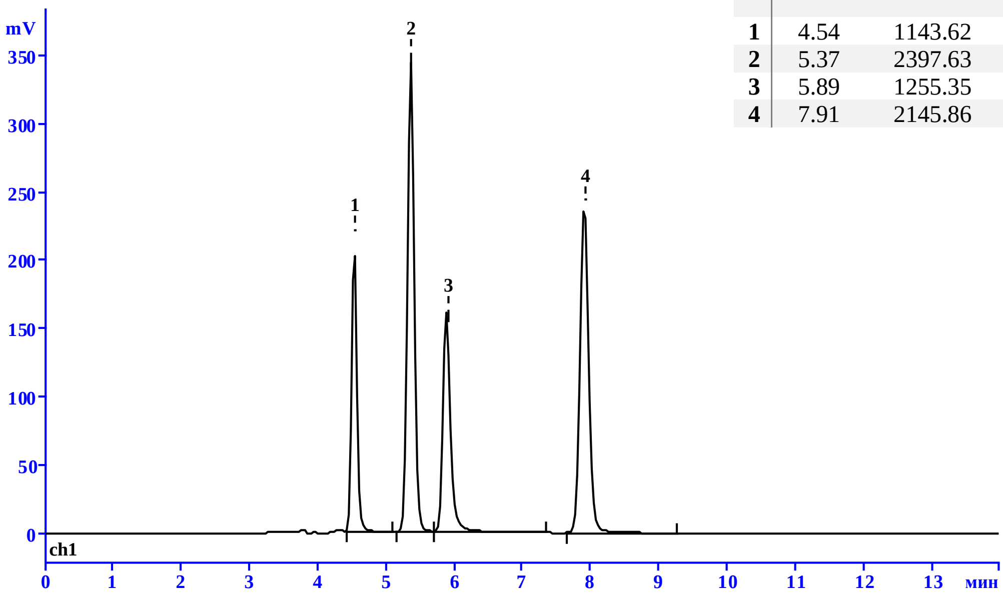

Fugure S7.4. **3:1:1 ratio.**

a) t

a) $t_{initial}$

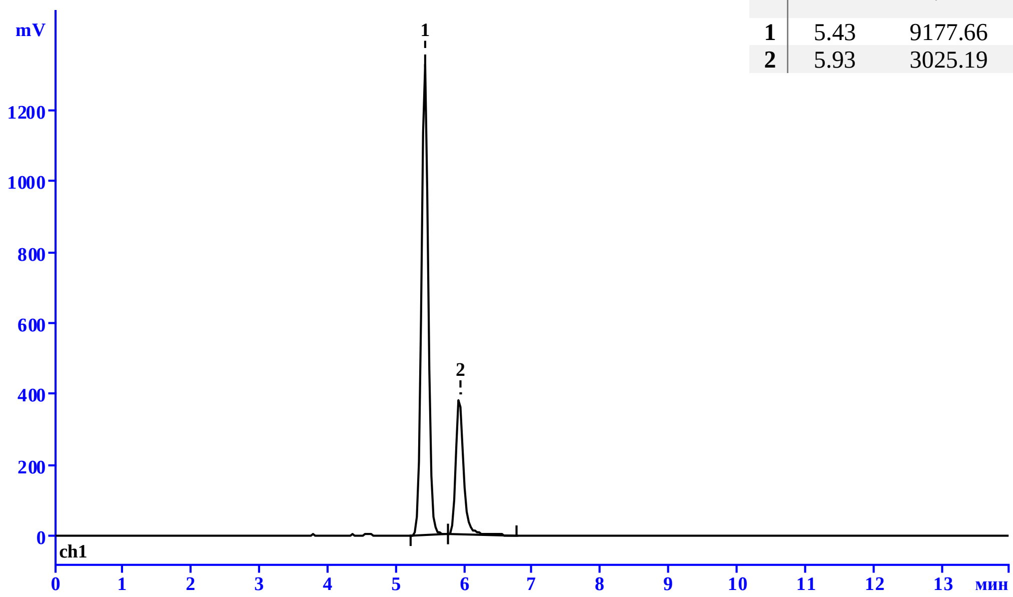

dGuo+Ade+P_i – 3:1:1 – t_0

№	RT, min	Area, mV*сек	Equilibrium concentration, µM	Name
1	5.43	9177.66	600.00	dGuo
2	5.93	3025.19	200.00	Ade

b) $t_{equilibrium}$

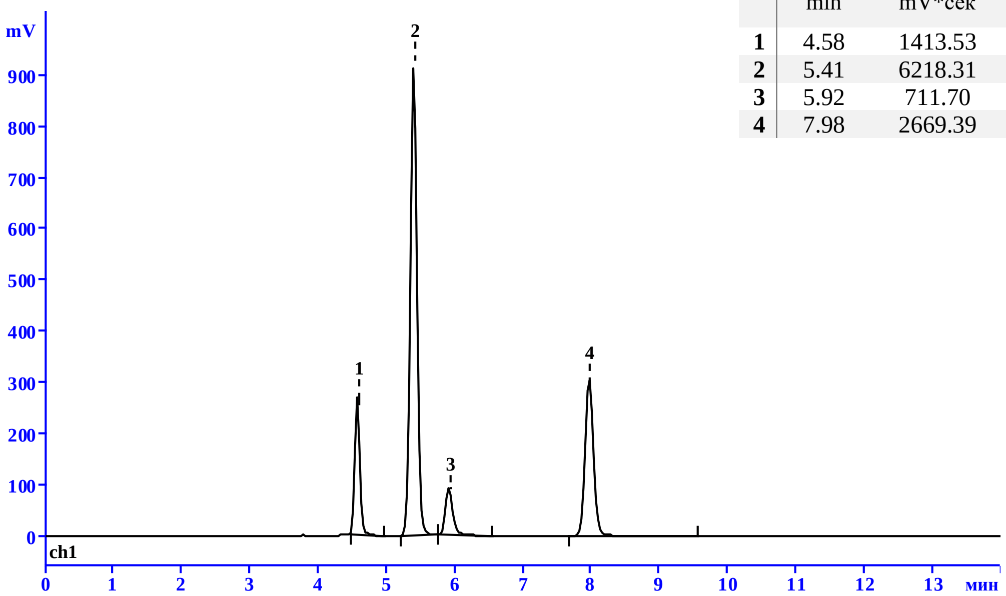

dGuo+Ade +P_i – 3:1:1– t $_{equilibrium}$

№	RT, min	Area, mV*сек	Equilibrium concentration, µM	Name
1	4.58	1413.53	193.47	Gua
2	5.41	6218.31	406.59	dGuo
3	5.92	711.70	47.05	Ade
4	7.98	2669.39	152.95	dAdo

2′-deoxyinosine from 2′-deoxyguanosine (dGuo+Hyp+P$_i$, analytical method)

To a reaction sample solution (1 mL, Table S8) were added 1 U of PNP *E. coli*. The reaction mixture was incubated at 37°C, pH 7.5, and was monitored by HPLC.

Table S8. Volumes of stock and buffer solutions taken for 1 mL of a rection mixture (pH 7.5).

1 mM dGuo stock solution	2 mM Hyp stock solution	5 mM phosphate	50 mM Tris - HCl	C_{dGuo}:C_{Hyp}:C_P
200 μL	150 μL	40 μL	610 μL	1:1.5:1
300 μL	100 μL	10 μL	590 μL	1.5:1:0.25
300 μL	100 μL	40 μL	560 μL	1.5:1:1
600 μL	100 μL	40 μL	260 μL	3:1:1

HPLC-analysis of transglycosylation: Cosmosil 5CN-MS, 4.6*250 mm, 5μm, 300 Å, (Nacalai Tesque, Inc. (Japan)), 20°C, elution in modified mobile phase: linear gradient of acetonitrile in 10 mM NaOAc/deionized water from 2 to 12% for 10 min (flushing with 12-80 % acetonitrile-10 mM NaOAc/deionized water for 10-10.1 min, then 80-2% for 10.1-10.8 min) at flow rate 1 ml/min with UV detection at wavelength 260 nm, injection volume 20 μL.

Fugure S8.1. **1:1.5:1 ratio**

a) t$_{\textbf{initial}}$ (2%-12% CH_3CN, 10 min, 10 mM NaOAc/ H_2O)

dGuo+Hyp+P$_i$ – 1:1.5:1 – t$_0$

№	RT, min	Area, mV*сек	Equilibrium concentration, μM	Name
**1**	4.27	2872.20	300.00	Hyp
**2**	5.37	3050.44	200.00	dGuo

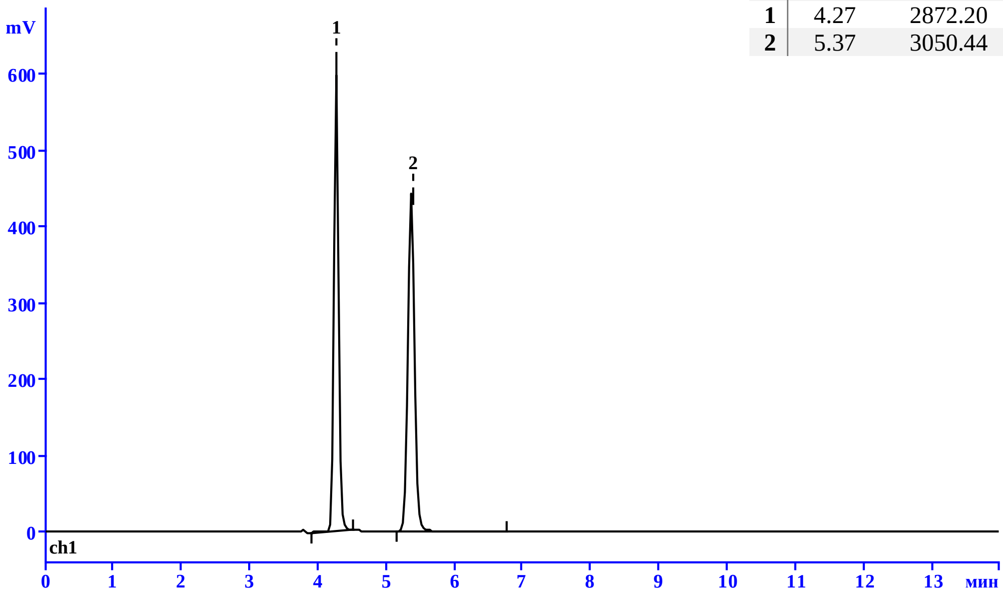

b) t$_{equilibrium}$ (0.1% CH_3CN, 10 min, 10 mM NaOAc/ H_2O)

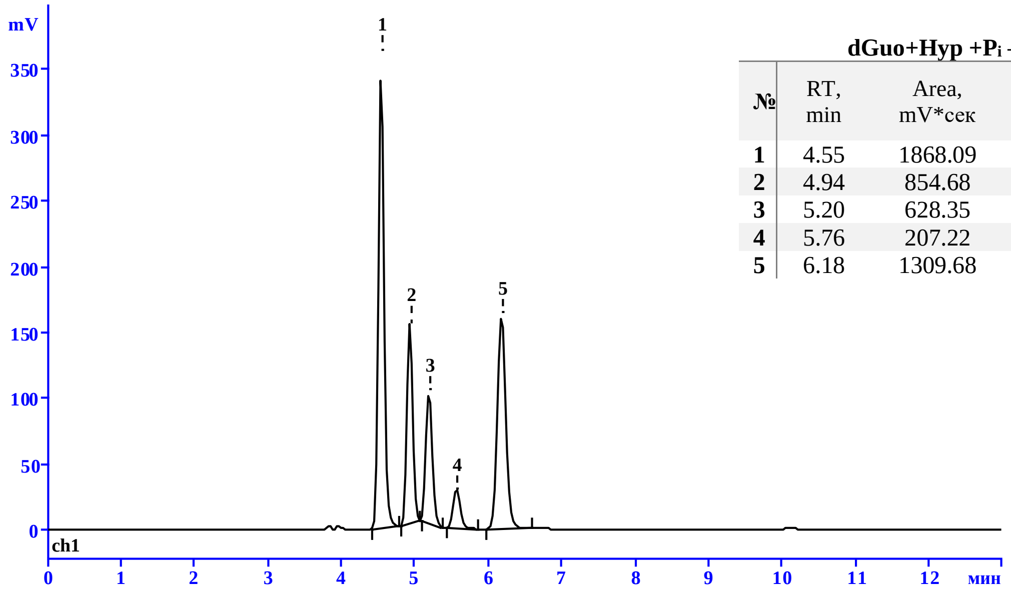

dGuo+Hyp +P$_i$ – 1:1.5:1– t $_{equilibrium}$

№	RT, min	Area, mV*сек	Equilibrium concentration, µM	Name
**1**	4.55	1868.09	195.12	Hyp
**2**	4.94	854.68	114.13	Gua
**3**	5.20	628.35	104.88	dIno
**4**	5.76	207.22	152.95	-
**5**	6.18	1309.68	85.87	dGuo

c) t$_{equilibrium}$ (2%-12% CH_3CN, 10 min, H_2O)

dGuo+Hyp +P$_i$ – 1:1.5:1– t $_{equilibrium}$

№	RT, min	Area, mV*сек	Equilibrium concentration, µM	Name
**1**	3.14	47.33	-	-
**2**	3.91	27.20	-	-
**3**	4.32	1916.94	200.22	Hyp
**4**	4.77	605.61	99.78	dIno
**5**	5.00	1114.85	120.48	Gua
**6**	5.52	1212.86	79.52	dGuo

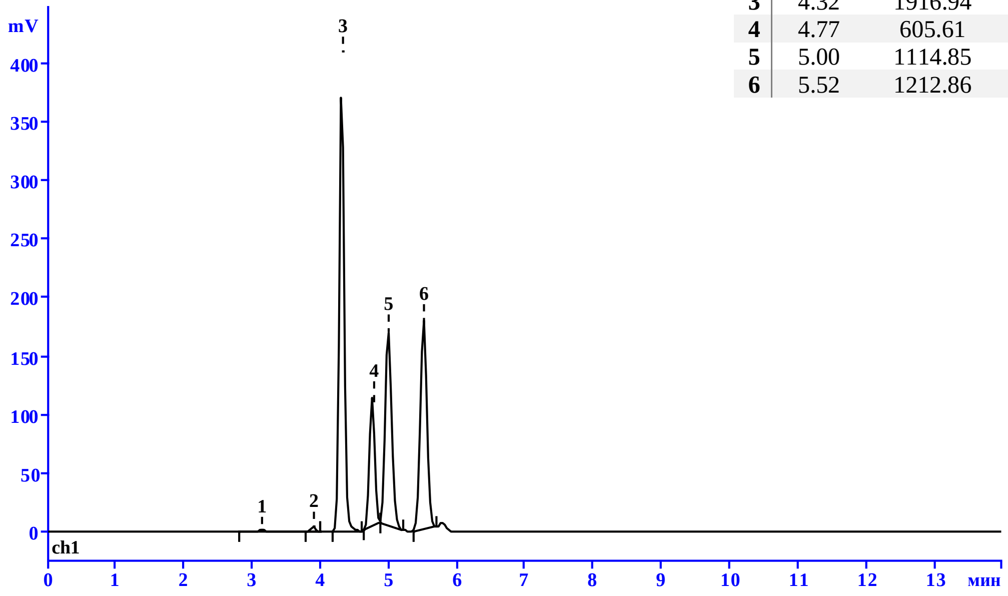

Fugure S8.2. **1.5:1:0.25 ratio**

a) $t_{initial}$ (2%-12% CH_3CN, 10 min, 10 mM NaOAc/ H_2O)

dGuo+Hyp+P_i – 1.5:1:0.25 – t_0

№	RT, min	Area, mV*сек	Equilibrium concentration, µM	Name
**1**	4.26	1862.70	200.00	Hyp
**2**	5.37	4537.34	300.00	dGuo

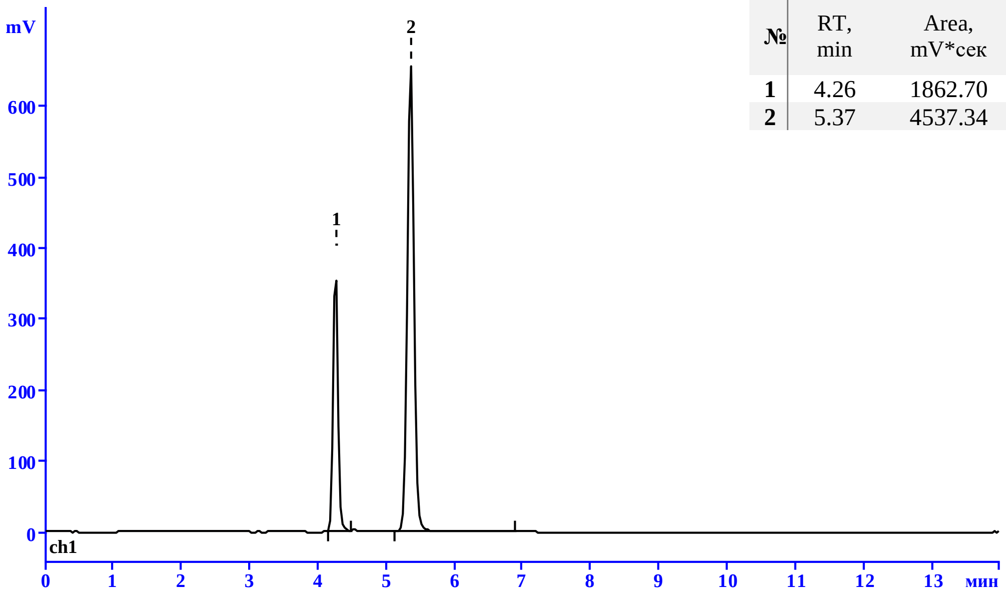

b) $t_{equilibrium}$ (0.1% CH_3CN, 10 min, 10 mM NaOAc/ H_2O)

dGuo+Hyp +P_i – 1.5:1:0.25– t $_{equilibrium}$

№	RT, min	Area, mV*сек	Equilibrium concentration, µM	Name
**1**	4.57	957.94	102.85	Hyp
**2**	4.97	836.16	121.05	Gua
**3**	5.24	692.65	97.15	dIno
**4**	5.62	118.85	-	-
**5**	6.24	2706.49	178.95	dGuo

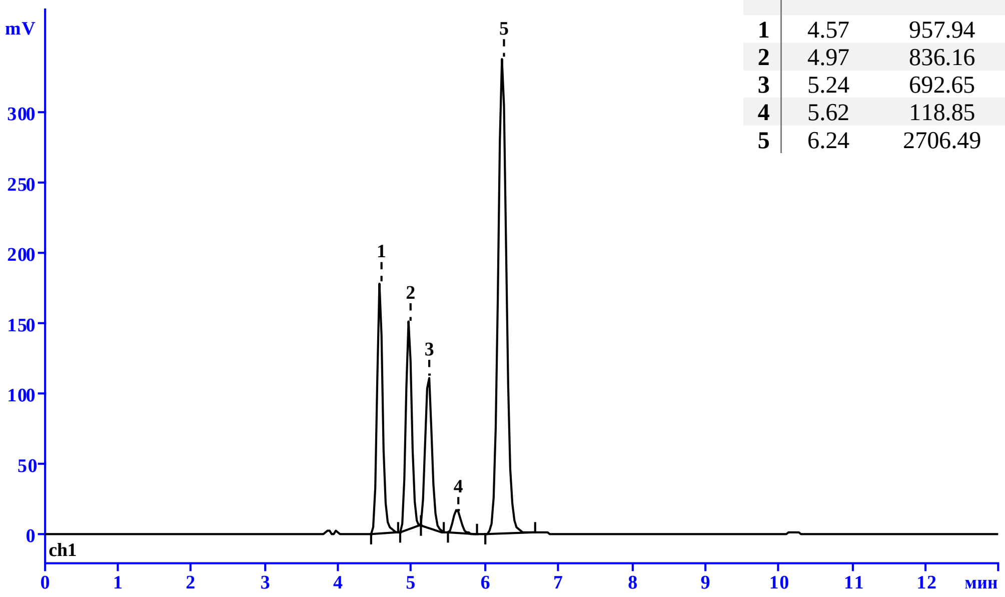

Fugure S8.3. **1.5:1:1 ratio**

a) $t_{initial}$ (2%-12% CH_3CN, 10 min, 10 mM NaOAc/ H_2O)

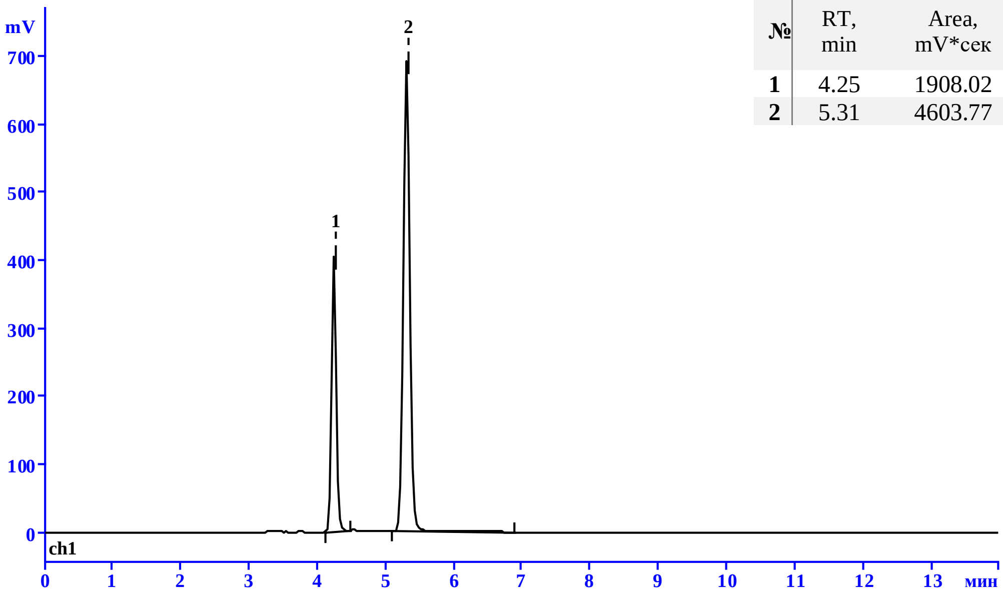

dGuo+Hyp+P_i – 1.5:1:1 – t_0

№	RT, min	Area, mV*сек	Equilibrium concentration, µM	Name
**1**	4.25	1908.02	200.00	Hyp
**2**	5.31	4603.77	300.00	dGuo

b) $t_{equilibrium}$ (0.1% CH_3CN, 10 min, 10 mM NaOAc/ H_2O)

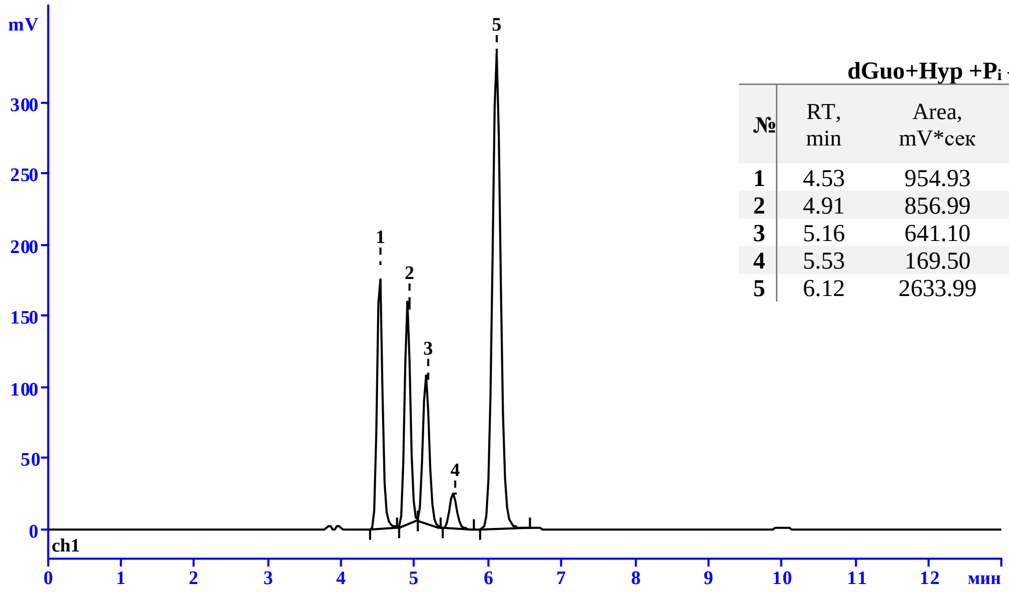

dGuo+Hyp +P_i – 1.5:1:1– t $_{equilibrium}$

№	RT, min	Area, mV*сек	Equilibrium concentration, µM	Name
**1**	4.53	954.93	100.10	Hyp
**2**	4.91	856.99	128.36	Gua
**3**	5.16	641.10	99.90	dIno
**4**	5.53	169.50	-	-
**5**	6.12	2633.99	171.64	dGuo

Fugure S8.4. **3:1:1 ratio**

a) $t_{initial}$ (2%-12% CH_3CN, 10 min, 10 mM NaOAc/ H_2O)

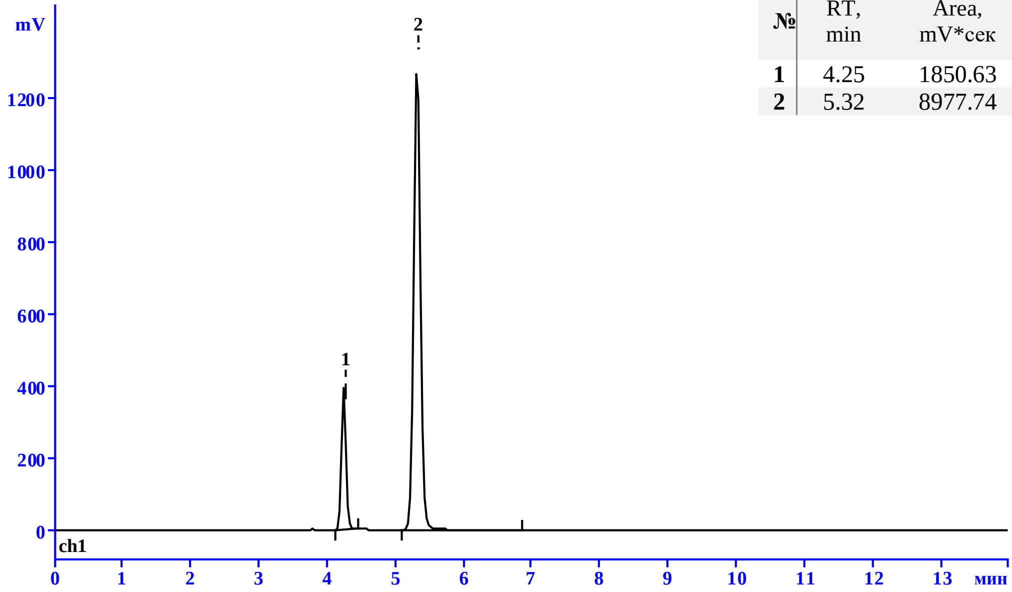

dGuo+Hyp+P_i – 3:1:1 – t_0

№	RT, min	Area, mV*сек	Equilibrium concentration, µM	Name
**1**	4.25	1850.63	200.00	Hyp
**2**	5.32	8977.74	600.00	dGuo

b) $t_{equilibrium}$ (0.1% CH_3CN, 10 min, 10 mM NaOAc/ H_2O)

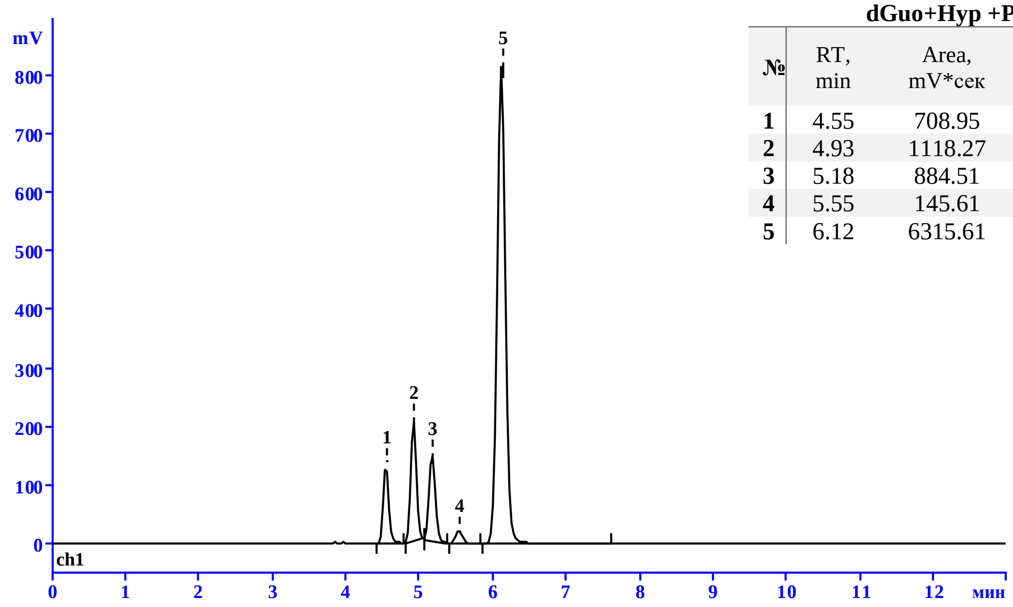

dGuo+Hyp +P_i – 3:1:1– t equilibrium

№	RT, min	Area, mV*сек	Equilibrium concentration, µM	Name
**1**	4.55	708.95	76.62	Hyp
**2**	4.93	1118.27	177.92	Gua
**3**	5.18	884.51	123.38	dIno
**4**	5.55	145.61	-	-
**5**	6.12	6315.61	422.08	dGuo

c) t$_{\textbf{equilibrium}}$ (2%-12% CH_3CN, 10 min, H_2O)

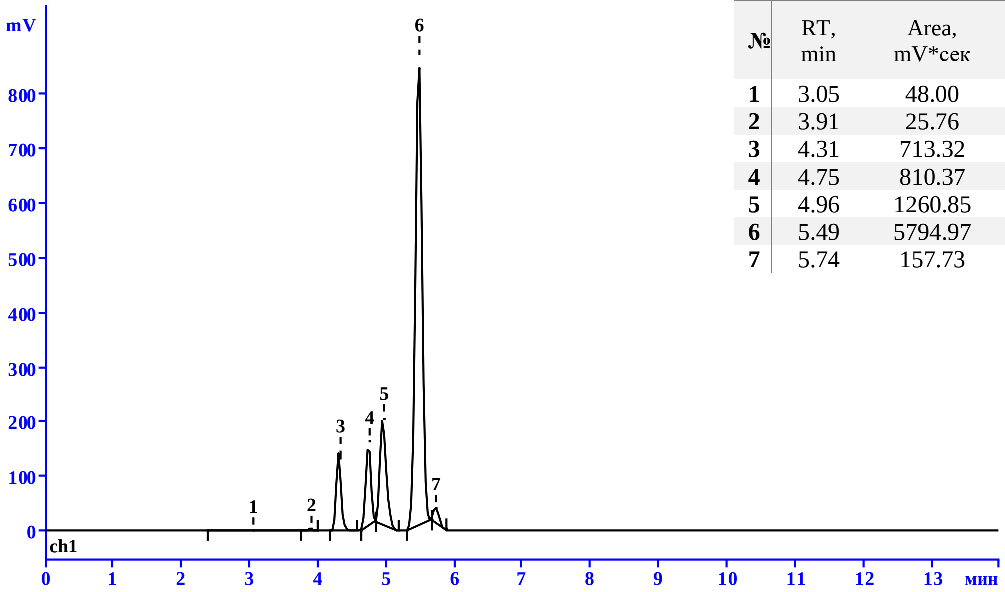

dGuo+Hyp +P_i – 3:1:1– t $_{\textbf{equilibrium}}$

№	RT, min	Area, mV*сек	Equilibrium concentration, μM	Name
**1**	3.05	48.00	-	-
**2**	3.91	25.76	-	-
**3**	4.31	713.32	77.09	Hyp
**4**	4.75	810.37	122.91	dIno
**5**	4.96	1260.85	212.71	Gua
**6**	5.49	5794.97	387.29	dGuo
**7**	5.74	157.73	-	-

2′-deoxyuridine from 7-methyl-2′-deoxyguanosine (7-Me-dGuo+Ura+P_i, analytical method)

To a reaction sample solution (1 mL, Table S9) were added 1 U of PNP *E. coli*. The reaction mixture was incubated at 37°C, pH 7.5, and was monitored by HPLC.

Table S9. Volumes of stock and buffer solutions taken for 1 mL of a rection mixture (pH 7.5).

1 mM 7-Me-dGuo stock solution	2 mM Ura stock solution	5 mM phosphate	50 mM Tris - HCl	$C_{7\text{-MedGuo}}$:C_{Ura}:C_{P}
200 μL	150 μL	40 μL	610 μL	1:1.5:1
300 μL	100 μL	10 μL	590 μL	1.5:1:0.25
300 μL	100 μL	40 μL	560 μL	1.5:1:1
600 μL	100 μL	40 μL	260 μL	3:1:1

HPLC-analysis of transglycosylation: Cosmosil 5C18-MS-II, 4.6*150 mm, 5µm, 120 Å, (Nacalai Tesque, Inc. (Japan)), 20°C, elution in modified mobile phase: linear gradient of acetonitrile in 0.06% TFA/deionized water from 2 to 12% for 10 min (flushing with 12-80 % acetonitrile-0.06% TFA/deionized water for 10-10.1 min, then 80-2% for 10.1-10.8 min) at flow rate 1 ml/min with UV detection at wavelength 260 nm, injection volume 20 µL.

Fugure S9.1. **1:1.5:1 ratio**

a) $t_{initial}$

7-MedGuo+Ura +P_i – 1:1.5:1 – t_0

№	RT, min	Area, mV*сек	Equilibrium concentration, µM	Name
**1**	2.82	2533.16	300.00	Ura
**2**	3.95	50.97	-	-
**3**	4.44	134.23	16.60	7-Me-Gua
**4**	7.21	2291.99	200.00	7-Me-dGuo

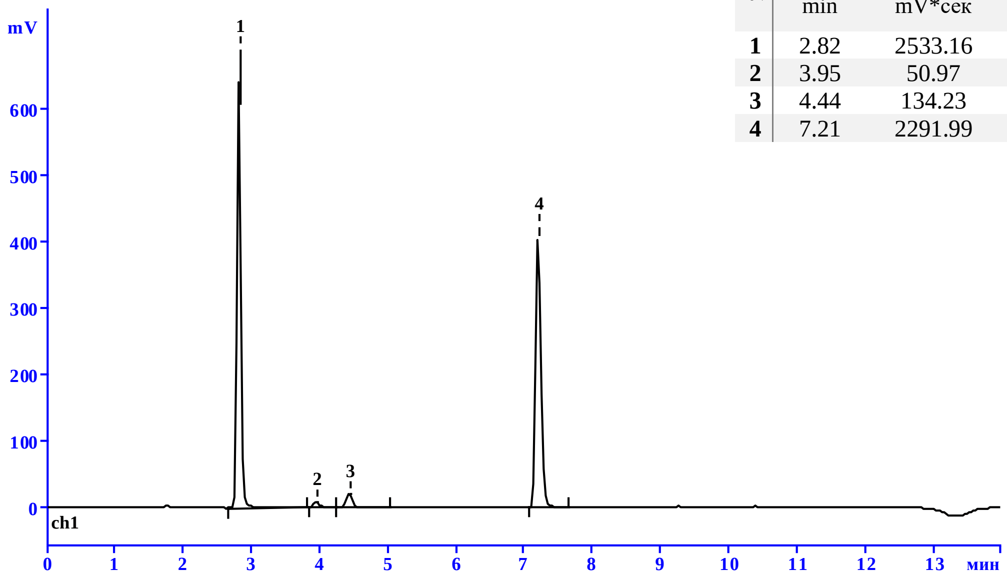

b) $t_{equilibrium}$

7-MedGuo+Ura +P_i – 1:1.5:1 – $t_{equilibrium}$

№	RT, min	Area, mV*сек	Equilibrium concentration, µM	Name
**1**	2.85	1580.03	187.12	Ura
**2**	3.96	50.91	-	-
**3**	4.44	1760.63	216.60	7-Me-Gua
**4**	6.51	1163.86	112.88	dUrd

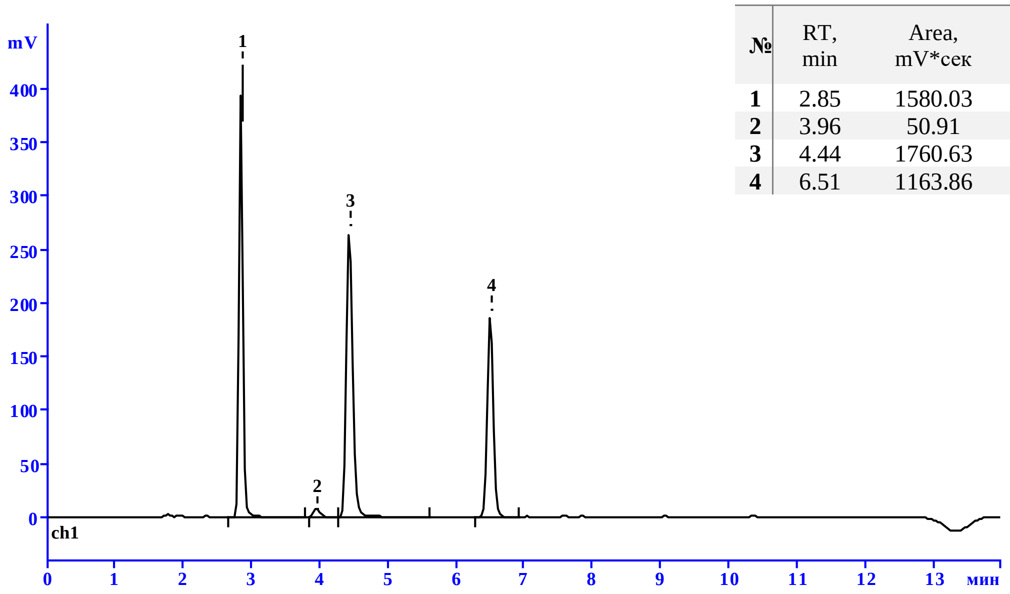

Fugure S9.2. **1.5:1:0.25 ratio**

a) $t_{initial}$

7-MedGuo+Ura +P_i – 1.5:1:0.25 – t_0

№	RT, min	Area, mV*сек	Equilibrium concentration, µM	Name
**1**	2.78	1173.79	200.00	Ura
**2**	3.86	67.76	-	-
**3**	4.33	149.51	18.50	7-Me-Gua
**4**	7.06	3166.80	300.00	7-Me-dGuo

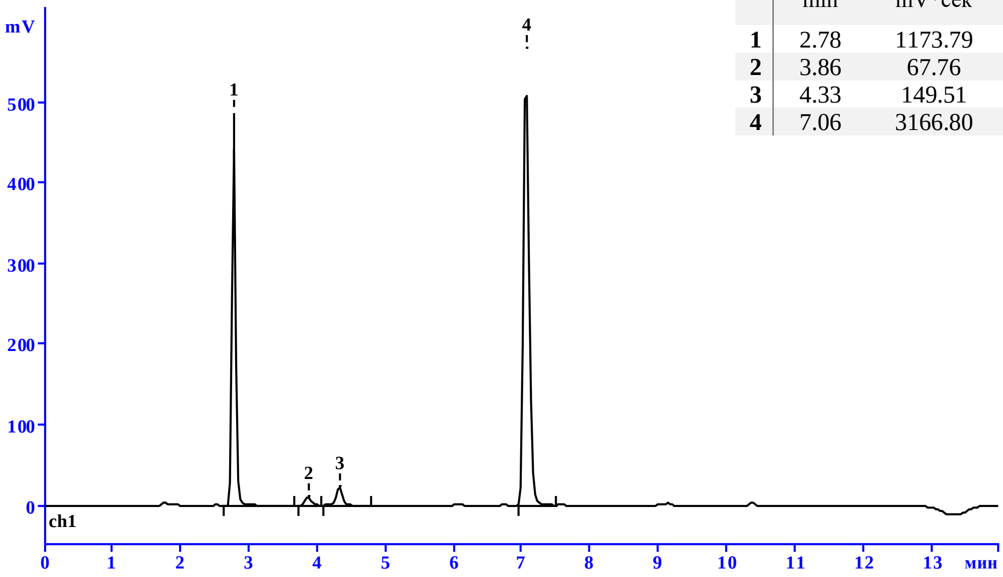

b) $t_{equilibrium}$

7-MedGuo+Ura +P_i – 1.5:1:0.25 – $t_{equilibrium}$

№	RT, min	Area, mV*сек	Equilibrium concentration, µM	Name
**1**	2.81	656.04	73.97	Ura
**2**	3.91	70.36	-	-
**3**	4.37	2393.30	318.50	7-Me-Gua
**4**	6.21	1374.44	126.03	dUrd

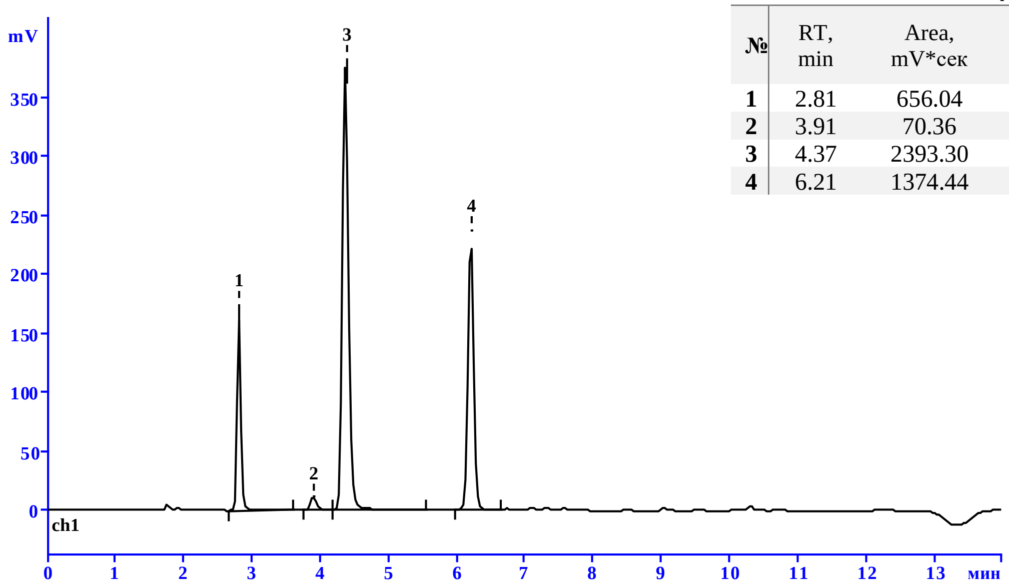

Fugure S9.3. **1.5:1:1 ratio**

a) t_{initial}

7-MedGuo+Ura +P_i – 1.5:1:1 – t_0

№	RT, min	Area, mV*сек	Equilibrium concentration, µM	Name
**1**	1.75	21.60	-	-
**2**	2.74	1675.59	200.00	Ura
**3**	3.79	75.99	-	-
**4**	4.25	184.13	22.78	7-Me-Gua
**5**	7.09	3579.23	300.00	7-Me-dGuo
**6**	10.40	29.12	-	-

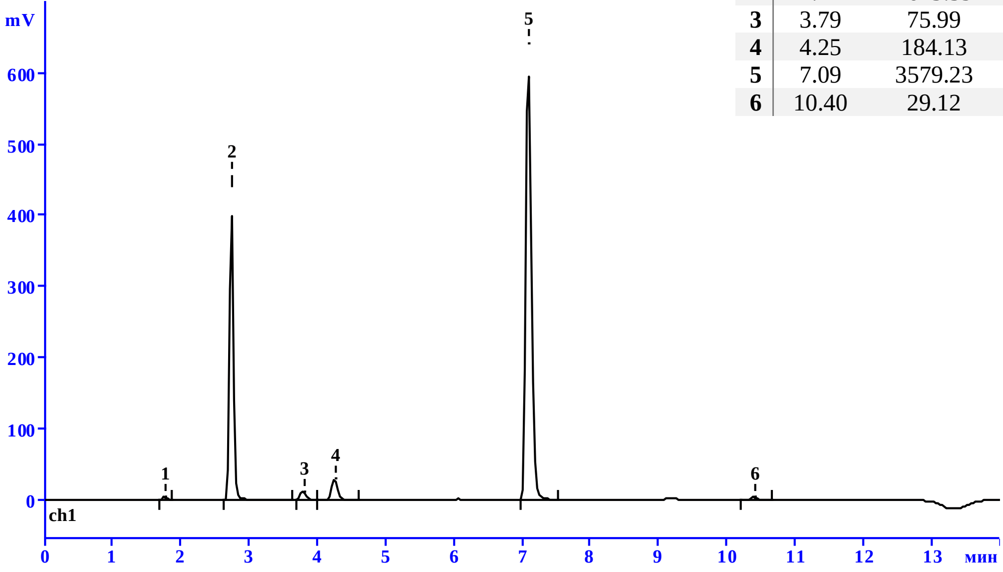

b) t_{equilibrium}

7-MedGuo+Ura +P_i – 1.5:1:1 – t equilibrium

№	RT, min	Area, mV*сек	Equilibrium concentration, µM	Name
**1**	1.76	22.63	-	-
2	2.83	615.76	73.50	Ura
**3**	3.93	77.16	-	-
**4**	4.37	2712.33	322.78	7-Me-Gua
**5**	6.31	1265.46	126.50	dUrd

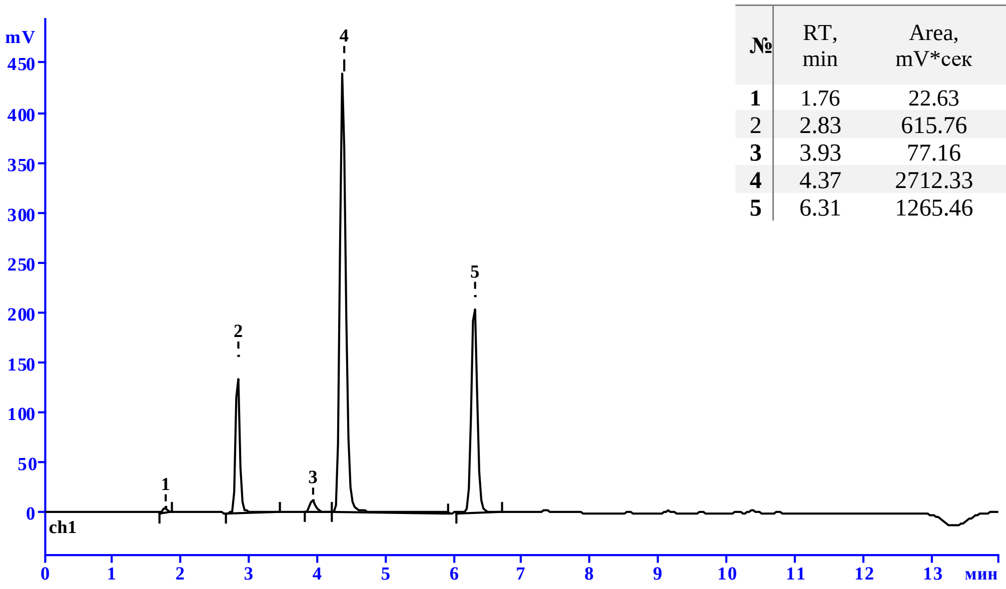

Fugure S9.4. **3:1:1 ratio**

a) $t_{initial}$

7-MedGuo+Ura +P_i – 3:1:1 – t_0

№	RT, min	Area, mV*сек	Equilibrium concentration, µM	Name
**1**	1.76	40.44	-	-
**2**	2.78	1550.86	200.00	Ura
**3**	3.74	164.34	-	-
**4**	4.19	408.52	50.53	7-Me-Gua
**5**	7.01	7902.00	600.00	7-Me-dGuo
**6**	9.13	30.27	-	-
**7**	10.33	63.77	-	-

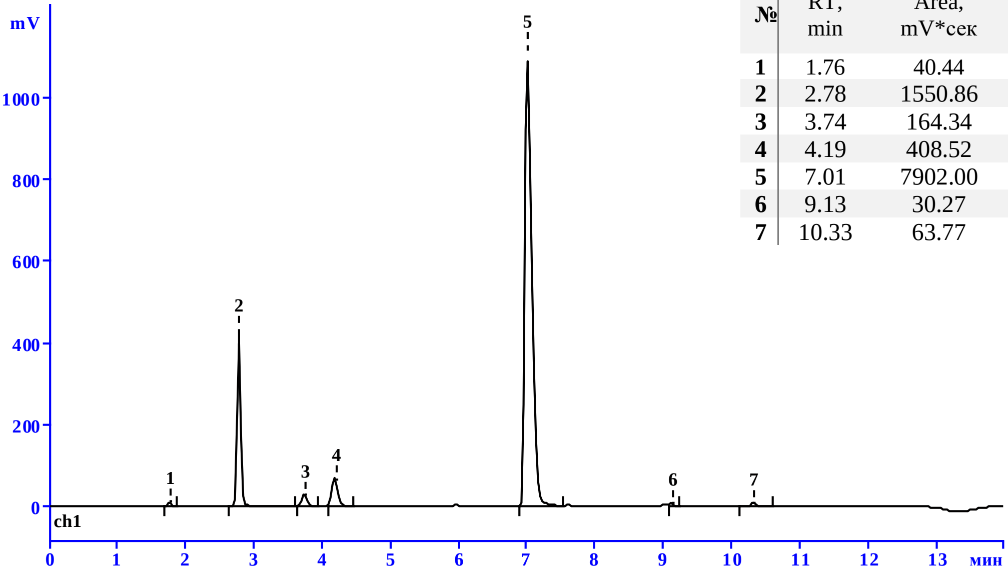

b) $t_{equilibrium}$

7-MedGuo+Ura +P_i – 3:1:1 – t $_{equilibrium}$

№	RT, min	Area, mV*сек	Equilibrium concentration, µM	Name
**1**	1.77	44.70	-	-
2	2.85	191.91	18.09	Ura
**3**	3.95	170.07	-	-
**4**	4.42	6054.85	644.04	7-Me-Gua
**5**	6.39	1676.53	181.91	dUrd
**6**	7.24	85.88	6.52	7-Me-dGuo
**7**	7.44	35.45	-	-
**8**	9.09	29.31	-	-
**9**	10.29	44.35	-	-

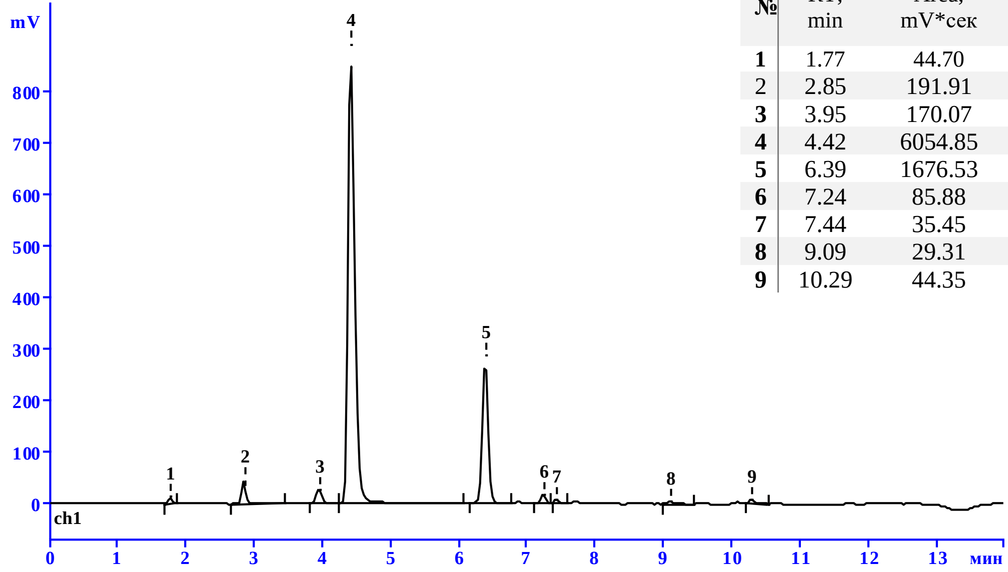

2′-deoxyinosine from 7-methyl-2′-deoxyguanosine (7-Me-dGuo+Hyp+P_i, analytical method)

To a reaction sample solution (1 mL, Table S10) were added 1 U of PNP *E. coli*. The reaction mixture was incubated at 37°C, pH 7.5, and was monitored by HPLC.

Table S10. Volumes of stock and buffer solutions taken for 1 mL of a rection mixture (pH 7.5).

1 mM 7-Me-dGuo stock solution	2 mM Hyp stock solution	5 mM phosphate	50 mM Tris - HCl	$C_{7\text{-MedGuo}}:C_{Hyp}:C_P$
200 µL	150 µL	40 µL	610 µL	1:1.5:1
300 µL	100 µL	10 µL	590 µL	1.5:1:0.25
300 µL	100 µL	40 µL	560 µL	1.5:1:1
600 µL	100 µL	40 µL	260 µL	3:1:1

HPLC-analysis of transglycosylation: Cosmosil 5C18-MS-II, 4.6*150 mm, 5µm, 120 Å, (Nacalai Tesque, Inc. (Japan)), 20°C, elution in modified mobile phase: linear gradient of acetonitrile in 0.06% TFA/deionized water from 2 to 12% for 10 min (flushing with 12-80 % acetonitrile-0.06% TFA/deionized water for 10-10.1 min, then 80-2% for 10.1-10.8 min) at flow rate 1 ml/min with UV detection at wavelength 260 nm, injection volume 20 µL.

Fugure S10.1. **1:1.5:1 ratio**

a) $t_{initial}$

7-MedGuo+Hyp+P_i – 1:1.5:1 – t_0

№	RT, min	Area, mV*сек	Equilibrium concentration, µM	Name
1	3.47	2872.54	300.00	Hyp
2	3.99	74.27	-	-
3	4.49	95.67	11.83	7-MeGua
4	7.37	2303.04	200.00	7-MedGuo

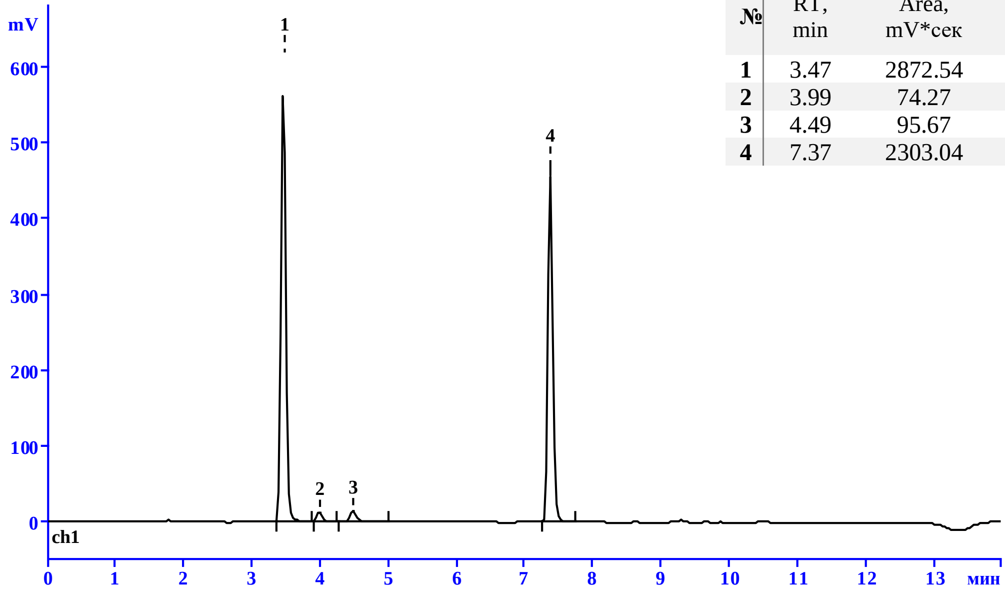

b) $t_{equilibrium}$

7-Me-dGuo+Hyp+P_i – 1:1.5:1 – t $_{equilibrium}$

№	RT, min	Area, mV*сек	Equilibrium concentration, µM	Name
**1**	3.34	1540.82	160.92	Hyp
**2**	3.79	78.44	-	-
**3**	4.27	1715.36	211.83	7-MeGua
**4**	7.93	1334.91	139.08	dIno

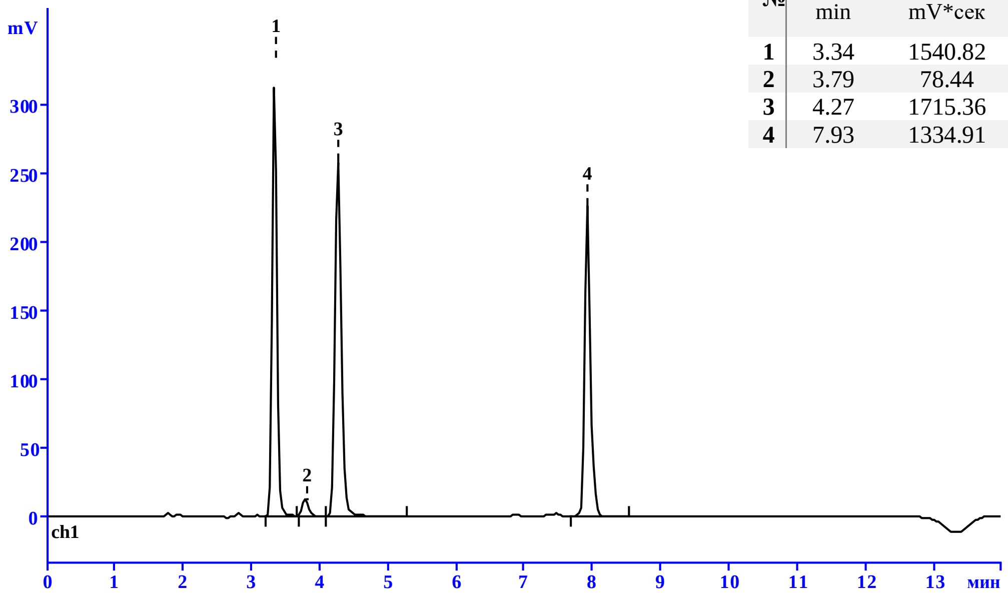

Fugure S10.2. **1.5:1:0.25 ratio.**

a) $t_{initial}$

7-MedGuo+Hyp+P_i – 1.5:1:0.25 – t_0

№	RT, min	Area, mV*сек	Equilibrium concentration, µM	Name
**1**	1.75	21.16	-	-
**2**	3.42	1905.90	200.00	Hyp
**3**	3.99	110.38	-	-
**4**	4.52	113.86	14.09	7-MeGua
**5**	7.24	3430.84	300.00	7-MedGuo

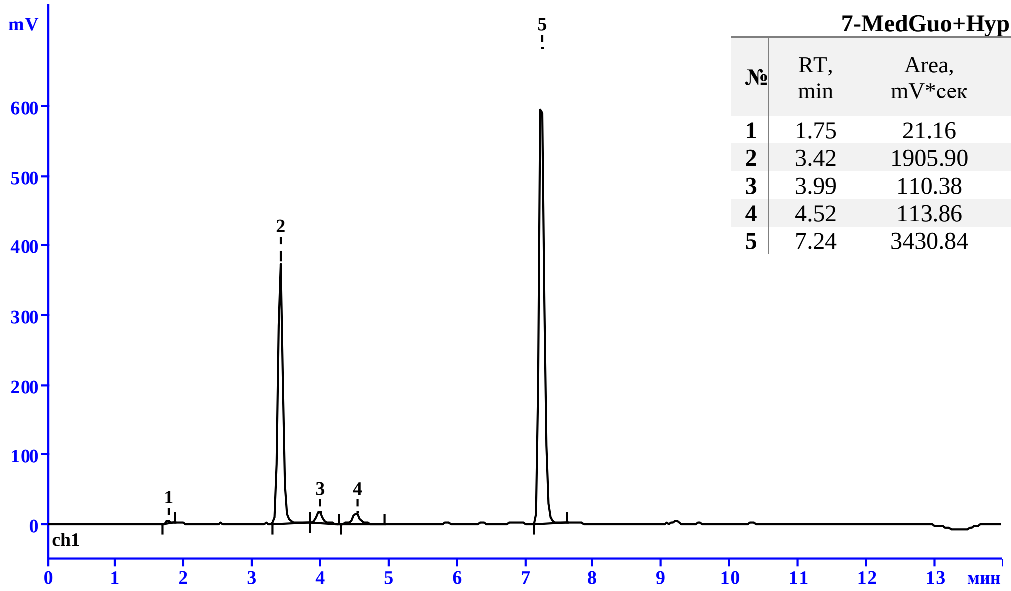

b) t_{equilibrium}

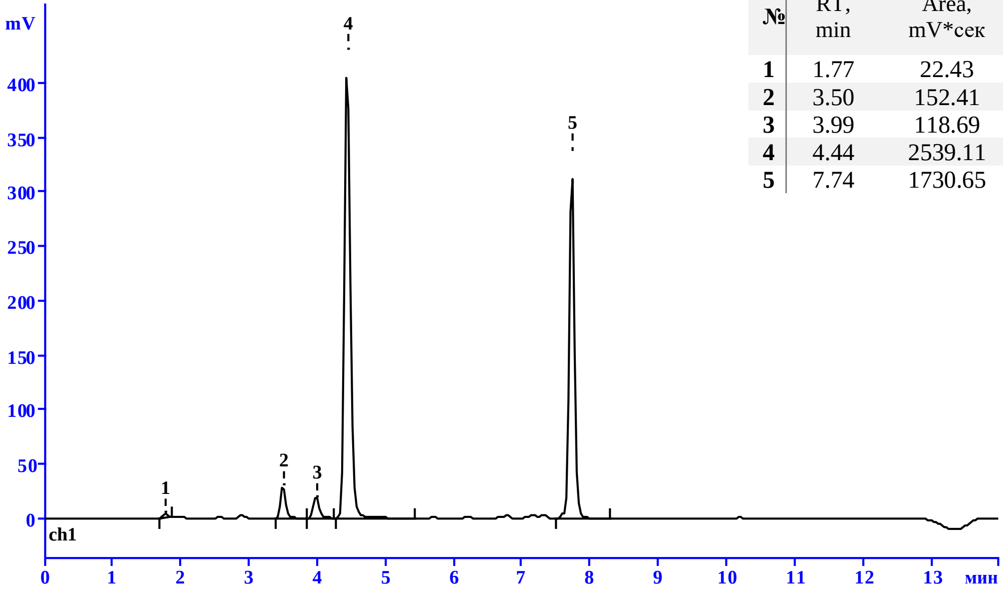

7-Me-dGuo+Hyp+P_i – 1.5:1:0.25 – t $_{equilibrium}$

№	RT, min	Area, mV*сек	Equilibrium concentration, μM	Name
**1**	1.77	22.43	-	-
**2**	3.50	152.41	15.99	Hyp
**3**	3.99	118.69	-	-
**4**	4.44	2539.11	314.09	7-MeGua
**5**	7.74	1730.65	184.01	dIno

Fugure S10.3. **1.5:1:1 ratio.**

a) t$_{initial}$

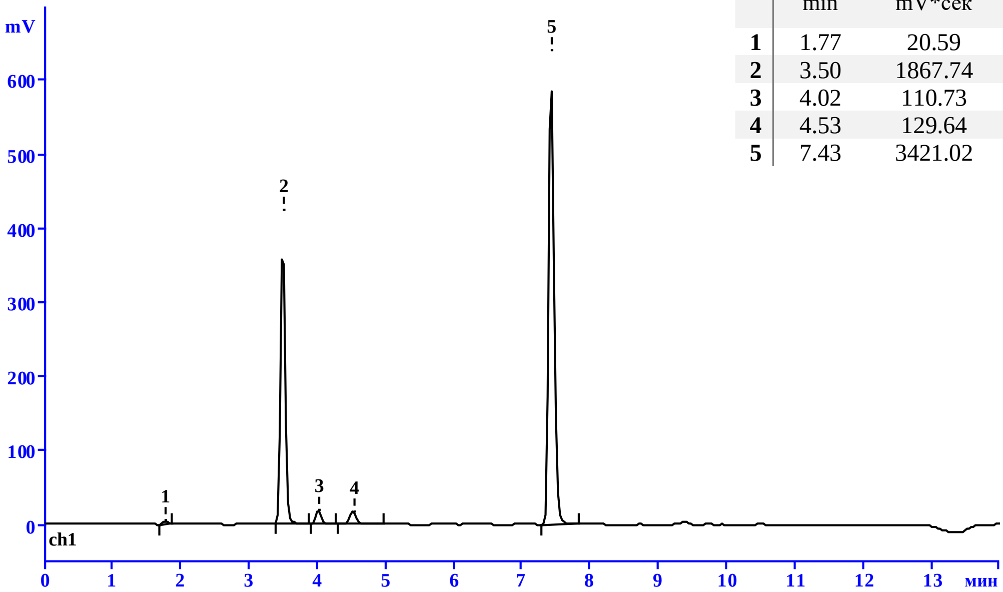

7-MedGuo+Hyp+P_i – 1.5:1:1 – t_0

№	RT, min	Area, mV*сек	Equilibrium concentration, μM	Name
**1**	1.77	20.59	-	-
**2**	3.50	1867.74	200.00	Hyp
**3**	4.02	110.73	-	-
**4**	4.53	129.64	16.04	7-Me-Gua
**5**	7.43	3421.02	300.00	7-MedGuo

b) tequilibrium

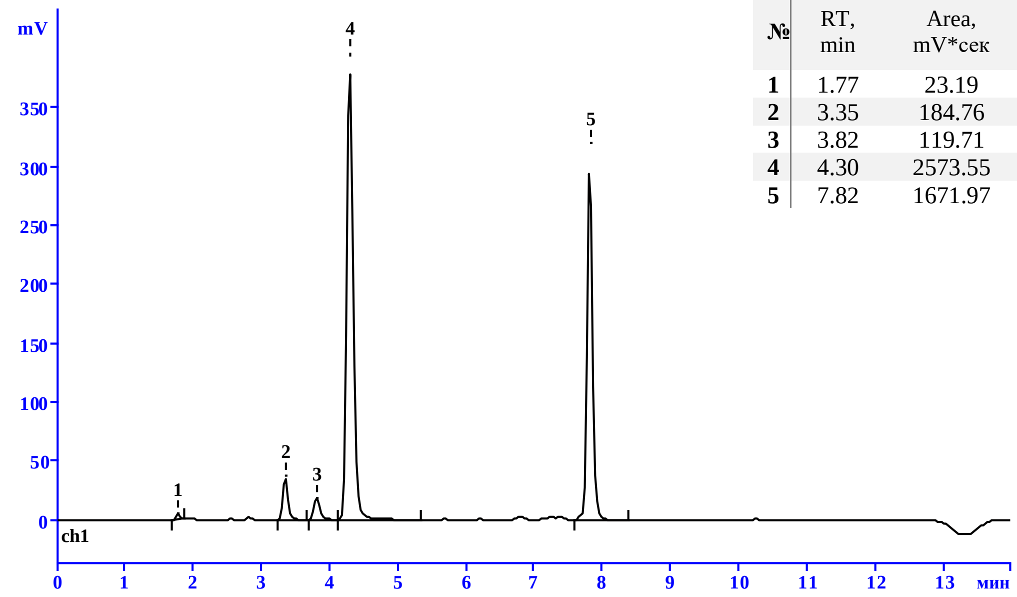

7-Me-dGuo+Hyp+P_i – 1.5:1:1 – t equilibrium

№	RT, min	Area, mV*сек	Equilibrium concentration, µM	Name
**1**	1.77	23.19	-	-
**2**	3.35	184.76	19.78	Hyp
**3**	3.82	119.71	-	-
**4**	4.30	2573.55	316.04	7-MeGua
**5**	7.82	1671.97	180.22	dIno

Fugure S10.4. **3:1:1 ratio.**

a) tinitial

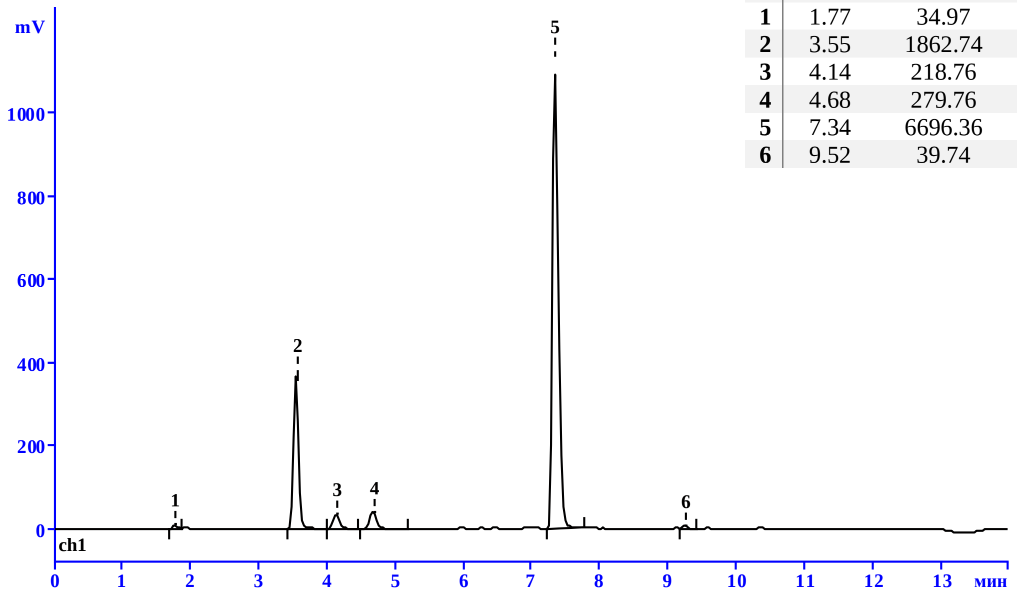

7-MedGuo+Hyp+P_i – 3:1:1 – t_0

№	RT, min	Area, mV*сек	Equilibrium concentration, µM	Name
**1**	1.77	34.97	-	-
**2**	3.55	1862.74	200.00	Hyp
**3**	4.14	218.76	-	-
**4**	4.68	279.76	34.61	7-Me-Gua
**5**	7.34	6696.36	300.00	7-MedGuo
**6**	9.52	39.74	-	-

b) t$_{equilibrium}$

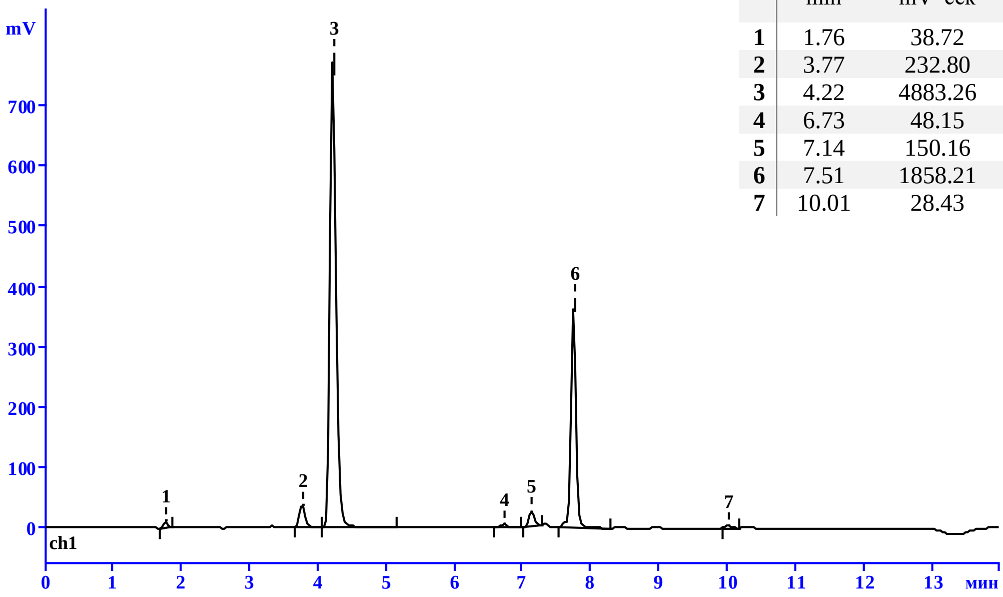

7-Me-dGuo+Hyp+P$_i$ – 3:1:1 – t $_{equilibrium}$

№	RT, min	Area, mV*сек	Equilibrium concentration, µM	Name
**1**	1.76	38.72	-	-
**2**	3.77	232.80	-	-
**3**	4.22	4883.26	621.16	7-MeGua
**4**	6.73	48.15	-	-
**5**	7.14	150.16	13.45	7-MedGuo
**6**	7.51	1858.21	200.00	dIno
**7**	10.01	28.43	-	-

2′-deoxyinosine from 7-methyl-2′-deoxyguanosine (7-Me-dGuo+N^6-Bn-2-NH$_2$-Ade+P$_i$, analytical method)

To a reaction sample solution (1 mL, Table S11) were added 1 U of PNP *E. coli*. The reaction mixture was incubated at 37°C, pH 7.5, and was monitored by HPLC.

Table S11. Volumes of stock and buffer solutions taken for 1 mL of a rection mixture (pH 7.5).

1 mM 7-Me-dGuo stock solution	1 mM N^6-Bn-2-NH$_2$-Ade stock solution	5 mM phosphate	50 mM Tris - HCl	$C_{7\text{-MedGuo}}$:$C_{N6\text{-Bn-2-NH2-Ade}}$:$C_P$
200 µL	150 µL	40 µL	610 µL	1:1.5:1
300 µL	100 µL	10 µL	590 µL	1.5:1:0.25
300 µL	100 µL	40 µL	560 µL	1.5:1:1
600 µL	100 µL	40 µL	260 µL	3:1:1

HPLC-analysis of transglycosylation: Cosmosil 5C18-MS-II, 4.6*150 mm, 5µm, 120 Å, (Nacalai Tesque, Inc. (Japan)), 20°C, elution in modified mobile phase: linear gradient of acetonitrile in 0.06% TFA/deionized water from 2 to 12% for 10 min (flushing with 12-80% acetonitrile-0.06% TFA/deionized water for 10-10.1 min, then 80-2% for 10.1-10.8 min) at flow rate 1 ml/min with UV detection at wavelength 283 nm, injection volume 20 µL.

Fugure S11.1. **1.5:1:0.25 ratio**

a) $t_{initial}$

7-MedGuo+ N^6-Bn-2-NH$_2$-Ade +P$_i$ – 1.5:1:0.25 – t_0

№	RT, min	Area, mV*сек	Equilibrium concentration, μM	Name
**1**	4.10	58.60	-	-
**2**	4.61	52.49	6.01	7-MeGua
**3**	6.78	2614.16	300.00	7-MedGuo
**4**	14.75	3795.09	200.00	N^6-Bn-2-NH$_2$-Ade

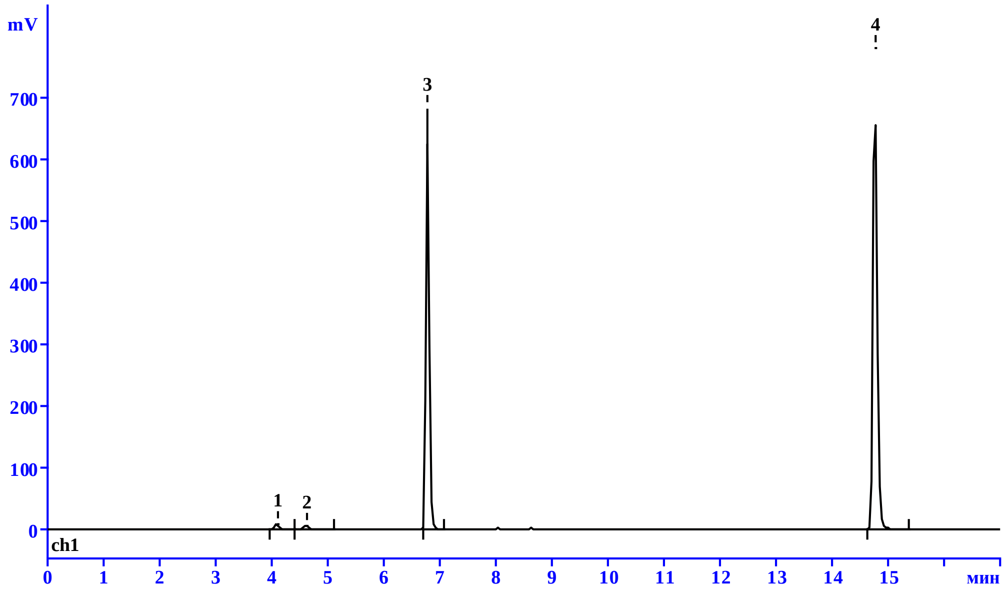

b) $t_{equilibrium}$

7-Me-dGuo+ N^6-BnGua +P$_i$ – 1.5:1:0.25 – t $_{equilibrium}$

№	RT, min	Area, mV*сек	Equilibrium concentration, μM	Name
**1**	3.94	58.32	-	-
**2**	4.38	1990.91	293.12	7-MeGua-
**3**	6.46	112.30	12.89	7-MedGuo
**4**	15.12	2889.98	200.00	N^6-Bn-2-NH$_2$-dAdo

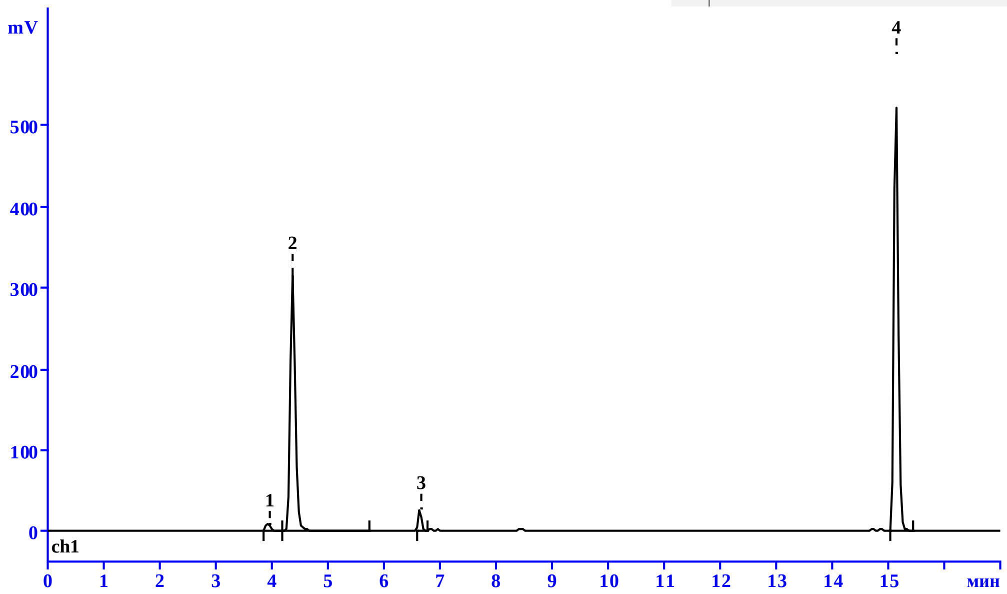

Fugure S11.2. **1.5:1:1 ratio**

a) $t_{initial}$

7-MedGuo+ *N*[6]-BnGua +P_i – 1.5:1:1 – t_0

№	RT, min	Area, mV*сек	Equilibrium concentration, µM	Name
**1**	4.04	59.18	-	-
**2**	4.58	71.13	8.14	7-MeGua
**3**	6.80	2625.63	300.00	7-MedGuo
**4**	14.83	3604.59	200.00	*N*[6]-Bn-2-NH_2-Ade

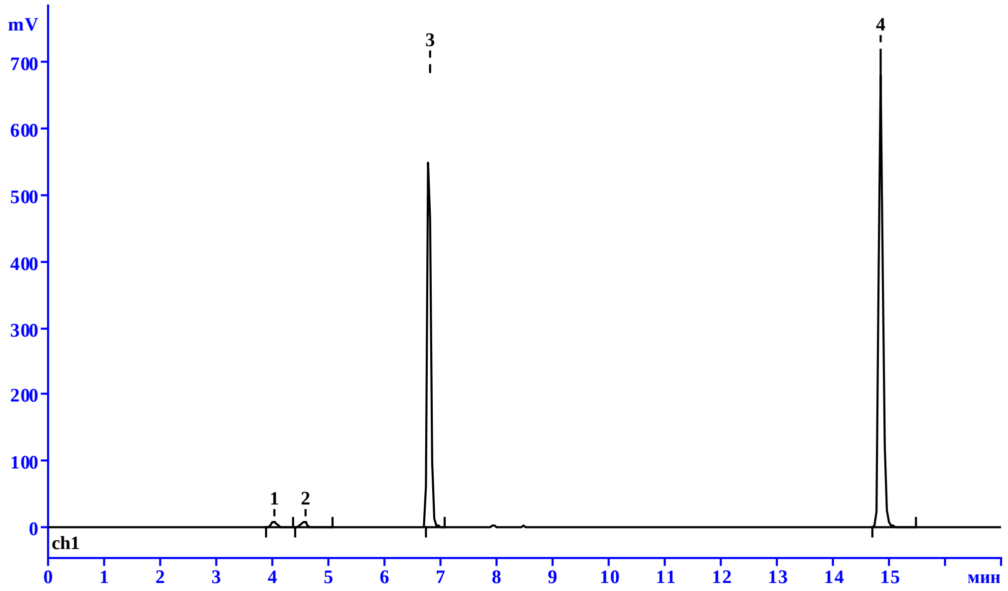

b) $t_{equilibrium}$

7-Me-dGuo+ *N*[6]-BnGua +P_i – 1.5:1:1 – t $_{equilibrium}$

№	RT, min	Area, mV*сек	Equilibrium concentration, µM	Name
**1**	3.90	59.71	-	-
**2**	4.39	2138.06	308.14	7-MeGua
**3**	14.69	30.04	1.67	*N*[6]-Bn-2-NH_2-Ade
**4**	14.95	2735.33	198.33	*N*[6]-Bn-2-NH_2-dAdo

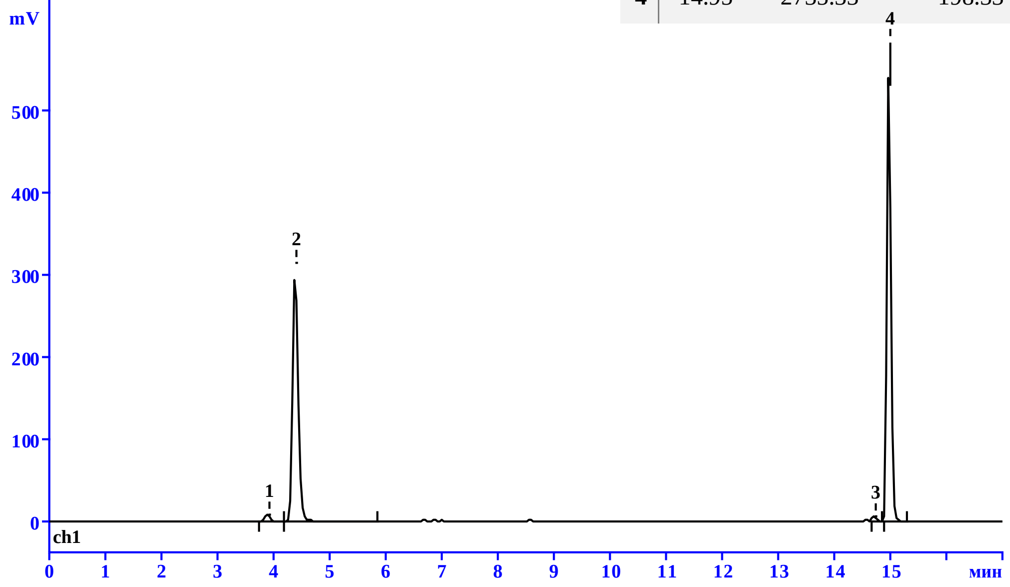

Figure S12

*Phosphorolysis of ribonucleoside **1a**.*

To the suspension of ribonucleoside **1a** (62 mg 0.14 mmol) in KH_2PO_4 buffer (50 mM, pH 7.5, 10 mL), *E.coli* PNP (1.92 U) was added. The mixture was neatly agitated at 30°C. On the next day *E.coli* alkaline phosphatase and (5.2 U) and $MgSO_4$ (18 mg) were added and the reaction mixture was left to stay for 4 days at ambient temperature. The mixture was then concentrated in vacuum to a volume *ca*. 5 ml and kept at 0°C overnight. The precipitate was centrifuged, washed with cold deionized water (μQ, 5°C, 4× X 2.5 ml) and dried in vacuum desiccator over P_2O_5 to give product **3a** in 60% yield.

a) $t_{initial}$

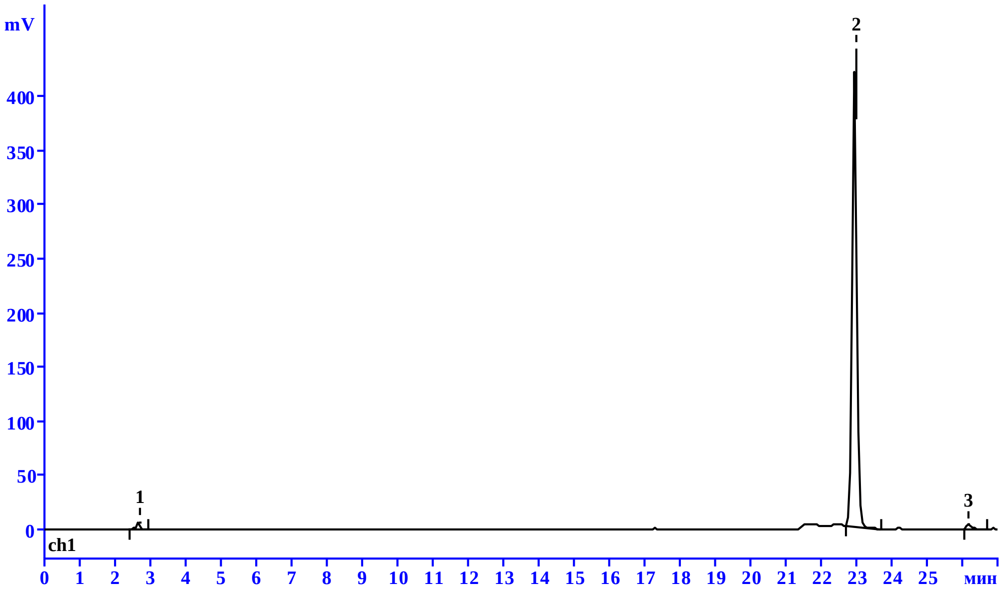

b) t_1 after 3.5 days

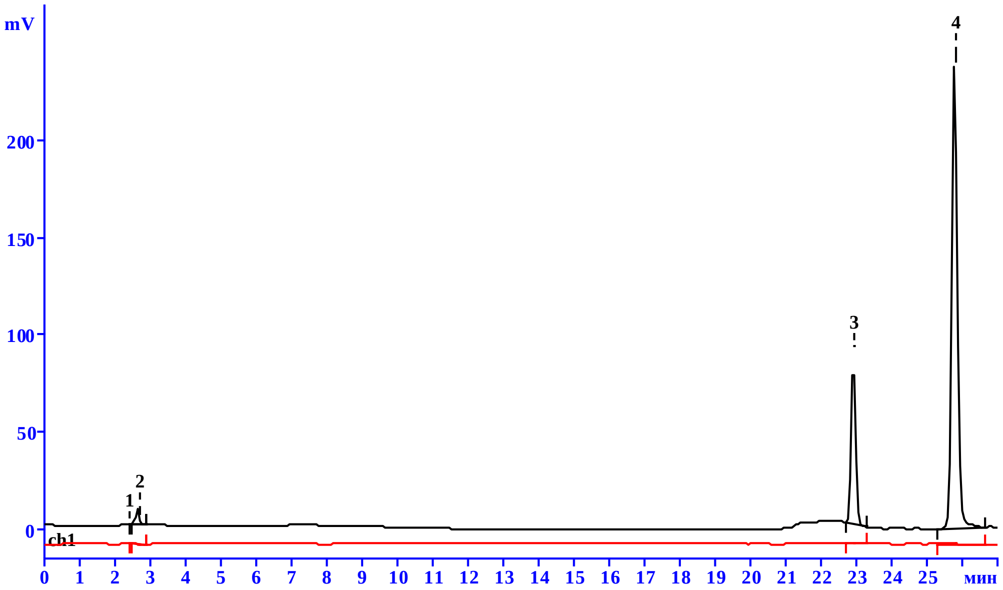

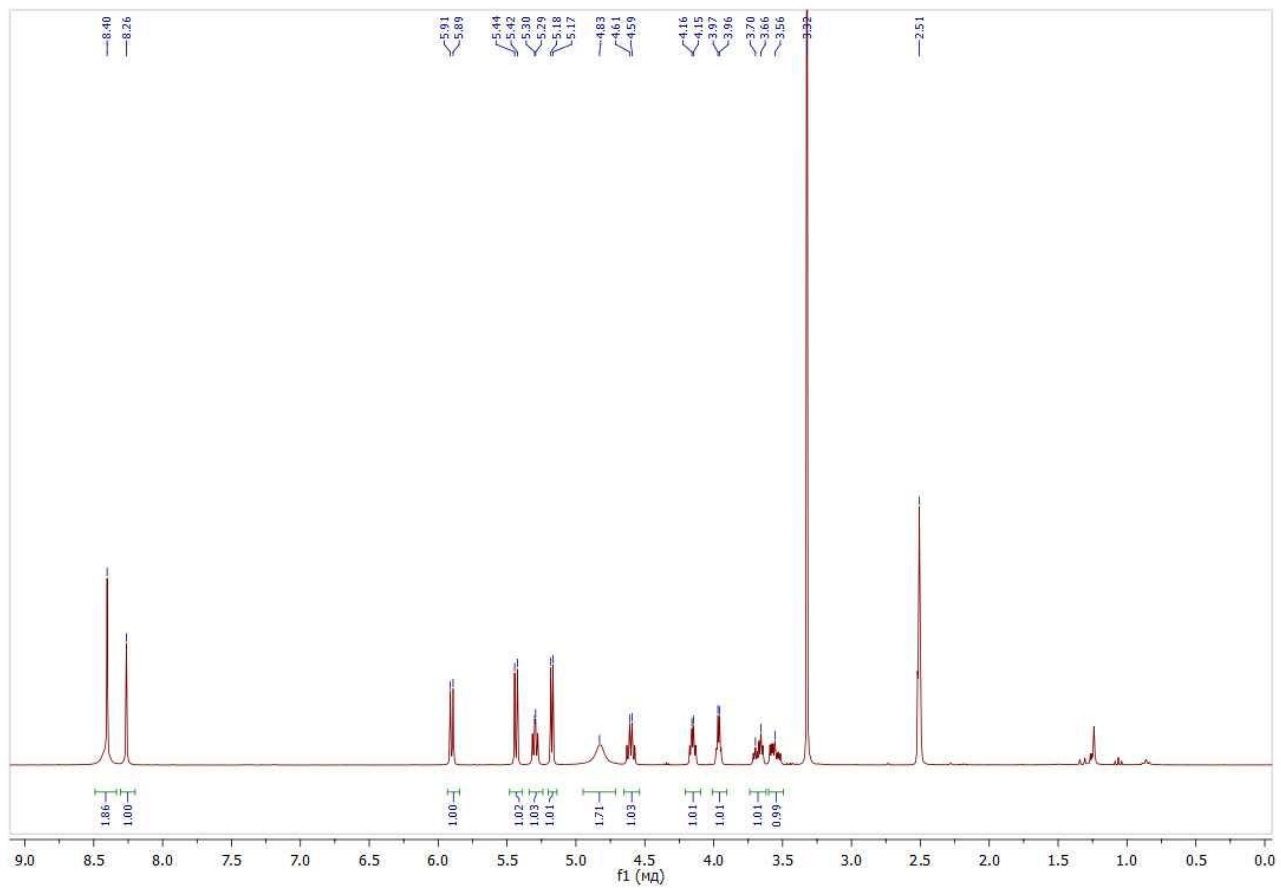

Fig. S13.1. ^{1}H-NMR spectrum (300 *MHz*, DMSO-d_6) of N^6-(2,3,4,5,6-pentafluorobenzyl)-adenosine (**1a**) at 293K.

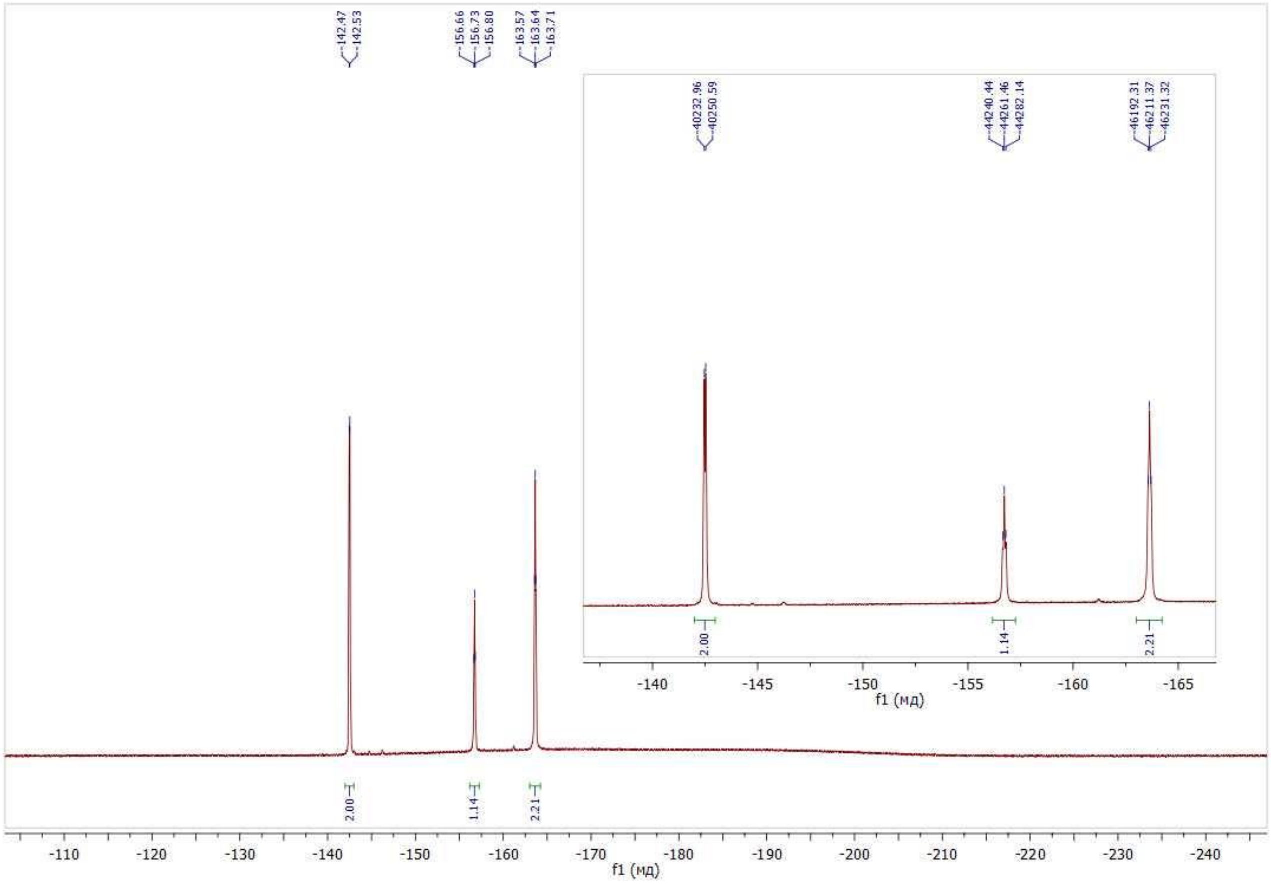

Fig. S13.2. ^{19}F-NMR spectrum (282 *MHz*, DMSO-d_6) of N^6-(2,3,4,5,6-pentafluorobenzyl)adenosine (**1a**) at 293K.

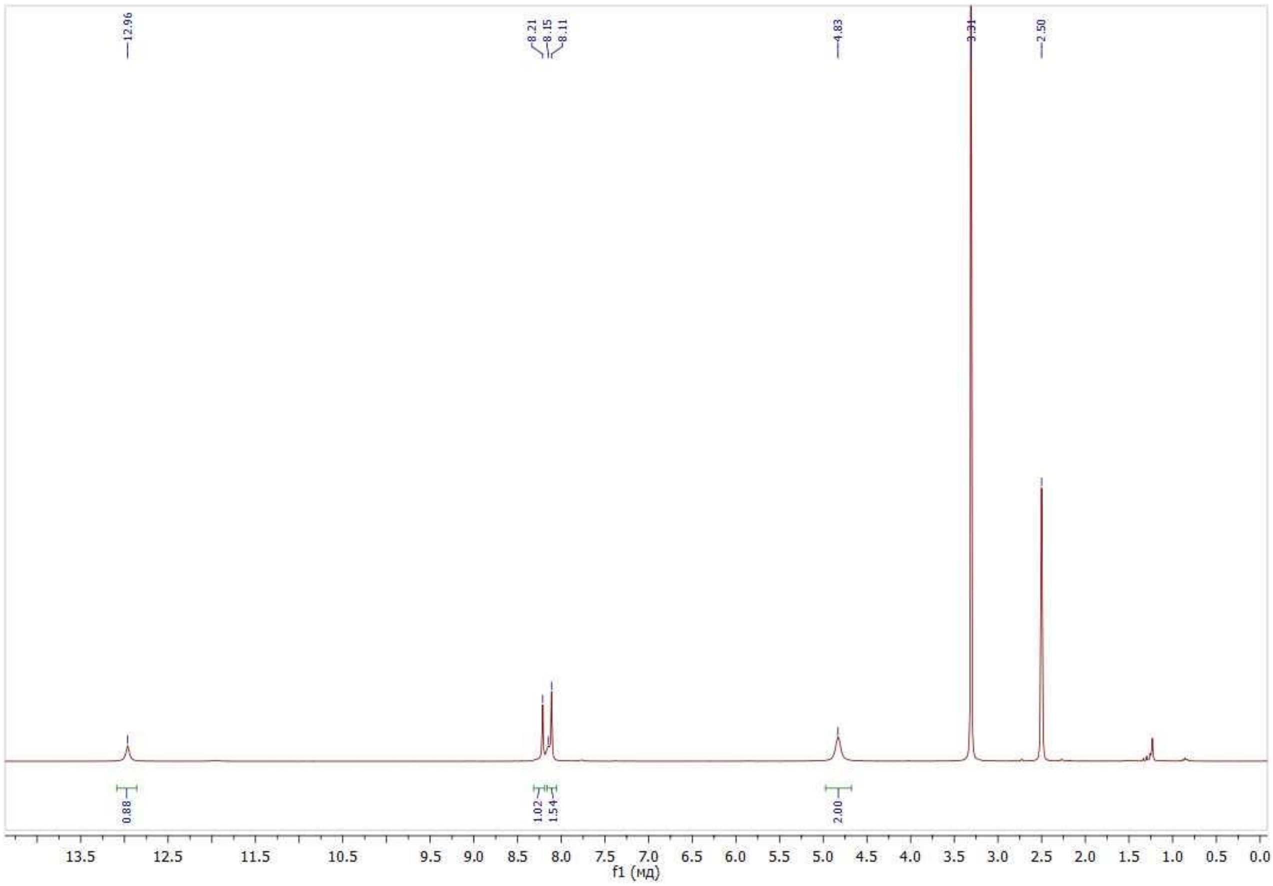

Fig. S14.1. ^{1}H-NMR spectrum (300 *MHz*, DMSO-d_6) of N^6-(2,3,4,5,6-pentafluorobenzyl)-adenine (**3a**) at 293K

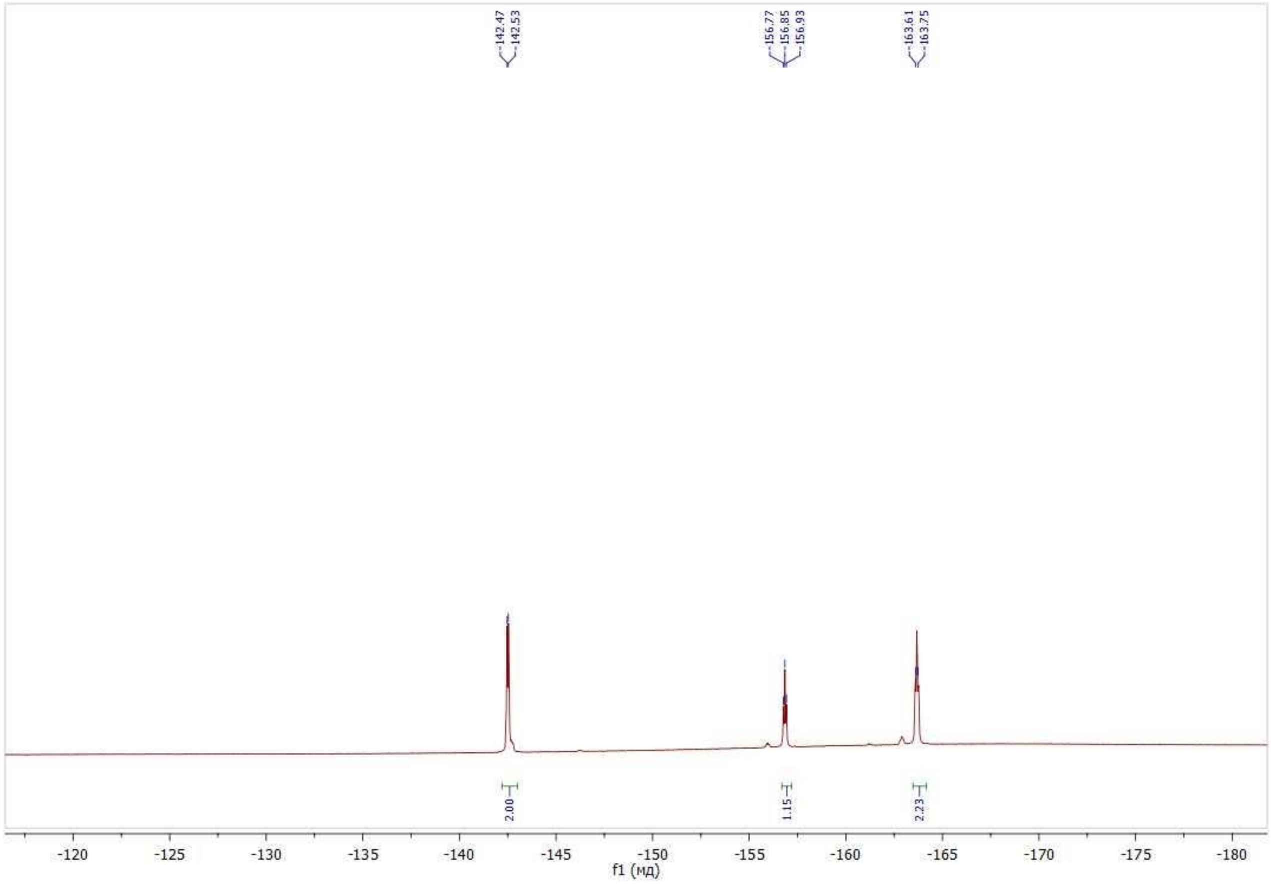

Fig. S14.2. ^{19}F-NMR spectrum (282 *MHz*, DMSO-d_6) of N^6-(2,3,4,5,6-pentafluorobenzyl)adenine (**3a**) at 293K

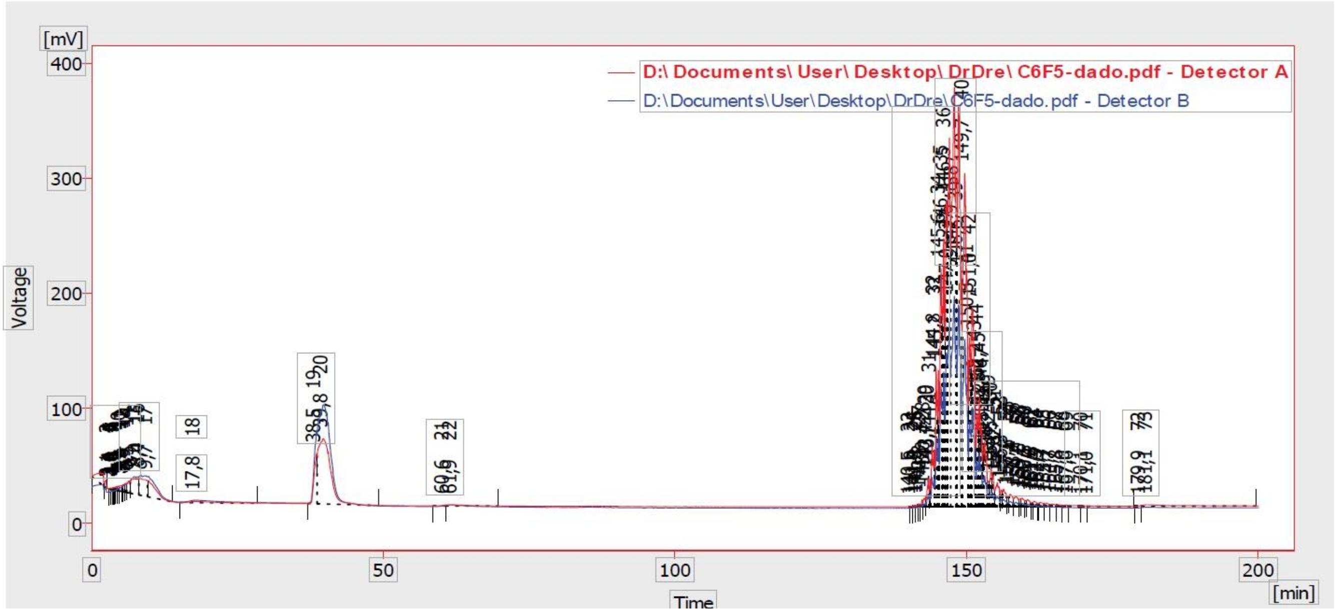

Fig. S15.1. Chromatographic purification of N^6-(2,3,4,5,6-pentafluorobenzyl)-2′-deoxyadenosine (**6a**) at 293K on C_{18}-silica-gel in 0-55% EtOH gradient in H_20. Detection by two-channel UV-detector TOY18DAD400H, channel A – registration at wavelength 267 nm, channel B - registration at wavelength 283 nm.

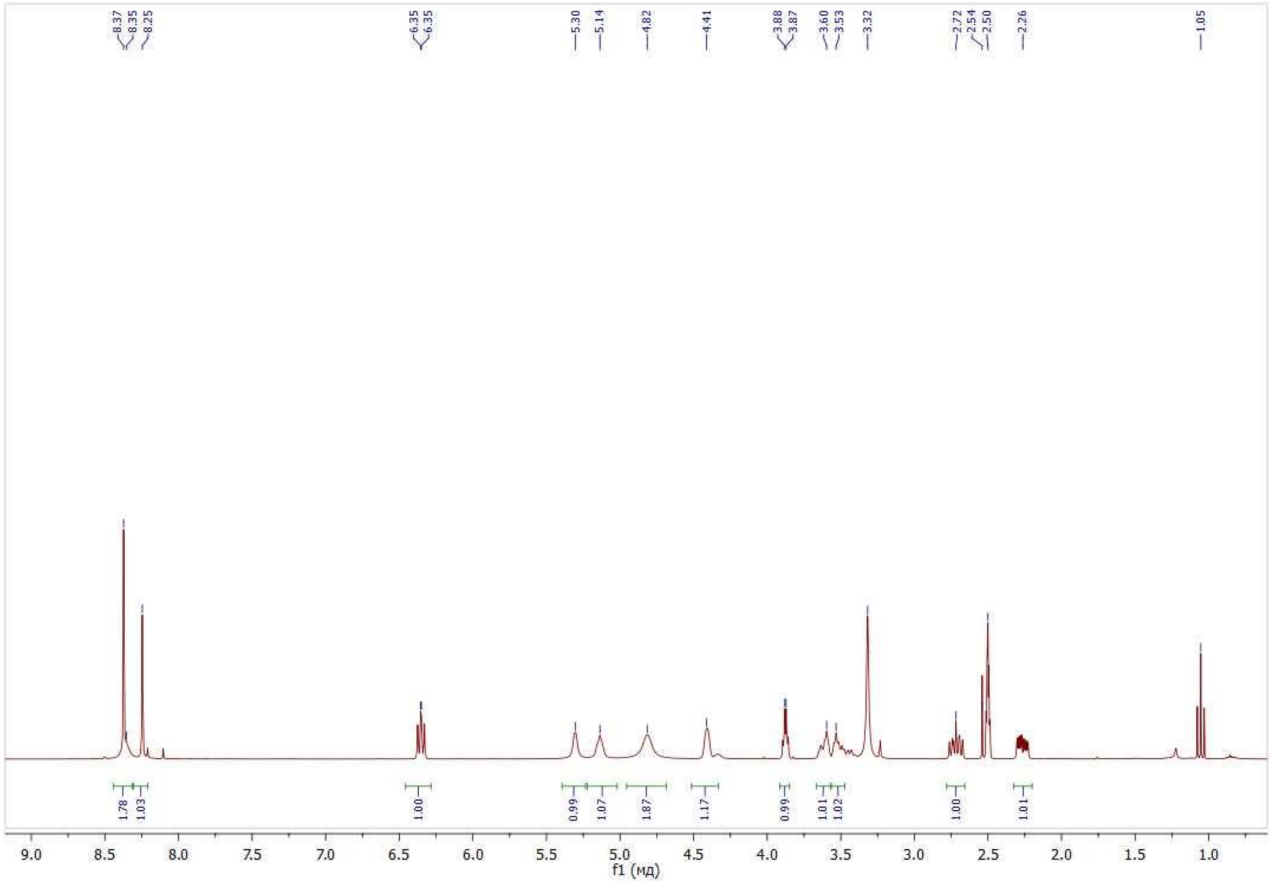

Fig. S15.2. ^{1}H-NMR spectrum (300 *MHz*, DMSO-d_6) of N^6-(2,3,4,5,6-pentafluorobenzyl)-2′-deoxyadenosine (**6a**) at 293K.

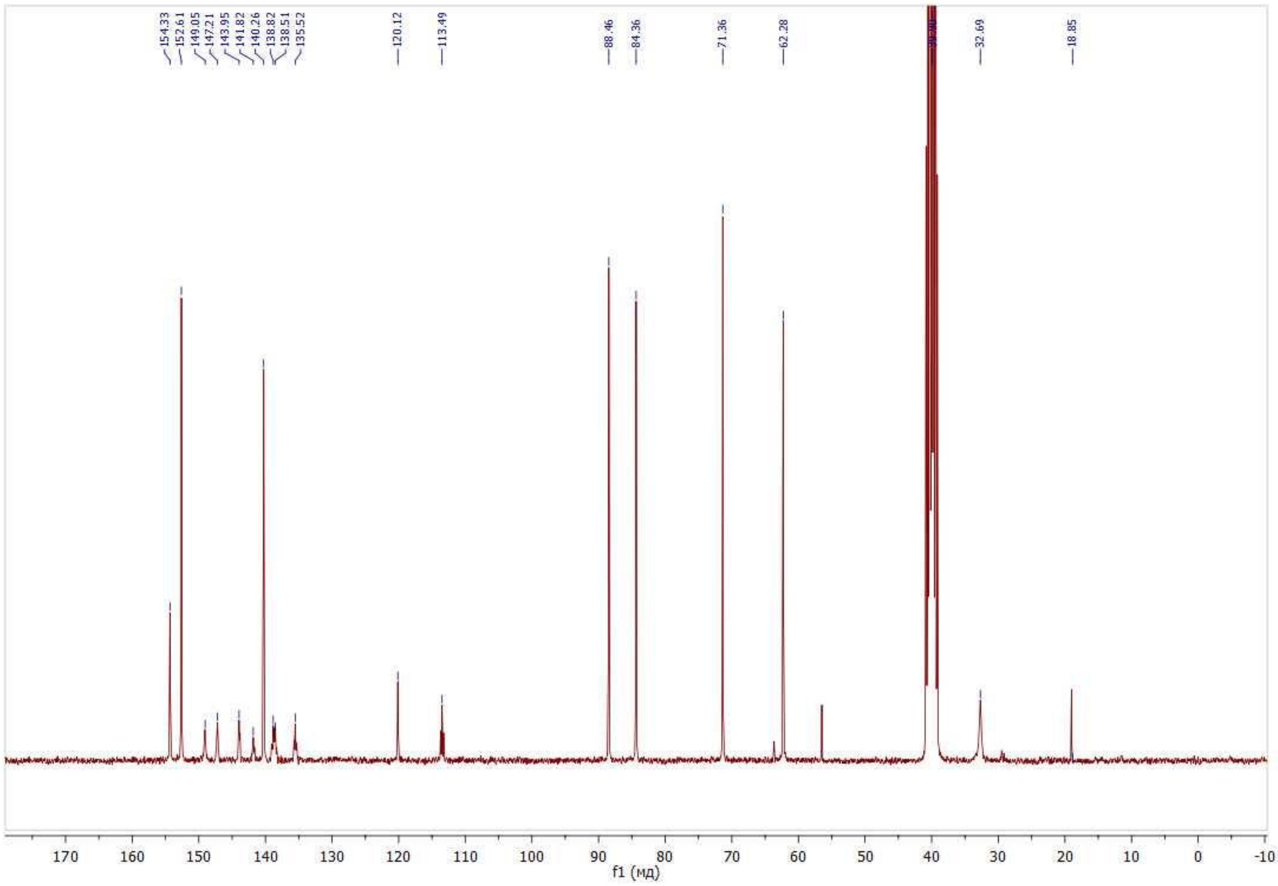

Fig. S15.3. ^{13}C-NMR spectrum (75 *MHz*, DMSO-d_6) of N^6-(2,3,4,5,6-pentafluorobenzyl)-2′-deoxyadenosine (**6a**) at 293K.

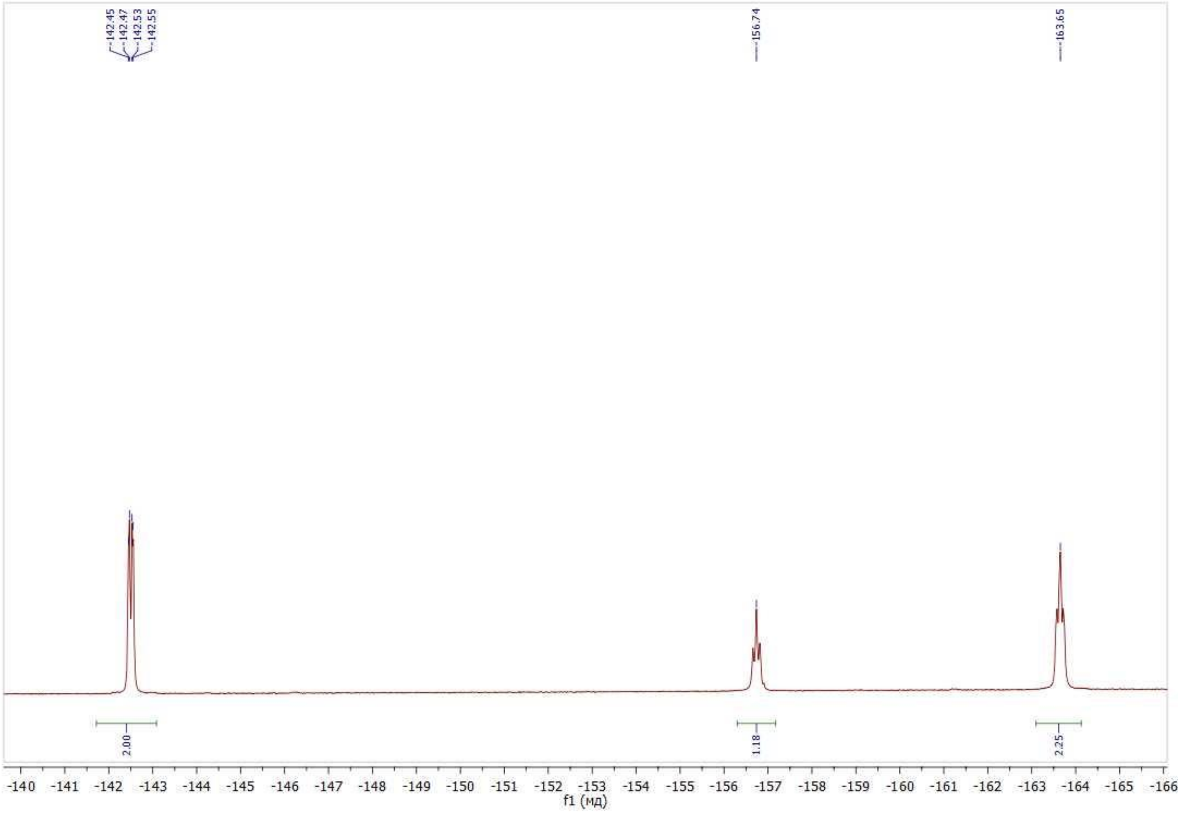

Fig. S15.4. ^{19}F-NMR spectrum (282 *MHz*, DMSO-d_6) of N^6-(2,3,4,5,6-pentafluorobenzyl)-2’-deoxyadenosine (**6a**) at 293K.

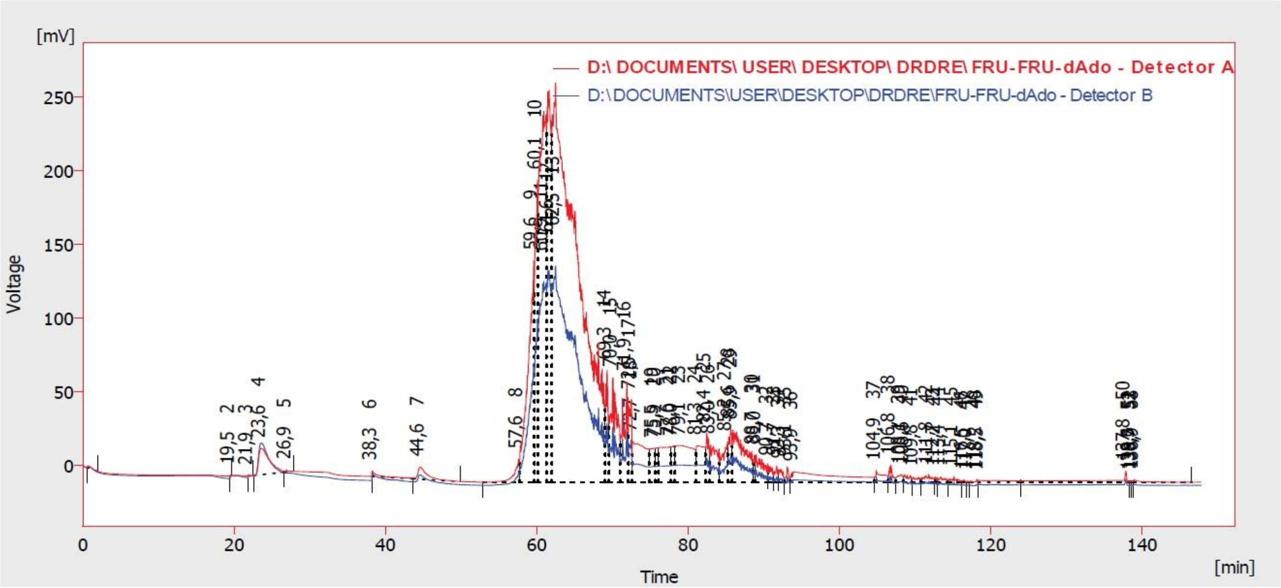

Fig. S16.1. Chromatographic purification of N^6-furfuryl-2′-deoxyadenosine (**6b**) at 293K on C_{18}-silica-gel in 0-20% EtOH gradient in $H_2$0. Detection by two-channel UV-detector TOY18DAD400H, channel A – registration at wavelength 267 nm, channel B - registration at wavelength 283 nm.

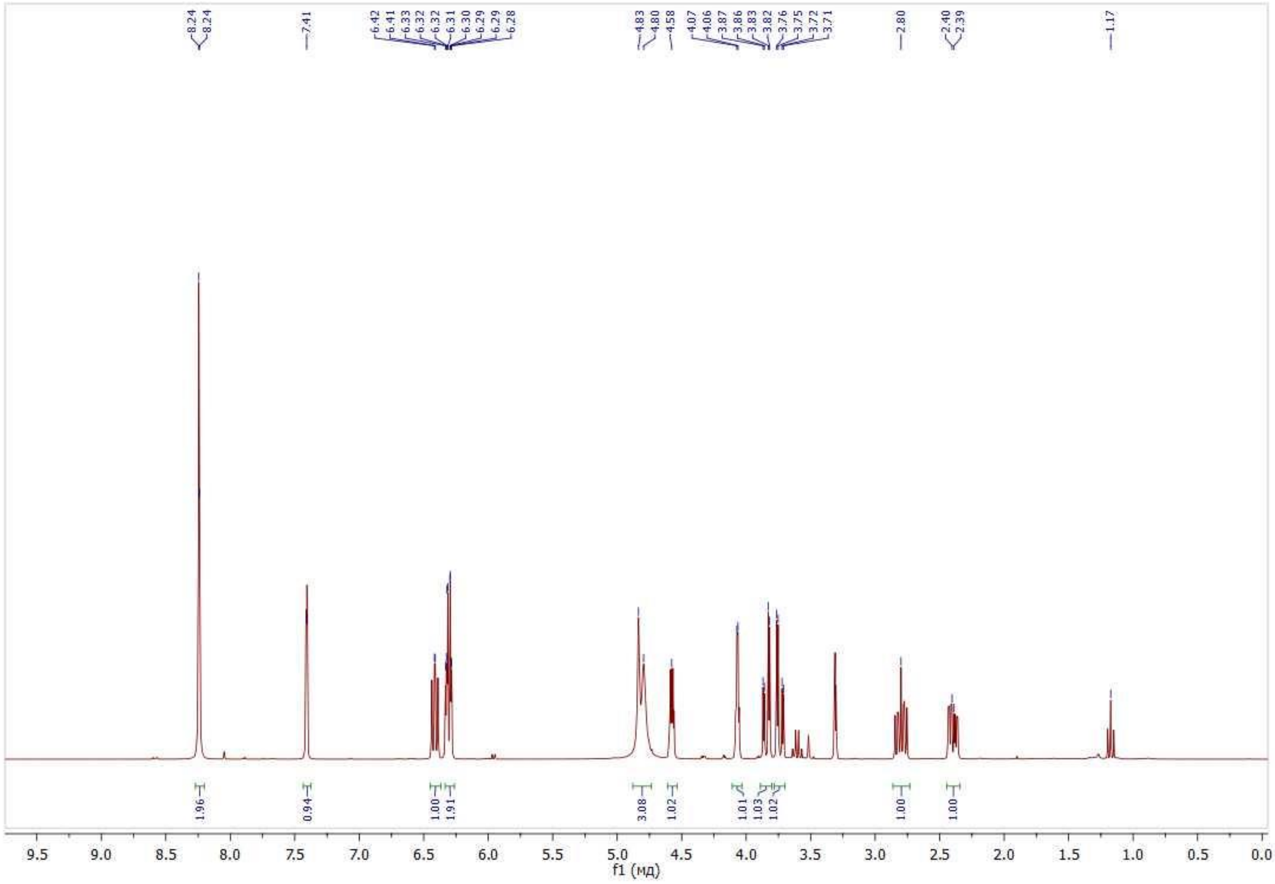

Fig. S16.2. ^{1}H-NMR spectrum (300 *MHz*, CD_3OD) of *N*^{6}-furfuryl-2′-deoxyadenosine (**6b**) at 293K.

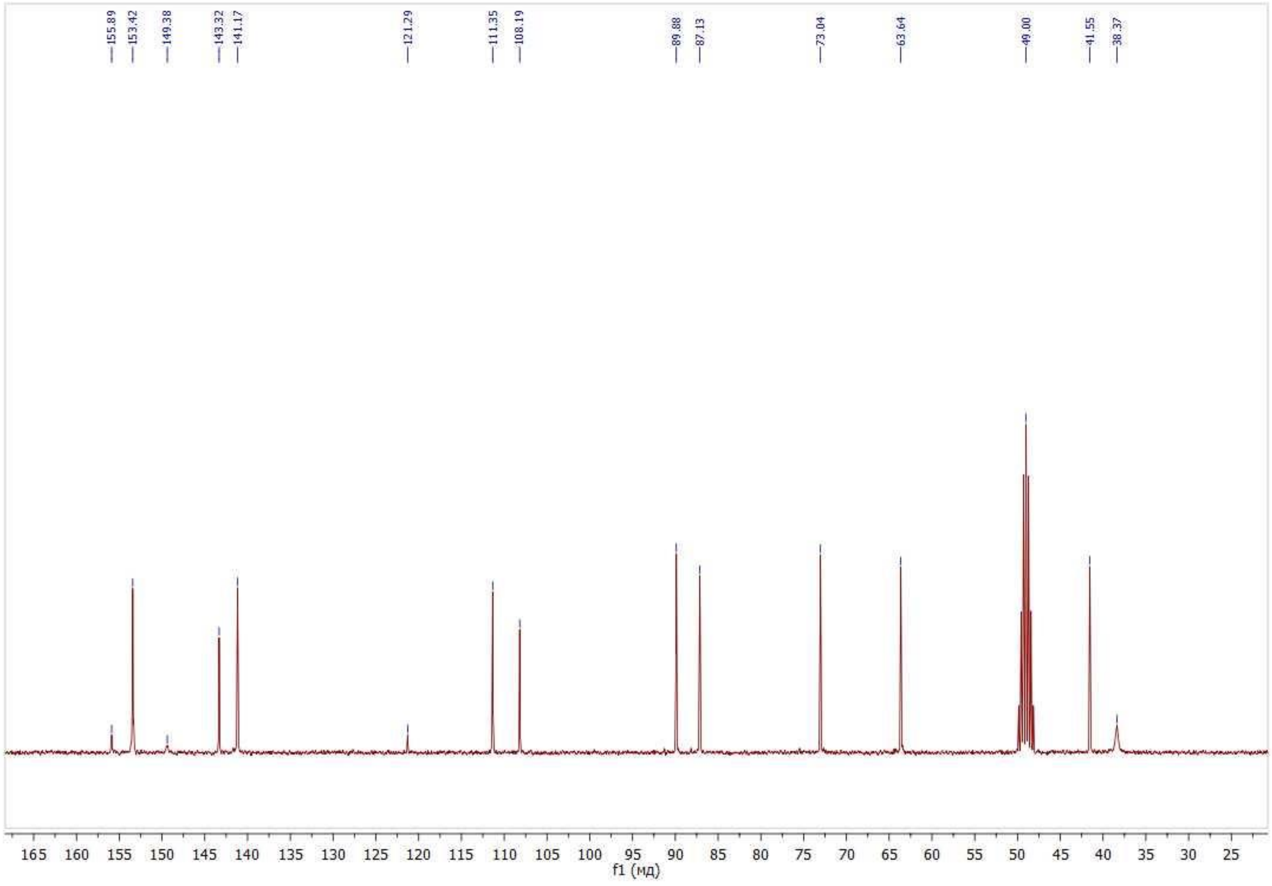

Fig. S16.3. ^{13}C-NMR spectrum (75 *MHz*, CD_3OD) of *N*^{6}-furfuryl-2′-deoxyadenosine (**6b**) at 293K.

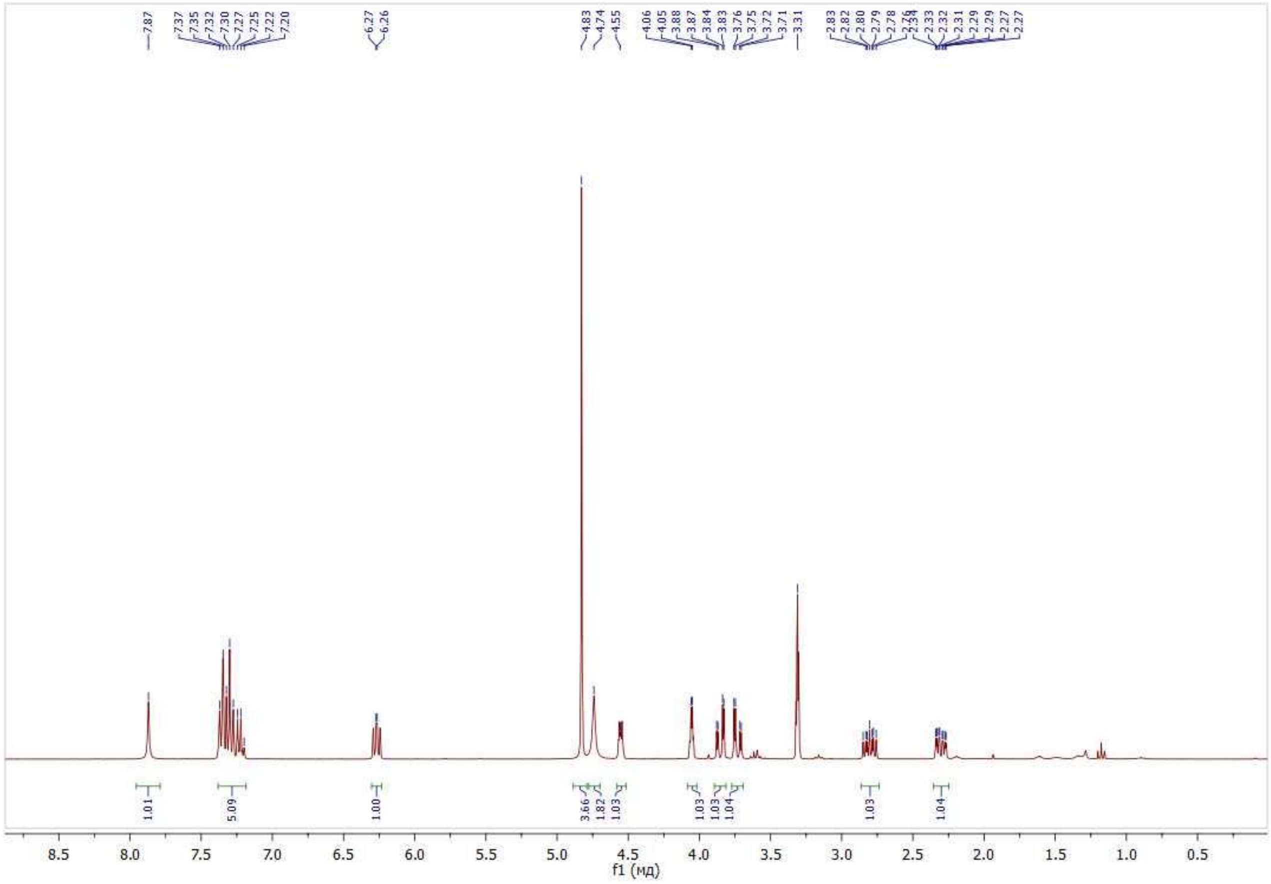

Fig. S17.1. ^{1}H-NMR spectrum (300 *MHz*, CD_3OD) of *N*^{6}-benzyl-2-amino-2′-deoxyadenosine (**6c**) at 293K.

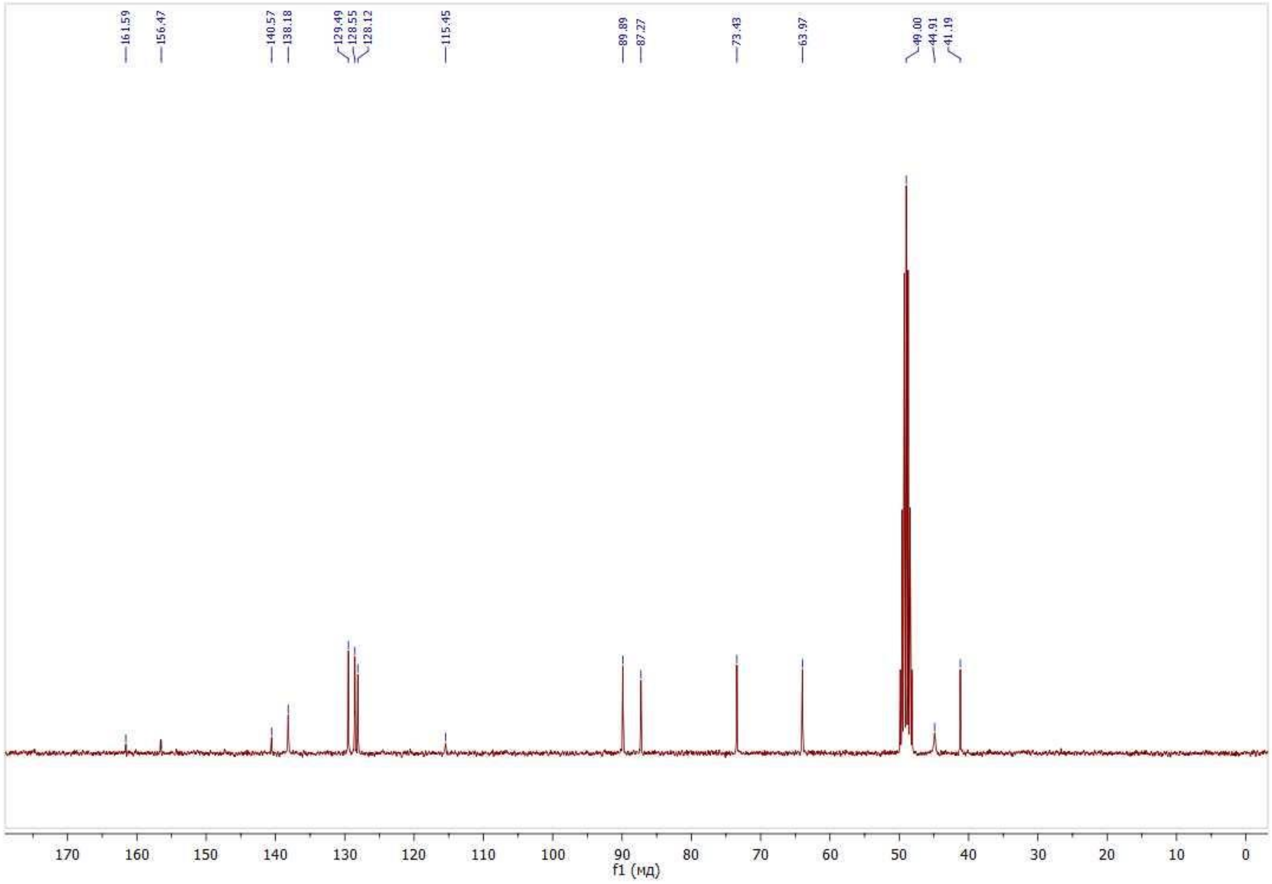

Fig. S17.2. ^{13}C-NMR spectrum (75 M*Hz*, CD_3OD) of *N*^{6}-benzyl-2-amino-2′-deoxyadenosine (**6c**) at 293K.
